# The RNA-binding ubiquitin ligase MKRN1 functions in ribosome-associated quality control of poly(A) translation

**DOI:** 10.1186/s13059-019-1814-0

**Published:** 2019-10-22

**Authors:** Andrea Hildebrandt, Mirko Brüggemann, Cornelia Rücklé, Susan Boerner, Jan B. Heidelberger, Anke Busch, Heike Hänel, Andrea Voigt, Martin M. Möckel, Stefanie Ebersberger, Anica Scholz, Annabelle Dold, Tobias Schmid, Ingo Ebersberger, Jean-Yves Roignant, Kathi Zarnack, Julian König, Petra Beli

**Affiliations:** 1 Institute of Molecular Biology (IMB), Ackermannweg 4, 55128 Mainz, Germany; 2 Buchmann Institute for Molecular Life Sciences (BMLS), Goethe University, Max-von-Laue-Str. 15, 60438 Frankfurt am Main, Germany; 3 Faculty of Medicine, Institute of Biochemistry I, Goethe University Frankfurt, Theodor-Stern-Kai 7, 60590 Frankfurt am Main, Germany; 4 Department for Applied Bioinformatics, Institute of Cell Biology and Neuroscience, Goethe University Frankfurt, Max-von-Laue-Str. 13, 60438 Frankfurt am Main, Germany; 5 Senckenberg Biodiversity and Climate Research Centre (BiK-F), Georg-Voigt-Straße 14-16, 60325 Frankfurt am Main, Germany; 6 Center for Integrative Genomics, Faculty of Biology and Medicine, University of Lausanne, Génopode Building, CH-1015 Lausanne, Switzerland

**Keywords:** MKRN1, Ubiquitylation, RNA binding, Ribosome-associated quality control, Poly(A), iCLIP, Ubiquitin remnant profiling, Translation

## Abstract

**Background:**

Cells have evolved quality control mechanisms to ensure protein homeostasis by detecting and degrading aberrant mRNAs and proteins. A common source of aberrant mRNAs is premature polyadenylation, which can result in non-functional protein products. Translating ribosomes that encounter poly(A) sequences are terminally stalled, followed by ribosome recycling and decay of the truncated nascent polypeptide via ribosome-associated quality control.

**Results:**

Here, we demonstrate that the conserved RNA-binding E3 ubiquitin ligase Makorin Ring Finger Protein 1 (MKRN1) promotes ribosome stalling at poly(A) sequences during ribosome-associated quality control. We show that MKRN1 directly binds to the cytoplasmic poly(A)-binding protein (PABPC1) and associates with polysomes. MKRN1 is positioned upstream of poly(A) tails in mRNAs in a PABPC1-dependent manner. Ubiquitin remnant profiling and in vitro ubiquitylation assays uncover PABPC1 and ribosomal protein RPS10 as direct ubiquitylation substrates of MKRN1.

**Conclusions:**

We propose that MKRN1 mediates the recognition of poly(A) tails to prevent the production of erroneous proteins from prematurely polyadenylated transcripts, thereby maintaining proteome integrity.

## Introduction

During gene expression, quality control pathways monitor each step to detect aberrant mRNAs and proteins. These mechanisms ensure protein homeostasis and are essential to prevent neurodegenerative diseases [1]. A common source of aberrant mRNAs is premature polyadenylation, often in combination with mis-splicing, which results in truncated non-functional protein products [2]. Therefore, mechanisms are in place that recognize such homopolymeric adenosine (poly(A)) sequences and abrogate their translation [3].

In eukaryotes, ribosomes that terminally stall for diverse reasons during translation are detected by ribosome-associated quality control (RQC) (reviewed in [4, 5]). Upon splitting of the 60S and 40S ribosomal subunits, the RQC complex assembles on the 60S subunit to initiate the release and rapid degradation of the truncated tRNA-bound polypeptide. The E3 ubiquitin ligase Listerin (LTN1) modifies the truncated polypeptide with K48-linked ubiquitin chains to target it for degradation in a p97-dependent manner through the proteasome [3, 6, 7]. Whereas peptide release and ribosome recycling by the RQC complex are relatively well understood, less is known about the mechanisms that promote poly(A) recognition and initial ribosome stalling.

Several recent studies demonstrated a role for the RNA-binding E3 ubiquitin ligase ZNF598 in initiating RQC for prematurely polyadenylated mRNAs [8–10]. It was suggested that ZNF598 senses the translation of poly(A) segments through binding of the cognate lysine tRNAs [9]. In addition, ZNF598 recognizes the collided di-ribosome structure that arises when a trailing ribosome encounters a slower leading ribosome [11]. This is followed by site-specific, regulatory ubiquitylation of the 40S ribosomal proteins RPS10 and RPS20 by ZNF598. In addition to ZNF598, the 40S ribosomal subunit-associated protein RACK1 was shown to regulate ubiquitylation of RPS2 and RPS3 upstream of ribosomal rescue [10].

Makorin Ring Finger Protein 1 (MKRN1) belongs to a family of evolutionary conserved RNA-binding E3 ubiquitin ligases. Up to four paralogs exist in vertebrates (MKRN1–4), which combine a RING domain with one or more CCCH zinc finger domains [12, 13] (Additional file 1: Figure S1). MKRN1 has been implicated in the regulation of telomere length, RNA polymerase II transcription, and the turnover of tumor suppressor protein p53 and cell cycle regulator p21 [14–17], but its RNA-related functions remain poorly understood. A study in mouse embryonic stem cells (mESC) reported its interaction with hundreds of mRNAs as well as multiple RNA-binding proteins (RBPs), including the cytoplasmic poly(A)-binding protein (PABP) PABPC1, IGF2BP1, and ELAVL1 [18]. The interaction with the poly(A)-binding protein was further corroborated in human HEK293 cells [19]. The same study demonstrated that a shortened isoform of MKRN1 controls local translation via its PABP-interacting motif 2 (PAM2 motif) in rat neurons [19]. In line with a role in translation, MKRN1 was found in association with ribosomes, from which it could be released together with PABP and other proteins by RNase digestion [20].

The presence of several RNA binding domains and a RING domain in MKRN1 prompted us to study the function of MKRN1 in human cells. Here, we find that MKRN1 is a novel factor in RQC. We show that MKRN1 is recruited to A-rich sequences in mRNAs in a PABPC1-dependent manner. MKRN1 depletion abrogates ribosome stalling at A-rich sequences and results in reduced ubiquitylation of RPS10 and PABPC1. We therefore propose that MKRN1 acts as a first line of defense against poly(A) translation at the mRNA level to prevent premature polyadenylation and the production of erroneous proteins.

## Results

### MKRN1 interacts with PABPC1 and other RBPs

In order to learn about potential functions, we characterized the protein interaction profile of MKRN1 in HEK293T cells. To this end, we used affinity purification (AP) coupled to stable isotope labelling with amino acids in cell culture (SILAC)-based quantitative mass spectrometry (MS) using GFP-MKRN1^wt^ or GFP as a bait. We identified 53 proteins that were significantly enriched in GFP-MKRN1^wt^ compared to the control APs (false discovery rate [FDR] < 5%, combined ratios of three independent experiments). Almost all identified interactors were previously found in association with polyadenylated transcripts (Gene Ontology [GO] term “poly(A) RNA binding,” Additional file 1: Figure S2A).

**Fig. 1:**
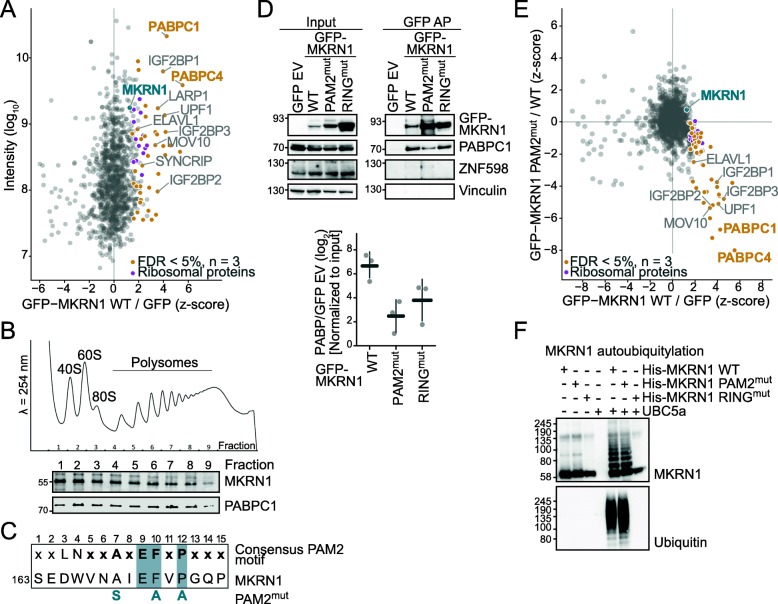
MKRN1 interacts with PABPC1 and other regulators of translation and RNA stability. **a** Protein interactome of GFP-MKRN1^wt^ in HEK293T cells analyzed by quantitative MS-based proteomics. Combined SILAC ratios (*n* = 3 replicates) after *z*-score normalization are plotted against log_10_-transformed intensities. 1100 protein groups were quantified in at least two out of three replicate experiments. MKRN1 and significant interactors are highlighted (FDR < 5%). **b** MKRN1 and PABPC1 associate with polysomes. A 10–50% sucrose gradient of cycloheximide-treated HEK293T cell extracts. Shown are the Western blot analyses of individual gradient fractions with antibodies against MKRN1 and PABPC1/3 (*n* = 3 replicates, plus one technical replicate). UV absorbance was measured at *λ* = 254 nm. Replicates and uncropped gel images are shown in Additional file 3: Figure S10A-C. **c** A PAM2 motif similar to the previously reported consensus (shown on top; Additional file 1: Figure S1B) [22] is present in MKRN1 (first amino acid position indicated on the left). Introduced mutations in MKRN1^PAM2mut^ are indicated in petrol below. Relevant positions are highlighted (Additional file 1: Figure S1B). **d** Endogenous PABPC1 interacts strongly with MKRN1^wt^ and MKRN1^RINGmut^, but only to a lesser extent with MKRN1^PAM2mut^. Western blots for endogenous PABPC1 and GFP after AP of GFP-MKRN1 (wt and mutants). Ratios of PABPC1 signal (normalized to input) in GFP-MKRN1 APs over control (GFP empty vector, EV) are shown below. Replicates 2, 3, and uncropped gel images are shown in Additional file 3: Figure S10D-F. **e** Quantitative comparison of the interactomes of GFP-MKRN1^wt^ and GFP-MKRN1^PAM2mut^ shows that PABPC1 and several other interactors are lost upon PAM2 mutation. Combined ratios of three replicates are shown in a scatter plot. Only proteins detected in at least two out of three replicates are shown. MKRN1^wt^ significant interactors (from **a**) are highlighted as in **a** (FDR < 5% in MKRN1^wt^). **f** MKRN1^WT^ and MKRN1^PAM2mut^, but not MKRN1^RINGmut^, efficiently autoubiquitylate. In vitro ubiquitylation assays with recombinant His-tagged MKRN1^wt^ and mutant proteins that were incubated with or without the E2 enzyme UBC5a, the E1 enzyme UBA1, and ubiquitin. A reaction with UBC5a only served as a control. Autoubiquitylation was analyzed by Western blot. Replicates 2, 3, and uncropped gel images are shown in Additional file 3: Figure S10G,H

In line with previous reports [18, 19, 21], we found that MKRN1 strongly interacts with the cytoplasmic poly(A)-binding proteins PABPC1 and PABPC4 (Fig. 1a, Additional file 1: Figure S2B, Additional file 2: Table S1). In addition, we detected 14 ribosomal proteins as well as four proteins that were previously shown to co-purify with ribosomes [20], including IGF2BP1, LARP1, UPF1, and ELAVL1 (Fig. 1a). We confirmed the MS results in reciprocal AP experiments with GFP-tagged PABPC1, ELAVL1, and IGF2BP1 as baits followed by Western blot for endogenous MKRN1 (Additional file 1: Figure S2C and Additional file 3: Figure S10). All detected interactions persisted in the presence of RNases (RNase A and T1), demonstrating that MKRN1 interacts with these proteins in an RNA-independent manner (Additional file 1: Figure S2C). The association is further supported by a study on the Mkrn1 ortholog in *Drosophila melanogaster*, which identified pAbp, Larp, Upf1, and Imp (IGF2BP in mammals) as interaction partners (Dold et al., bioRxiv, 10.1101/501643).

Our results suggest that MKRN1 is part of a larger mRNA ribonucleoprotein particle (mRNP) together with PABPC1 and other RBPs that are involved in translational regulation. “Nonsense-mediated decay” and “translation” were the most significantly enriched GO terms for the MKRN1 interaction partners (Biological Process, Additional file 1: Figure S2A). Furthermore, MKRN1 was clearly present in polysomal fractions, as determined in sucrose gradient centrifugation experiments. Here, it co-sedimented with PABPC1 (Fig. 1b), indicating that together with PABPC1, MKRN1 is associated with translating ribosomes. Several proteins interact with PABPC1 via a PABP-interacting motif 2 (PAM2) motif, which specifically binds to the MLLE domain present almost exclusively in PABP proteins [23, 24]. Accordingly, a previous study demonstrated that MKRN1 associates with PABP via a PAM2 motif at amino acid positions 161–193 [19]. In support of a putative functional relevance, a phylogenetic analysis illustrated that the presence and positioning of the PAM2 motif are preserved in MKRN1 orthologs across metazoans (Additional file 1: Figure S1A,B). Point mutations in the PAM2 motif of MKRN1 (GFP-MKRN1^PAM2mut^) [25] strongly compromised its interaction with PABPC1 and PABPC4 in AP experiments followed by MS or Western blot (Fig. 1c–e and Additional file 2: Table S1). Surprisingly, MKRN1^PAM2mut^ lost interaction not only with PABPC1 and PABPC4, but also with several other identified proteins (Fig. 1e), suggesting that the mutant no longer resided within the mRNPs. For comparison, we also tested a previously described point mutation in the RING domain that abolishes the E3 ubiquitin ligase function (ligase-dead, GFP-MKRN1^RINGmut^) [14]. This mutant exhibited stable interactions with PABPC1 and other interactors in the AP-MS data. In Western blot experiments, we noted a reduced interaction of MKRN1^RINGmut^ with PABPC1. A possible explanation could be the substantially increased levels of MKRN1^RINGmut^ in the cells that may skew the normalization in the Western blot experiments (Fig. 1d, Additional file 1: Figure S3 and Additional file 2: Table S1).

In order to rule out that a general loss of protein integrity of MKRN1^PAM2mut^, e.g., due to misfolding, is responsible for the observed loss of PABPC1 interaction, we tested the activity of recombinant MKRN1 protein variants in in vitro ubiquitylation assays. As expected, recombinant MKRN1^RINGmut^ was enzymatically inactive, as reflected in the complete absence of autoubiquitylation (Fig. 1f, Additional file 1: Figure S1E). In contrast, both MKRN1^wt^ and MKRN1^PAM2mut^ could autoubiquitylate with the same efficiency, evidencing that the E3 ubiquitin ligase domain of MKRN1^PAM2mut^ is still functional. Moreover, confocal microscopy of GFP-MKRN1^wt^ and GFP-MKRN1^PAM2mut^ confirmed that the PAM2 mutation did not lead to aggregation nor otherwise impaired protein localization (Additional file 1: Figure S1D). Together, this provides strong evidence that MKRN1^PAM2mut^ is not generally corrupted.

Overall, our results confirm that MKRN1 interacts with PABPC1 and suggest that both proteins associate with translating ribosomes. Loss of the MKRN1-PABPC1 interaction impairs mRNP formation, indicating that PABPC1 may be involved in recruiting MKRN1 to the mRNPs.

### MKRN1 binds to poly(A) tails and A-rich stretches in 3′ UTRs

In order to characterize the RNA binding behavior of human MKRN1 in vivo, we performed individual-nucleotide resolution UV crosslinking and immunoprecipitation (iCLIP) [26] in combination with 4-thiouridine (4SU) labeling to enhance UV crosslinking [27]. In three replicate experiments with GFP-tagged MKRN1 (GFP-MKRN1^wt^) expressed in HEK293T cells, we identified more than 4.6 million unique crosslink events, cumulating into 7331 MKRN1 binding sites (see the “Materials and methods” section; Additional file 1: Figure S4A and Table S2). These were further ranked according to the strength of MKRN1 binding, which was estimated from the enrichment of crosslink events within a binding site relative to its local surrounding as a proxy for transcript abundance (“signal-over-background,” SOB; see the “Materials and methods” section) [28]. SOB values were highly reproducible between replicates (Pearson correlation coefficients *r* > 0.72, Additional file 1: Figure S4B-G).

**Fig. 2:**
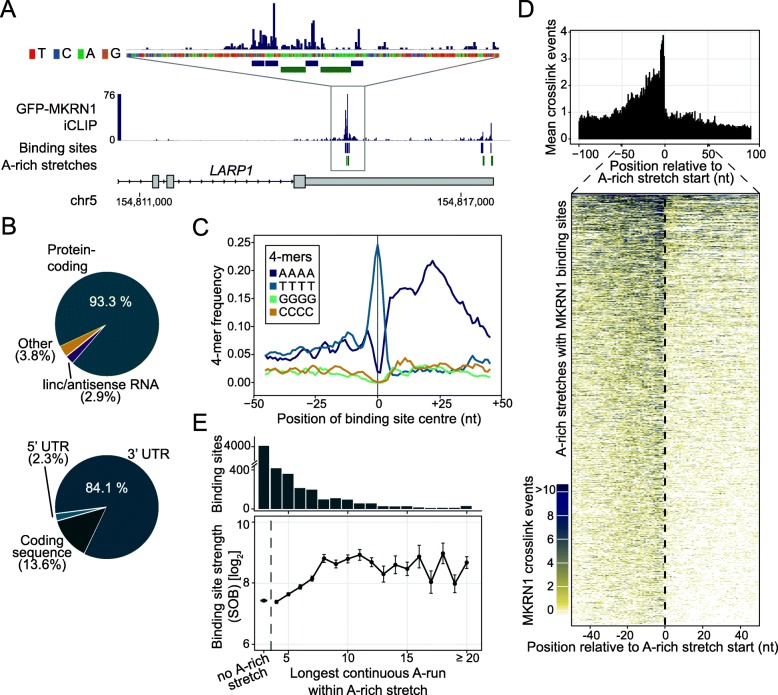
MKRN1 binds upstream of A-rich stretches in 3′ UTRs. **a** MKRN1 binds upstream of A-rich stretches in the 3′ UTR of the *LARP1* gene. Genome browser view of GFP-MKRN1 iCLIP data showing crosslink events per nt (merged replicates) together with binding sites (lilac) and associated A-rich stretches (dark green). **b** MKRN1 predominantly binds in the 3′ UTR of protein-coding genes. Pie charts summarizing the distribution of MKRN1 binding sites to different RNA biotypes (7331 binding sites, top) and different regions within protein-coding transcripts (6913 binding sites, bottom). **c** MKRN1 binding sites display a downstream enrichment of AAAA homopolymers. Frequency per nucleotide (nt) for four homopolymeric 4-mers in a 101-nt window around the midpoints of the top 20% MKRN1 binding sites (according to signal-over-background; see the “Materials and methods” section). **d** MKRN1 crosslink events accumulate upstream of A-rich stretches. Metaprofile (top) shows the mean crosslink events per nt in a 201-nt window around the start position of 1412 MKRN1-associated A-rich stretches in 3′ UTRs. Heatmap visualization (bottom) displays crosslink events per nt (see color scale) in a 101-nt window around the MKRN1-associated A-rich stretches. **e** MKRN1 binding site strength (signal-over-background, SOB) increases with the number of continuous A’s within the A-rich stretch. Mean and standard deviation of MKRN1 binding site strengths associated with A-rich stretches harboring continuous A runs of increasing length (*x*-axis). MKRN1 binding sites without associated A-rich stretches are shown for comparison on the left. Number of binding sites in each category indicated as bar chart above

Across the transcriptome, MKRN1 almost exclusively bound to protein-coding mRNAs with a strong tendency to locate in 3′ UTRs (Fig. 2a, b). Binding sites generally harbored uridine-rich tetramers (Additional file 1: Figure S5A), likely reflecting 4SU-based UV crosslinking [27]. Strikingly, the top 20% MKRN1 binding sites were massively enriched in AAAA tetramers (A, adenosine) within 5–50 nucleotides (nt) downstream of the binding sites (Fig. 2c and Additional file 1: Figure S5A). The AAAA enrichment reflected the presence of A-rich stretches, which ranged from 8 to 30 nt in length (Additional file 1: Figure S5B; see the “Materials and methods” section). Within 3′ UTRs, 30% (1848 out of 6165) of MKRN1 binding sites resided immediately upstream of an A-rich stretch (Fig. 2a, d) and longer A-rich stretches associated with stronger MKRN1 binding (Additional file 1: Figure S5C,D). Intriguingly, we detected a requirement for a run of at least 8 continuous A’s to confer strong MKRN1 binding (Fig. 2e), which precisely matched the RNA footprint of one RNA recognition motif (RRM) domain of PABP [29]. Since PABPC1 was previously reported to bind not only at poly(A) tails but also within 3′ UTRs [30–32], these observations suggested that MKRN1 binds together with PABPC1 to mRNAs.

**Fig. 3:**
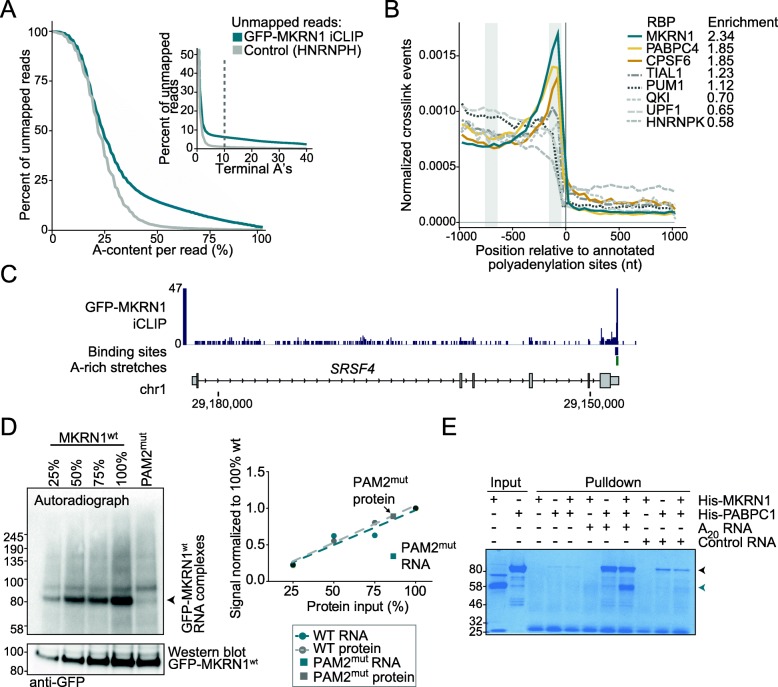
MKRN1 binds at poly(A) tails. **a** Unmapped MKRN1 iCLIP reads display increased A-content (more than half of all nucleotides in the read), evidencing poly(A) tail binding. Cumulative fraction of iCLIP reads (*y*-axis, merged replicates) that could not be mapped to the human genome (see the “Materials and methods” section) and show at least a given A-content (*x*-axis). iCLIP data for the unrelated RBP HNRNPH [33] are shown for comparison. Cumulative percentage of reads with a minimum number of terminal A’s is displayed as an inset. **b** MKRN1 crosslink events increase towards 3′ UTR ends. Metaprofile of MKRN1 crosslink events and seven additional RBPs shows the normalized sum of crosslink events per nt in a 2001-nt window around annotated polyadenylation sites of transcripts with > 1 kb 3′ UTRs. Gray bars indicate windows in 3′ UTR body (− 750 to − 650) and close to the poly(A) site (− 150 to − 50), which were used to calculate enrichment factors. **c** MKRN1 binds near the polyadenylation site of the *SRSF4* gene. Genome browser view as in Fig. 2a. **d** Overall RNA binding of MKRN1 is strongly reduced when abrogating PABPC1 interaction. Autoradiograph (left) of UV crosslinking experiments (replicate 1, with 4SU and UV crosslinking at 365 nm; replicates 2 and 3 in Additional file 1: Figure S6A,B) comparing GFP-MKRN1^PAM2mut^ with GFP-MKRN1^wt^ at different dilution steps for calibration. Quantification of radioactive signal of protein-RNA complexes and corresponding Western blot shown on the right. Uncropped gel images are shown in Additional file 3: Figure S11A,B. **e** MKRN1 is recruited to poly(A) RNA with the help of PABPC1. SDS-PAGE (Coomassie staining) shows recovery of recombinant His-MKRN1^wt^ and/or His-PABPC1 (marked by petrol and gray arrowheads, respectively) from pulldown of biotinylated RNA oligonucleotides, containing the last 22 nt of the *SRSF4* 3′ UTR followed by 20 A (A_20_ RNA) or 20 V nucleotides (A or C or G; control RNA). Beads without RNA served as controls. Replicates and uncropped gel images are shown in Additional file 1: Figure S6C,D and Additional file 3: S11C-E, respectively

Prompted by this notion, we examined the unusually high fraction of unmapped iCLIP reads in the MKRN1 dataset for untemplated trailing A’s to test the hypothesis that MKRN1 may also bind at poly(A) tails (Additional file 1: Table S2). The unmapped reads generally displayed an increased A-content (Fig. 3a), compared to an unrelated control RBP [33], and 6% of the reads ended in at least ten terminal A’s (Fig. 3a, inset). In addition, the mapped GFP-MKRN1^wt^ crosslink events were enriched upstream of annotated polyadenylation sites, as exemplified in the *SRSF4* gene (Fig. 3b, c). We compared this binding pattern to other RBPs using publicly available eCLIP data from the ENCODE project [34]. The binding of TIAL1, PUM1, QKI, UPF1, and HNRNPK, which are known to fulfill different functions in the 3′ UTR [35–38], was distributed throughout 3′ UTR bodies (Fig. 3b). In contrast, PABPC4 as well as CPSF6, a component of the cleavage and polyadenylation machinery, peaked together with MKRN1 towards the polyadenylation sites. Together, these results support that MKRN1, but not other 3′ UTR-binding proteins, binds at poly(A) tails, where it coincides with the poly(A)-binding protein.

In order to test whether the interaction with PABPC1 influences MKRN1 RNA binding, we performed UV crosslinking experiments with GFP-MKRN1^PAM2mut^, which no longer interacts with PABPC1 (Fig. 1d, e). Strikingly, RNA binding of this mutant was globally reduced compared to GFP-MKRN1^wt^ (Fig. 3d, Additional file 1: Figure S6A,B, and Additional file 3: Figure S11), indicating that PABPC1 might recruit MKRN1 to RNA. In order to substantiate this finding, we performed in vitro RNA pulldown assays with recombinant His-PABPC1 and/or His-MKRN1 and biotinylated RNAs. Notably, addition of His-PABPC1 strongly increased the pulldown of His-MKRN1 with the A_20_-harboring RNA but not the control RNA (Fig. 3e and Additional file 1: Figure S6C,D), indicating that PABPC1 stabilizes MKRN1 at poly(A) sequences.

In summary, these results strongly suggest that MKRN1 binds upstream of A-rich stretches in 3′ UTRs and poly(A) tails. We hypothesize that this binding pattern is defined via the interaction of MKRN1 with PABPC1, since (i) MKRN1 binding intensifies when the associated A-rich stretch reaches sufficient length to accommodate one RRM domain of PABPC1, (ii) abolishing the MKRN1-PABPC1 interaction results in loss of MKRN1 RNA binding, and (iii) PABPC1 reinforces MKRN1’s association with RNA in vitro. In a concordant scenario, it was found that *Drosophila* Mkrn1 binds before an extended A-rich stretch in the 3′ UTR of *oskar* mRNA and that this binding is significantly reduced upon depletion of pAbp (Dold et al., bioRxiv, 10.1101/501643).

### MKRN1 promotes ribosome stalling at poly(A) sequences

**Fig. 4:**
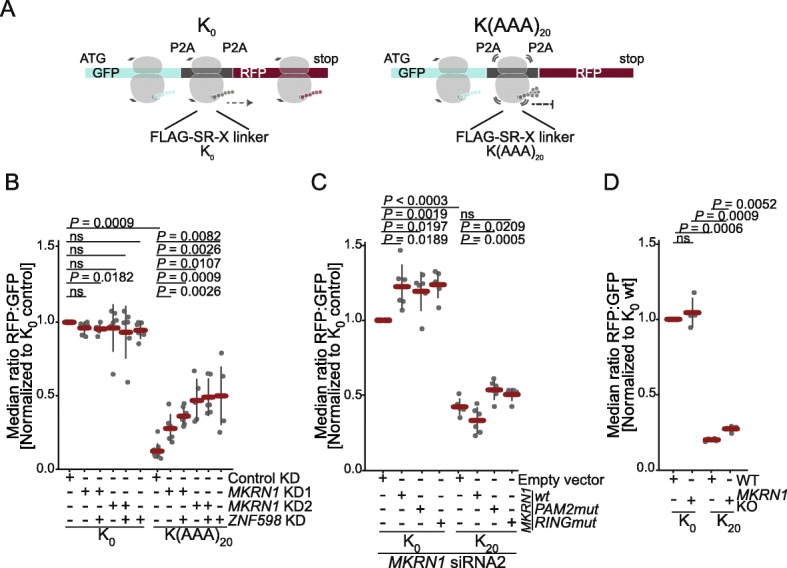
MKRN1 stalls ribosomes at poly(A) sequences. **a** The dual fluorescence reporter harbors an N-terminal GFP, followed by a FLAG-SR-X linker and a C-terminal RFP, which are separated by P2A sites to ensure translation into three separate proteins [8]. The resulting GFP:RFP ratio was determined using flow cytometry. The inserted fragment K(AAA)_20_ encodes 20 lysines by repeating the codon AAA. The starting vector without insert (K_0_) served as control. Schematic ribosomes illustrate translation of the respective reporter segments. **b** Ribosomes fail to stall in the absence of MKRN1. HEK293T cells were transfected with control siRNA or siRNAs targeting *MKRN1* (KD1 and KD2) or *ZNF598* for 24 h, followed by transfection of the reporter plasmids for 48 h. Western blots for KDs are shown in Additional file 1: Figure S8A. RFP and GFP signals were analyzed by flow cytometry. Median RFP:GFP ratios, normalized to K_0_ in control, are shown. Error bars represent s.d.m.; *P* values indicated above (paired two-tailed Student’s *t* test, Benjamini-Hochberg correction, *n* ≥ 6 replicates; ns, not significant). Analyses for inserts coding for 12 lysines (K(AAA)_12_) and ten arginines (R(CGA)_10_) in the dual fluorescence reporter are shown in Additional file 1: Figure S7A. **c** Expression of MKRN1^wt^ can rescue ribosome stalling. HEK293T cell lines with stable integrations of siRNA2-insensitive MKRN1 wild type and mutant constructs, or empty vector, were transfected with *MKRN1* siRNA2 for 24 h, followed by transfection of the reporter plasmids for 48 h. RFP and GFP signals were analyzed by flow cytometry. Median RFP:GFP ratios, normalized to K_0_ in WT cells, are shown. Error bars represent s.d.m.; *P* values indicated above (paired two-tailed Student’s *t* test, Benjamini-Hochberg correction, *n* = 6 replicates; ns, not significant). Analyses for reporter plasmids with inserts coding for K(AAA)_12_ or R(CGA)_10_ are shown in Additional file 1: Figure S7B. **d** *MKRN1* knockout (*MKRN1* KO) and wild type (WT) HEK293T cells were transfected with the reporter plasmids for 48 h. Measurements, analyses, and visualization as in **c** (*n* = 4 replicates). Analyses for reporter plasmids with inserts coding for K(AAA)_12_ or R(CGA)_10_ are shown in Additional file 1: Figure S7C (**d**)

As outlined above, our iCLIP data demonstrated that MKRN1 marks the beginning of poly(A) tails. Hence, it is conceivable that MKRN1 will also bind upstream of premature polyadenylation events within open reading frames. Based on MKRN1’s binding pattern, its interaction partners, and its association with ribosomes, we hypothesized that MKRN1 may be involved in the clearance of such transcripts by ribosome-associated quality control (RQC). In this process, ribosomes that translate into a poly(A) sequence, for instance upon stop codon readthrough or premature polyadenylation, are stalled and eventually recycled [4, 5]. To test this hypothesis, we employed a recently introduced flow cytometry-based assay that monitors ribosome stalling in a dual fluorescence reporter [8, 10]. In this reporter, the genes encoding the green and red fluorescent protein (GFP and RFP, respectively) are separated by a linker region with a poly(A) stretch as putative ribosome stalling sequence (Fig. 4a). The linker is flanked by two viral P2A sites that promote translational elongation into separate protein products, thereby disconnecting the translation of the linker region from the flanking GFP and RFP translation products [39]. As a consequence, complete translation of the reporter results in equal amounts of three stand-alone proteins (GFP, linker peptide, and RFP), whereas abortion of translation within the linker sequence impairs RFP, but not GFP production. This results in a reduced RFP:GFP ratio [8], which is measured using fluorescence-based flow cytometry.

As reported previously, inserting a K(AAA)_20_ linker (encoding 20 lysine residues) into the reporter resulted in predominant ribosome stalling compared to the starting vector (K_0_, Fig. 4b and Additional file 1: Figure S7). Importantly, *MKRN1* depletion with two independent siRNAs led to a reproducible recovery of RFP expression downstream of K(AAA)_20_, suggesting that many ribosomes failed to stall at K(AAA)_20_ (*MKRN1* KD1 and KD2; Fig. 4b, Additional file 1: Figure S7A, S8A,B, and Additional file 3: Figure S12). *MKRN1* KD2 seemed slightly more effective, possibly because this siRNA simultaneously decreased the transcript levels of the close paralog *MKRN2* (Additional file 1: Figure S8B). The specific impact of MKRN1 on ribosome stalling was further supported by complementing the *MKRN1* KD with stable integration of *MKRN1* constructs. Only MKRN1^wt^ was capable of partially reverting the KD effect, such that ribosome stalling was partially restored, whereas neither of the MKRN1 mutants was functional in this assay (Fig. 4c and Additional file 1: Figure S7B). In addition, we found that *MKRN1* KD2 did not influence the abundance of the underlying reporter RNA (Additional file 1: Figure S7D), indicating that RQC for this reporter is not coupled to appreciable mRNA destabilization in HEK293T cells, in line with a previous study [8].

In order to relate MKRN1’s effect to other players of the RQC pathway, we knocked down *ZNF598*, the E3 ubiquitin ligase that was recently reported to stall ribosomes during RQC [8–10]. Importantly, *MKRN1* KD2 impaired ribosome stalling to a similar extent as KD of *ZNF598*. Simultaneous depletion of *MKRN1* and *ZNF598* was not additive, indicating that both proteins work in the same pathway (Fig. 4b and Additional file 1: Figure S7A). We could not detect an interaction between MKRN1 and ZNF598 in pulldown experiments (Fig. 1d). However, we noted a certain level of cross-regulation, such that *ZNF598* expression was decreased in *MKRN1* KD1 (but not in *MKRN1* KD2), whereas *ZNF598* overexpression reduced *MKRN1* expression (Additional file 1: Figure S8). Overall, these results suggest that MKRN1 fulfills a specific function in RQC complementary to ZNF598.

For independent validation of the KD results, we generated a stable *MKRN1* knockout (KO) cell line using CRISPR/Cas9 genome editing (Additional file 1: Figure S8C,D). Reporter assays showed that complete loss of *MKRN1* impaired ribosome stalling at K(AAA)_20_, albeit with a smaller effect size compared to the siRNA-mediated KD (Fig. 4d and Additional file 1: Figure S7C). qPCR indicated a compensatory upregulation of the paralog *MKRN2* in the *MKRN1* KO but not in the *MKRN1* KD (Additional file 1: Figure S8B,D), which could explain the reduced effect size of the *MKRN1* KO in the reporter assays. In line with a partially redundant role of MKRN2, we find that simultaneous depletion of *MKRN1* and *MKRN2*, as observed upon KD with siRNA2 (Additional file 1: Figure S8B), shows a larger effect than KD with siRNA1, which does not change *MKRN2* levels.

Based on these results, we propose MKRN1 as a novel player in RQC that contributes to efficient ribosome stalling at poly(A) sequences. Our experiments suggest that this function likely depends on the interaction of MKRN1 with PABPC1 as well as its E3 ubiquitin ligase activity.

### MKRN1 mediates the ubiquitylation of RPS10 and PABPC1

**Fig. 5:**
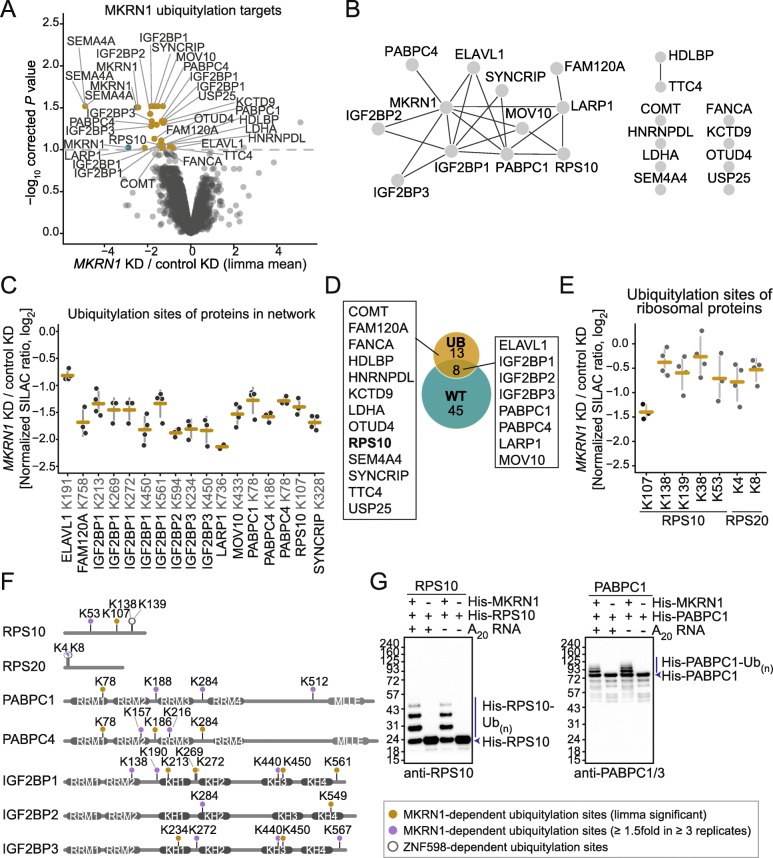
MKRN1 ubiquitylates ribosomal protein RPS10 and translational regulators. **a** Ubiquitin remnant profiling to compare the relative abundance of ubiquitylation sites in *MKRN1* KD2 and control HEK293T cells. Ubiquitin remnant peptides were enriched and analyzed by quantitative mass spectrometry, quantifying a total of 15,528 ubiquitylation sites on 4790 proteins. 29 putative MKRN1 target sites with significantly decreased ubiquitylation upon *MKRN1* KD2 (FDR < 10%, *n* = 4 replicates) are highlighted and labeled with the respective protein name. Note that many proteins contain several differentially regulated ubiquitylation sites. **b** Protein interaction network of 21 proteins with putative MKRN1 ubiquitylation target sites (significantly reduced, shown in **a**). The functional interactions were obtained from the STRING and BioGrid databases and our study. Visualization by Cytoscape. **c** Ubiquitin remnant profiling results for significantly regulated ubiquitylation sites (FDR < 10%) in proteins from network in **b**. Mean and standard deviation of the mean (s.d.m., error bars) are given together with all data points. **d** Comparison of interactome of GFP-MKRN1^wt^ (WT over GFP, see Fig. 1a) with putative MKRN1 ubiquitylation substrates from ubiquitin remnant profiling (UB, see **a**). Protein names are given for all ubiquitylation substrates. **e** Ubiquitin remnant profiling results for seven quantified ubiquitylation sites in RPS10 and RPS20. Significant changes are shown in black (FDR < 10%) and non-significant changes in gray. Representation as in **c**. **f** Comparison of ubiquitylation sites in the target proteins RPS10 (UniProt ID P46783), RPS20 (P60866), PABPC1 (P11940), PABPC4 (B1ANRO), IGF2BP1 (Q9NZI8), IGF2BP2 (F8W930), and IGF2BP3 (O00425) that are modified by ZNF598 and MKRN1 during RQC. **g** MKRN1 ubiquitylates RPS10 and PABPC1 in vitro. His-RPS10 (left) or His-PABPC1 (right) were incubated with or without His-MKRN1. Ubiquitylation of the target proteins was assessed by Western blot. Replicates 2, 3, and uncropped gel images are shown in Additional file 3: Figure S12I,J for RPS10 and Additional file 3: Figure S12K,L for PABPC1

RQC builds on a series of ubiquitylation events by multiple E3 ubiquitin ligases, including Listerin and ZNF598 [5]. In order to identify putative ubiquitylation substrates of MKRN1, we performed ubiquitin remnant profiling to compare the relative abundance of di-glycine-modified lysines in *MKRN1* KD and control cells. We quantified 2324 ubiquitylation sites (in 1264 proteins) that were detected in all four replicate experiments (Additional file 4: Table S3). Notably, *MKRN1* depletion led to a significantly decreased abundance of 29 ubiquitylation sites on 21 proteins (FDR < 10%, Fig. 5a). Comparing the relative abundance of putative substrates in total extracts showed that none of them significantly changed in the *MKRN1* KD cells, suggesting that ubiquitylation does not trigger substantial degradation of these proteins (Additional file 1: Figure S9A and Additional file 5: Table S4). This result was further confirmed by Western blot for several proteins that were identified as putative MKRN1 substrates (Additional file 1: Figure S9B).

The majority of the ubiquitylation targets formed a functional cluster of translational regulators based on our own interactome data, previously reported protein-protein interactions, and functional annotations (Fig. 5b and Additional file 1: Figure S9C). Intriguingly, we identified a MKRN1-dependent ubiquitylation site on RPS10, a 40S ribosomal protein that was previously reported to be modified by ZNF598 during RQC [8–10]. Here, *MKRN1* KD led to a significant decrease in ubiquitylation at lysine 107 of RPS10 (K107; Fig. 5c–f). In addition, *MKRN1 KD* decreased ubiquitylation of a number of interaction partners including PABPC1/4, IGF2BP1/2/3, LARP1, MOV10, and ELAVL1 (Fig. 5d). In order to test whether MKRN1 can directly modify RPS10 and PABPC1, we performed in vitro ubiquitylation assays with recombinant proteins. Western blot experiments detected the appearance of multiple mono- and poly-ubiquitylated variants of RPS10 and PABPC1, illustrating that MKRN1 efficiently ubiquitylated both proteins (Fig. 5g). We therefore propose that rather than recruiting ZNF598 or another E3 ubiquitin ligase, MKRN1 plays an active role in RQC by regulatory ubiquitylation of RPS10.

## Discussion

**Fig. 6:**
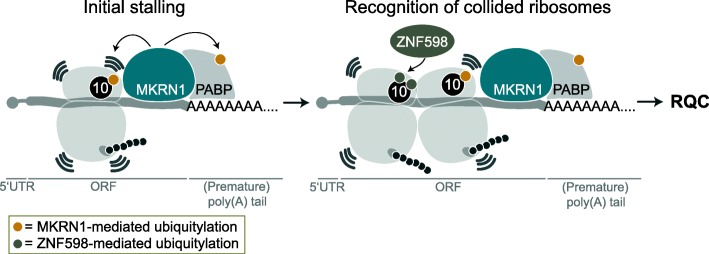
MKRN1 is a sensor for poly(A) sequences that stalls ribosomes to initiate ribosome-associated quality control. Proposed model of MKRN1 function: MKRN1 is positioned upstream of (premature) poly(A) tails via interaction with PABPC1. Ribosomes translating the open reading frame run into MKRN1 that acts as a roadblock to prohibit poly(A) translation. Upon contact with the translating ribosome, MKRN1 ubiquitylates the 40S ribosomal protein RPS10. This stalls the ribosome, causing the trailing ribosomes to collide. ZNF598 recognizes the collided ribosomes and ubiquitylates ribosomal proteins to promote RQC

Ribosome-associated quality control is essential to recognize and clear terminally stalled ribosomes. Here, we put forward MKRN1 as a novel factor in RQC. Our data suggest that MKRN1 is positioned upstream of poly(A) sequences through direct interaction with PABPC1, thereby marking the beginning of poly(A) tails. We propose that in case of premature polyadenylation or stop codon read-through, MKRN1 stalls the translating ribosome and initiates RQC by ubiquitylating ribosomal protein RPS10, PABPC1, and other translational regulators (Fig. 6).

Central to our model is the specific RNA binding behavior of MKRN1, which is recruited to mRNA by PABPC1 to mark the beginning of A-rich stretches and poly(A) tails. This hypothesis builds on the strong interaction of both proteins, the reduced RNA binding of MKRN1 when the interaction is abolished in vivo, the reinforced RNA binding of MKRN1 in the presence of PABPC1 in vitro, and the occurrence of strong MKRN1 binding when at least 8 continuous A’s are present. The latter mirrors the footprint of one RRM domain of PABPC1, indicating that the binding of one RRM of PABPC1 to poly(A) is sufficient for MKRN1 recruitment [29]. Of note, a study with the Mkrn1 ortholog from *D. melanogaster* demonstrates binding of a Mkrn1/pAbp complex at an A-rich stretch in the 3′ UTR of *oskar* mRNA, which is involved in translational control and required for oogenesis (Dold et al., bioRxiv, doi: 10.1101/501643).

Our data show that MKRN1 associates with polysomes and ubiquitylates RPS10, indicating a role in translational control. We hypothesize that ribosomes encountering the MKRN1-PABPC1 complex are stalled, possibly via ubiquitylation of RPS10 on K107 and other MKRN1 substrates. Concordantly, ZNF598 was also found to mediate ubiquitylation of RPS10 on K138/K139 [11]. In conjunction with the unique RNA binding behavior, we therefore hypothesize that MKRN1 acts as a first line of defense against poly(A) translation. We propose that MKRN1 is recruited by PABPC1 to the beginning of poly(A) tails, including premature polyadenylation events within open reading frames, where it represents a physical “roadblock” to the translating ribosome. Upon contact with the translating ribosome, MKRN1 ubiquitylates K107 on RPS10, thereby stalling the ribosome before it translates the poly(A) tail. Subsequently, the trailing ribosomes collide with the initially stalled ribosome. ZNF598 recognizes the collision interface and ubiquitylates the collided ribosomes [11, 40]. In summary, we suggest that a sequence of MKRN1-mediated and ZNF598-mediated ubiquitylation events on ribosomal proteins and possibly other factors, including PABPC1, triggers ribosome-associated quality control. The type of ubiquitin chain deposited by MKRN1 on RPS10 as well as the possible existence of deubiquitylating enzymes that cleave the ubiquitin chain from RPS10 remain to be investigated.

Many known components of the RQC machinery, such as Listerin (Ltn1p in yeast) and ZNF598 (Hel2p in yeast), are conserved from yeast to human; however, the molecular signals that are recognized partially differ. In yeast, RQC can be triggered by an excess of positively charged amino acids (lysine and arginine), which are sensed while they pass through the ribosomal exit tunnel [41, 42]. In contrast, in human, sensing the aberrant mRNAs does not occur via the encoded amino acids but at the level of the mRNA sequence and corresponding tRNAs, such that only poly(A) effectively results in ribosome stalling [8, 9, 43]. We propose that MKRN1 acts as a direct reader of poly(A) sequences based on its interaction with PABPC1. Consistent with this conceptual difference, there is no functionally equivalent ortholog of MKRN1 in yeast (Yth1p and Lee1p are similar, but lack the RING domain and the PAM2 motif; Additional file 1: Figure S1C). However, a recent study in yeast has shown that Hel2p binds before and also after the stop codon within 3′ UTRs of mRNAs [44], while such a binding pattern has not been observed for ZNF598 in human cells [9]. Even though MKRN1 and Hel2p binding patterns are not identical, this suggests the possibility that the roles of yeast Hel2p might have been split between MKRN1 and ZNF598 in human cells.

Why yeast and human employ partially different mechanisms to detect poly(A) translation is currently unclear, but it has been suggested that spurious translation of poly-lysine stretches from long human poly(A) tails might target the aberrant proteins to the nucleus [8]. Loss of mRNA surveillance and RQC deficiency can lead to protein aggregation and culminate in proteotoxic stress, which in turn is lined to neurological disorders such as amyotrophic lateral sclerosis [45, 46]. Hence, recognition of poly(A) sequences prior to their translation, possibly by the action of MKRN1, might be particularly beneficial in humans.

## Materials and methods

### Cell culture

HEK293T cells were obtained from DSMZ and cultured in DMEM (Life Technologies) with 10% fetal bovine serum (Life Technologies), 1% penicillin/streptomycin (Life Technologies), and 1% l-glutamine (Life Technologies). All cells were maintained at 37 °C in a humidified incubator containing 5% CO_2_ and routinely tested for mycoplasma infection. For SILAC labeling, cells were maintained in media containing either l-arginine and l-lysine (light SILAC label), l-arginine (^13^C_6_) and l-lysine (^2^H_4_) (medium SILAC label), or l-arginine (^13^C_6_-^15^N_4_) and l-lysine (^13^C_6_-^15^N_2_) (heavy SILAC label) (Cambridge Isotope Laboratories).

### Vectors

The following vectors, suitable for Gateway Cloning, were obtained either from the IMB Core Facility ORFeome Collection [47] or from the Harvard PlasmID Repository (https://plasmid.med.harvard.edu/PLASMID/): pENTR221-MKRN1, pENTR221-PABPC1, pENTR223.1-IGF2BP1, pENTR221-ELAVL1, and pCMV-SPORT-ZNF598. Coding sequences from the entry vectors were cloned into the mammalian expression vectors pMX-DEST53-IP-GFP by LR Gateway cloning according to the manufacturer’s recommendations (Gateway LR Clonase II Enzyme mix; Life Technologies). Dual fluorescence reporter plasmids (pmGFP-P2A-K_0_-P2A-RFP, pmGFP-P2A-(K^AAA^)_12_-P2A-RFP, pmGFP-P2A-(K^AAA^)_20_-P2A-RFP, and pmGFP-P2A-(R^CGA^)_10_-P2A-RFP) were generously provided by Ramanujan S. Hegde (MRC Laboratory of Molecular Biology, Cambridge, UK) [8].

### Cloning

All MKRN1 mutant plasmids were generated with the Q5 Site-Directed Mutagenesis Kit (NEB) according to the manufacturer’s recommendations. In order to disrupt MKRN1’s interaction with PABPC1 (MKRN1^PAM2mut^), three-point mutations were introduced into the PAM2 motif (A169S, F172A, P174A; Fig. 1c) as previously described [25]. In MKRN1^RINGmut^, a previously described mutation in the RING domain (H307E) was introduced to abolish E3 ubiquitin ligase function [14]. In order to obtain a siRNA2-insensitive copy of *MKRN1* for complementation, we used alternative codons and wobble positions where possible to synonymize the nucleotide sequence in the siRNA target site. For untagged MKRN1, GFP was deleted from the pMX-DEST-GFP vector using the Q5 Site-Directed Mutagenesis Kit. All oligonucleotides used for cloning are listed in Additional file 1: Table S5.

### Transfections

Overexpression of vectors was performed using Polyethylenimine MAX 4000 (PEI, Polysciences, 24885-2) with a DNA:PEI ratio of 1:10. Knockdowns were performed with siRNAs (Additional file 1: Table S6) using Lipofectamine RNAiMAX (Life Technologies) according to the manufacturer’s recommendations.

### Affinity purification (AP) for Western blot analyses

GFP-based affinity purifications (APs) were performed as described before [21]. In brief, HEK293T cells transiently expressing GFP (empty vector) or a GFP-tagged target protein were used. The cells were lysed in modified RIPA (mRIPA) buffer supplemented with protease inhibitors (protease inhibitor cocktail, Sigma), 1 mM sodium orthovanadate, 5 mM β-glycerophosphate, 5 mM sodium fluoride, and 10 mM *N*-ethylmaleimide (NEM) (all from Sigma). Protein concentrations were determined using the Pierce BCA Protein Assay Kit (Thermo Fisher). GFP-trap agarose beads (Chromotek) were incubated with the cleared lysate for 1 h at 4 °C. After five washes with mRIPA buffer, the beads were resuspended in LDS sample buffer (Life Technologies) and heated to 70 °C for 10 min. For RNase digests, the enriched proteins were incubated with 0.5 U/μl RNase A (Qiagen) and 20 U/μl RNase T1 (Thermo Fisher Scientific) for 30 min at 4 °C after the first two washes in mRIPA buffer.

### Sample preparation for the protein interactome analysis

GFP-based APs were performed as described before [21]. In brief, HEK293T cells transiently expressing GFP (empty vector) were cultured in light SILAC medium, while cells expressing N-terminally GFP-tagged MKRN1 wt or mutants were cultured in medium or heavy SILAC medium. The cells were lysed as described above. After washing in mRIPA buffer, GFP-trap agarose beads were incubated with the cleared lysate for 1 h at 4 °C. All AP samples were washed four times with mRIPA buffer, combined and washed again in mRIPA buffer. The beads were heated in LDS sample buffer, supplemented with 1 mM dithiothreitol (DTT; Sigma, D5545) for 10 min at 70 °C and alkylated using 5.5 mM 2-chloroacetamide (CAA; Sigma, C0267) for 30 min at RT in the dark [48].

### Sample preparation for the proteome analysis

*MKRN1* KD using siRNA2 was performed in heavy labeled SILAC cells, and control KD was performed in light labeled SILAC cells in two replicates. For the third replicate, a label swop was performed, knocking down *MKRN1* (siRNA2) in light labeled SILAC cells and control in heavy labeled SILAC cells. For proteome analysis, cells were lysed as described above. Subsequently, 25 μg protein from each SILAC condition (50 μg in total) were pooled and processed as described below.

### Sample preparation for mass spectrometry

The enriched proteins were resolved by SDS-PAGE on a NuPAGE 4–12% Bis-Tris protein gel (Thermo Fisher Scientific) and stained using the Colloidal Blue Staining Kit (Life Technologies). Proteins were in-gel digested using trypsin, before peptides were extracted from the gel. To concentrate, clear, and acidify the peptides, they were bound to C18 StageTips as described previously [49].

### Mass spectrometry data acquisition

Peptide fractions were analyzed on a quadrupole Orbitrap mass spectrometer (Thermo Q Exactive Plus, Thermo Scientific) coupled to an uHPLC system (EASY-nLC 1000, Thermo Scientific) [50]. Peptide samples were separated on a C18 reversed phase column (length 20 cm, inner diameter 75 μm, bead size 1.9 μm) and eluted in a linear gradient from 8 to 40% acetonitrile containing 0.1% formic acid in 105 min for the interactome analyses, in 175 min for the proteome analyses, or in 125 min for the ubiquitylome analyses. The mass spectrometer was operated in data-dependent positive mode, automatically switching between MS and MS^2^ acquisition. The full scan MS spectra (m/z 300–1650) were acquired in the Orbitrap. Sequential isolation and fragmentation of the ten most abundant ions were performed by higher-energy collisional dissociation (HCD) [51]. Peptides with unassigned charge states, as well as with charge states less than + 2 were excluded from fragmentation. The Orbitrap mass analyzer was used for acquisition of fragment spectra.

### Peptide identification and quantification

Raw data files were analyzed and peptides were identified using the MaxQuant software (version 1.5.28) [52]. Parent ion and MS^2^ spectra were compared to a database containing 92,578 human protein sequences obtained from UniProtKB (release June 2018), coupled to the Andromeda search engine [53]. Cysteine carbamidomethylation was set as a fixed modification. N-terminal acetylation, oxidation, and *N*-ethylmaleimide (NEM) were set as variable modifications. For ubiquitylome data analysis, glycine-glycine (GlyGly) modification of lysine was additionally set as a variable modification. The mass tolerance for the spectra search was set to be lower than 6 ppm in MS and 20 ppm in HCD MS^2^ mode. Spectra were searched with strict trypsin specificity and allowing for up to three mis-cleavages. Site localization probabilities were determined by MaxQuant using the PTM scoring algorithm as described previously [54, 55]. Filtering of the dataset was based on the posterior error probability to arrive at a false discovery rate (FDR) < 1% estimated using a target-decoy approach. Proteins that were categorized as “only identified by site”, potential contaminants and reverse hits were removed. Only proteins identified with at least two peptides (including at least one unique peptide) and a SILAC ratio count of at least two were used for analysis. For AP experiments, proteins that were quantified in at least two out of three experiments were kept for further analysis. In total, we quantified 1106 and 1097 protein groups in the AP experiments with GFP-MKRN1^wt^ (Fig. 1a), GFP-MKRN1^PAM2mut^ (Fig. 1e) and GFP-MKRN1^RINGmut^ (Additional file 1: Figure S3), respectively (Additional file 2: Table S1). The SILAC ratios were log_2_-transformed and converted into an asymmetric *z*-score based on the mean and interquartile range of the distribution as described previously [54]. For statistical analysis, a moderated t-test from the limma algorithm was used [56]. Enriched proteins with an FDR < 5% were determined to be significantly enriched interactors (for GFP-MKRN1^wt^). For proteins enriched in GFP-MKRN1^RINGmut^ over GFP-MKRN1^wt^, proteins with an FDR < 5% and a GFP-MKRN1^wt^/GFP *z*-score > 1 were selected. In the proteome experiment, we quantified 6439 protein groups, present in all three replicates. Ratio-ratio and ratio-intensity plots were created in R (version 3.4.3) using RStudio (http://www.rstudio.com/).

### Functional annotation of target proteins

In order to assess the functions of MKRN1-interacting proteins and proteins with MKRN1-dependent ubiquitylation sites, we performed gene ontology (GO) enrichment analyses using the Database for Annotation, Visualization and Integrated Discovery (DAVID 6.7) for three GO domains [57]. Enriched GO terms (modified Fisher exact test, adjusted *P* value < 0.05, Benjamini-Hochberg correction; Additional file 1: Figure S2A and Figure S9C) were visualized using REVIGO (Reduce & Visualize Gene Ontology) allowing medium GO term similarity [58].

### Western blot

Denatured proteins were separated by SDS-PAGE on a NuPAGE 4–12% Bis-Tris protein gel (Life Technologies) and transferred to a 0.45-μm nitrocellulose membrane (VWR). For detection, either fluorophore-coupled secondary antibodies or HRP-conjugated secondary antibodies and WesternBright Chemiluminescent Substrate (Biozym Scientific) or SuperSignal West Pico Chemiluminescent Substrate (Life Technologies) were used. Western blots were quantified by determining the background-subtracted densities of the protein of interest using ImageJ [59]. The signal from the AP (against GFP-tagged protein of interest) was normalized to the respective control samples expressing the empty vector or to the input.

### Antibodies

The following antibodies were used: anti-GFP (B-2 clone; Santa Cruz; sc-9996), anti-MKRN1 (Bethyl Laboratories, A300-990A), anti-PABPC1/3 (Cell Signaling, 4992), anti-ZNF598 (N1 N3; GeneTex; GTX119245), anti-ELAVL1 (Santa Cruz, sc-5261), anti-LARP1 (Santa Cruz, sc-515873), anti-Ubiquitin (P4D1; Santa Cruz, sc-8017), anti-Vinculin (Sigma Aldrich, V9264), anti-αTubulin (Sigma Aldrich, T-5168), anti-Rabbit IgG (Cell Signaling; 7074), anti-Mouse IgG (Cell Signaling; 7076), IRDye® 680RD Goat anti-Mouse IgG (P/N 925-68070), and IRDye® 800CW Goat anti-Rabbit IgG (P/N 925-32211) (both LI-COR Biosciences GmbH).

### Polysomal fractionation

HEK293T cells were subjected to gradient centrifugation for polysomal fractionation. Briefly, 4 × 10^6^ cells were seeded in 15-cm cell culture dishes and incubated overnight at 37 °C in a humidified atmosphere with 5% CO_2_. To stall translation, 100 μg/ml cycloheximide (CHX) were added 10 min prior to harvest. Cells were washed with PBS/CHX (100 μg/ml), and lysed in 750 μl polysome lysis buffer (140 mM KCl, 20 mM Tris-HCl pH 8.0, 5 mM MgCl_2_, 0.5% NP40, 0.5 mg/ml heparin, 1 mM DTT, 100 U/ml RNasin [Promega, Mannheim, Germany], 100 μg/ml CHX). The cell debris was pelleted by centrifugation (5 min, 4 °C, 13,000 rpm), and 600 μl of the cleared cell lysates were layered onto 11 ml 10–50% continuous sucrose gradients. The sucrose gradients were subjected to ultra-centrifugation at 35,000 rpm for 2 h at 4 °C without break using an SW40 Ti rotor (Beckman Coulter, Brea, USA). Afterwards, 1-ml fractions were collected using a Gradient Station (BioComp Instruments, Fredericton, Canada). UV-absorbance was measured at 254 nm. Protein was precipitated by adding trichloro acetic acid (TCA) to each fraction (final 10% [v/v]) and incubation overnight at 4 °C. The samples were centrifuged for 20 min at 4 °C and 13,000 rpm and the resulting pellets were washed twice with ice-cold acetone, dissolved in 100 μl 2× SDS loading buffer (62.5 mM Tris-HCl pH 6.8, 2.5 mM DTT, 10% glycerol [v/v], 1% SDS [w/v], 0.001% bromophenol blue) and heated for 5 min at 95 °C.

### Recombinant protein expression

N-His_6_-tagged hsRPS10, hsPABPC1, and hsMKRN1 variants were expressed from pET53-DEST in *E. coli* Rosetta™ 2(DE3) pLysS (Novagen). Cells were grown in LB-Luria at 37 °C and 160 rpm. For all MKRN1 variants, 50 μM ZnCl_2_ was added to the growth medium. For the expression of His-RPS10 and His-MKRN1 variants, cells were chilled on ice at OD_600_ of 0.6–0.8 and expression was induced by addition of IPTG (0.5 mM final concentration). Cells were further incubated at 18 °C and 160 rpm for 16–21 h. Cells expressing His-PABPC1 were grown at 37 °C and 160 rpm for 4 h.

Cells were harvested by centrifugation (4000×*g*, 15 min, 4 °C) and lysed in 38 ml of lysis buffer (50 mM Tris-HCl pH 8.0, 500 mM NaCl, 15 mM imidazole, 1 mM DTT, 1 mM MgCl_2_, Benzonase 1:5000 [Sigma]) per liter of initial culture volume using a high pressure homogenizer (Constant Systems, TS-T240). Lysates were cleared by centrifugation (45,000×*g*, 30 min, 4 °C). All proteins, except His-MKRN1-RING^mut^, were purified as follows: Proteins were passed over a HisTrap FF 5 ml column (GE Healthcare). After extensive washing with wash buffer (50 mM Tris-HCl pH 8.0, 500 mM NaCl, 15 mM imidazole), His-tagged proteins were eluted using wash buffer containing 300 mM imidazole. Elution fractions were pooled and diluted 1:10 in heparin-binding buffer (17 mM NaP_i_ pH 7.4, 50 mM NaCl, 5% glycerol, 1 mM DTT), or in the case of His-MKRN1-WT and His-MKRN1-PAM2^mut^, in anion exchange binding buffer (20 mM Tris-HCl pH 8.0, 30 mM NaCl, 5% glycerol, 1 mM DTT). The diluted proteins were subsequently passed over a HiTrap Heparin HP 5 ml column (GE Healthcare; His-RPS10 and His-PABPC1), or a HiTrap Q HP 5 ml column (GE Healthcare; His-MKRN1 variants). After washing with the respective binding buffer, recombinant proteins were eluted by running a linear gradient of 0–1.5 M NaCl in binding buffer over 20 column volumes. Fractions containing the respective recombinant protein were pooled and concentrated using Amicon® Ultra-15 spin concentrators (Merck Millipore). Concentrated protein pools were run on a Superdex 200 Increase 10/300 GL, or HiLoad 16/60 Superdex 200 prep grade column (GE Healthcare) in gel filtration buffer (40 mM Na-HEPES pH 7.4, 100 mM NaCl, 1 mM MgCl_2_, 1 mM DTT, 10% glycerol). Peak fractions containing the recombinant protein were pooled, aliquoted, and snap-frozen in liquid nitrogen. Frozen aliquots were stored at − 80 °C. All purification steps were performed using a Biorad NGC Quest Plus FPLC system. As His-MKRN1-RING^mut^ was prone to precipitation, it was isolated by a rapid one-step pulldown using Ni-NTA agarose beads (binding buffer: 20 mM Tris-HCl pH 7.5, 500 mM NaCl, 30 mM imidazole; elution buffer: 20 mM Tris-HCl pH 7.5, 500 mM NaCl, 1 mM MgCl_2_, 500 mM imidazole). 1 mM DTT and 10% glycerol were added to the eluted His-MKRN1-RING^mut^, and it was snap-frozen in liquid nitrogen.

### In vitro ubiquitylation assays

Autoubiquitylation assay: To assess the functionality and capability of autoubiquitylation of recombinant MKRN1, His-MKRN1-WT (1 μM), His-MKRN1-PAM2^mut^ (1.5 μM), or His-MKRN1-RING^mut^ (1.5 μM) were incubated with or without E2 enzyme UBC5a (2 μM), with E1 enzyme UBA1 (0.25 μM) (IMB Protein Production Core Facility), ATP (30 μM), ubiquitin (5 μM; Sigma), and DTT (1 mM) in 1× MAB reaction buffer (10× MAB reaction buffer: 400 mM HEPES pH 7.4, 500 mM NaCl, 80 mM MgCl_2_, 1 mM DTT) for 1 h at 37 °C. As a control, the E2 enzyme Ubc5a was incubated with all reaction components without any E3 enzyme. The reaction was stopped by adding LDS sample buffer and boiling.

Ubiquitylation of target proteins: To assess whether recombinant MKRN1 is capable of ubiquitylating RPS10 and PABPC1, His-MKRN1-WT (1 μM) was incubated with His-PABPC1 (0.75 μM) or His-RPS10 (0.75 μM), the E2 enzyme UBC5a (2 μM), the E1 enzyme UBA1 (0.25 μM), ATP (30 μM), ubiquitin (5 μM), and DTT (1 mM) in 1× MAB reaction for 1 h at 37 °C. As a control, His-PABPC1 or His-RPS10 were incubated with all reaction components without His-MKRN1-WT. The reaction was stopped by adding LDS sample buffer and boiling.

### RNA isolation, cDNA synthesis, and qPCR

Cells were washed twice in ice-cold PBS and harvested. RNA was isolated using the RNeasy Plus Mini Kit (Qiagen) according to the manufacturer’s recommendations. Five hundred nanograms of total RNA was transcribed into cDNA using random hexamer primers (Thermo Scientific) and the RevertAid Reverse Transcriptase (Thermo Scientific) according to the manufacturer’s recommendations. qPCR was performed using the Luminaris HiGreen qPCR Master Mix, low ROX (Thermo Scientific) according to the manufacturer’s recommendations with 10 μM forward and reverse primers (Additional file 1: Table S5).

### iCLIP experiments and data processing

iCLIP libraries were prepared as described previously [60, 61]. HEK293T cells ectopically expressing either GFP alone (empty vector) or N-terminally GFP-tagged MKRN1 wild type (GFP-MKRN1^wt^), GFP-MKRN1^PAM2mut^, or GFP-MKRN1^RINGmut^ were used. For crosslinking, confluent cells were irradiated once with 150 mJ/cm^2^ at 254 nm in a Stratalinker 2400 or treated with 4-thiouridine (100 μM for 16 h) and irradiated with 3 × 300 mJ/cm^2^ in a Stratalinker 2400 with 365 nm bulbs. For IP, 10.5 μg anti-GFP antibody (goat, Protein Unit, MPI-CBG, Dresden) was used per sample. The libraries were sequenced as 50-nt single-end reads on an Illumina MiSeq platform (Additional file 1: Table S2).

Basic sequencing quality checks were applied to all reads using FastQC (version 0.11.5) (https://www.bioinformatics.babraham.ac.uk/projects/fastqc/). Afterwards, reads were filtered based on sequencing qualities (Phred score) of the barcode region. Only reads with at most one position with a sequencing quality < 20 in the experimental barcode (positions 4 to 7) and without any position with a sequencing quality < 17 in the random barcode (positions 1–3 and 8–9) were kept for further analysis. Remaining reads were de-multiplexed based on the experimental barcode on positions 4 to 7 using Flexbar (version 3.0.0) [62] without allowing mismatches.

All following steps of the analysis were performed on all individual samples after de-multiplexing. Remaining adapter sequences were trimmed from the right end of the reads using Flexbar (version 3.0.0) allowing up to one mismatch in 10 nt, requiring a minimal overlap of 1 nt of read and adapter. After trimming off the adapter, the barcode is trimmed off of the left end of the reads (first 9 nt) and added to the header of the read, such that the information is kept available for downstream analysis. Reads shorter than 15 nt were removed from further analysis.

Trimmed and filtered reads were mapped to the human genome (assembly version GRCh38) and its annotation based on GENCODE release 25 [63] using STAR (version 2.5.4b) [64]. When running STAR, up to two mismatches were allowed, soft-clipping was prohibited at the 5′ ends of reads, and only uniquely mapping reads were kept for further analysis.

Following mapping, duplicate reads were marked using the dedup function of bamUtil (version 1.0.13), which defines duplicates as reads whose 5′ ends map to the same position in the genome (https://github.com/statgen/bamUtil). Subsequently, marked duplicates with identical random barcodes were removed since they are considered technical duplicates, while biological duplicates showing unequal random barcodes were kept.

Resulting bam files were sorted and indexed using SAMtools (version 1.5) [65]. Based on the bam files, bedgraph files were created using bamToBed of the BEDTools suite (version 2.25.0) [66], considering only the position upstream of the 5′ mapping position of the read, since this nucleotide is considered as the crosslinked nucleotide. Bedgraph files were then transformed to bigWig file format using bedGraphToBigWig of the UCSC tool suite [67].

### Identification and characterization of MKRN1 binding sites

Peak calling was performed on merged iCLIP coverage tracks (crosslink events per nucleotide) from the three replicates based on GENCODE annotation (release 27, GRCh38) using ASPeak (version 2.0; default setting plus –nornaseq to estimate parameters *p* and *r* for the negative binomial distributions in a 500-nt window around each peak) [68]. The initially predicted peaks were resized to uniform 9-nt windows around their weighted centered as defined by ASPeak. To avoid artifacts, we removed sparsely covered peaks that harbor crosslink events on less than three nucleotides within the 9-nt region window. We iteratively merged all remaining windows if overlapping by at least 1 nt, by defining the position with the cumulative half maximum count of crosslink events as new window center. We then excluded all windows overlapping with none or multiple protein-coding genes (GENCODE annotations support level ≥ 2 and transcript support level ≥ 3) and assigned each binding site to a distinct genomic region (3′ UTR, 5′ UTR, CDS, intron). Consistent with the mostly cytoplasmic localization of MKRN1 [18, 19, 21], less than 6% of the binding sites were predicted within introns, which were excluded from further analysis. Finally, we kept only reproducible binding sites with at least three crosslink events in all three replicates. This procedure yielded a total of 7331 MKRN1 binding sites in 2163 genes (Additional file 1: Figure S4A).

In order to estimate binding site strength and to facilitate comparisons between binding sites (Fig. 2c, e, Additional file 1: Figure S4E-G and S5A,C,D), we corrected for transcript abundance by representing the crosslink events within a binding site as a “signal-over-background” ratio (SOB). The respective background was calculated as the sum of crosslink events outside of binding sites (plus 5 nt to either side) by the merged length of all exons. 3′ UTR lengths were restricted to 10 nt past the last MKRN1 binding site or 500 nt if no binding site was present. SOB calculations were performed separately for each replicate and then averaged. No SOB value was assigned for genes with a background of < 10 crosslink events, resulting in SOB values for 97% of all binding sites.

In order to assess the local RNA sequence context of MKRN1 binding sites (Fig. 2c and Additional file 1: Figure S5A), enriched 4-mers were counted inside the 9-nt binding sites as well as within 40-nt before and after. To estimate an empirical background distribution, 1000 9-nt windows were randomly picked in 3′ UTRs and 4-mer frequencies were counted in the same windows. This process was repeated 100 times, and the resulting mean and standard deviation were used to calculate the *z*-score for each 4-mer.

In order to define the A-rich regions downstream of MKRN1 binding sites in 3′ UTRs (A-rich stretches), we used a maximization approach in a 55-nt search space starting from the binding site center. Within this space, we calculated the percentage of A nucleotides (A-content) for windows of increasing size (8–30 nt) and selected the stretch with highest value for each window size. In case of ties, the window closer to the binding site was preferred, resulting in a set of 23 candidate A-rich stretches with the maximal A-content for each length. Next, we computed the longest continuous A-run (LCA) and a weighted A-content (multiplying the A-content with the number of A nucleotides) for each candidate A-rich stretch. Candidate A-rich stretches with an A-content < 70%, a weighted A-content < 11, and an LCA < 4 were excluded. The final A-rich stretch for each binding site was then selected in a hierarchical manner, preferring LCA over weighted A-content. Lastly, overlapping A-rich stretches of neighboring binding sites were merged by selecting the highest scoring A-rich stretch, based on LCA and weighted A-content. In total, this procedure identified 1412 non-overlapping A-rich stretches, associated with 1848 binding sites.

In order to estimate the extent of MKRN1 binding to poly(A) tails (Fig. 3a), we evaluated the percentage of adenosine within the iCLIP reads that could not be mapped to the human genome without soft-clipping (see above). iCLIP data for heterogeneous nuclear ribonucleoprotein H (HNRNPH) served as control [33]. To further support MKRN1 binding upstream of poly(A) tails, we counted the number of continuous A’s at the 3′ ends of all unmapped reads (Fig. 3a, inset).

Annotated transcript 3′ ends (i.e., polyadenylation sites) were taken from GENCODE (all annotated protein-coding transcripts with support level ≤ 2 and transcript support level ≤ 3; release 28, GRCh38.p12; https://www.gencodegenes.org/). For comparison, we included seven publicly available eCLIP datasets (ENCODE project) [34] from human K562 cells for the RBPs PABPC4 (ENCODE accession number ENCFF440SQF), UPF1 (ENCFF466HWF), PUM1 (ENCFF019LLG), HNRNPK (ENCFF924WZQ), QKI (ENCFF120WPV), CPSF6 (ENCFF420PXR), and TIAL1 (ENCFF430UQQ). For each RBP, we used the mate #2 fastq file from the replicate with more reads, which was processed as described above. For Fig. 3b, all crosslink events within a 2-kb window around the polyadenylation sites for 3′ UTR longer than 1 kb were counted. For each RBP, only 3′ UTRs with at least 10 crosslink events in 2-kb window were taken. The resulting crosslink profiles were normalized to the total number of considered regions for each RBP and smoothened with a running 50-nt window.

### RNA pulldown experiments

The RNA pulldown experiments were performed with 42-nt RNA oligonucleotides, containing 22 nt of the *SRSF4* 3′ UTR (Fig. 3c) until the cleavage site (chr1:29,147,886-29,147,907), including the proximal polyadenylation signal of *SRSF4*. This invariant part was followed, either by 20 A (A_20_ RNA) or by a control sequence of 20 V (Control RNA). The RNA oligonucleotides were ordered from IDT. Fifty-picomole RNA oligonucleotides per reaction were biotinylated using the Pierce RNA 3′ End Biotinylation Kit (Thermo Fisher Scientific) according to the manufacturer’s recommendations. The biotinylated oligonucleotides were bound to High Capacity NeutrAvidin agarose beads (Thermo Fisher Scientific). NeutrAvidin agarose beads that were not coupled to biotinylated oligonucleotides were used as controls. After washing, one third of RNA-bound beads was incubated with 2 μg recombinant His-MKRN1-WT and/or recombinant His-PABPC1, respectively, for 1 h at 4 °C in binding buffer (50 mM Tris-HCl pH 7.4, 500 mM KCl, 12.5 mM MgCl_2_, 1% Triton X-100). After three washes in washing buffer (200 mM NaCl, 10 mM MgCl_2_, 50 mM HEPES pH 7.4, 0.5% Igepal CA630, 1% Triton X-100), proteins were eluted from the beads by boiling in NuPAGE LDS sample buffer (Thermo Fisher Scientific) for 10 min at 70 °C. Proteins were analyzed by SDS-PAGE and Coomassie staining.

### Evolutionary characterization of Makorin protein family

Four different ortholog searches were performed using HaMStR-OneSeq [69] against the Quest for Orthologs Consortium protein set, containing 78 species (release 2017_04) [70]. For each run, a different seed protein was chosen: human MKRN1–3 (UniProt identifiers Q9UHC7, Q9H000, and Q13064) and MKRN4 from zebrafish (A9C4A6). In order to identify proteins with a similar domain architecture, we calculated a unidirectional feature architecture similarity (FAS) score which compares the domain architecture of the seed protein and the predicted ortholog [71]. Predicted orthologues with FAS < 0.7 were removed after initial assessment. Finally, all vertebrate species and selected invertebrate species were used for reconstruction of a maximum likelihood (ML) tree. For this, protein sequences were aligned using MAFFT v7.294b L-INS-i [72], and ML trees with 100 bootstrap replicates were calculated using RAxML version 8.1.9 [73]. Settings for a rapid bootstrap analysis and searching for the best scoring ML tree in one program run (-f a) and an automatic selection of the best fitting amino acid substitution model (-m PROTGAMMAAUTO) were chosen. Reconstructed trees were visualized using FigTree v1.4.2 (http://tree.bio.ed.ac.uk/ software/figtree/).

The phylogenetic tree and FASTA sequences from the ortholog dataset were loaded into DoMosaics [74], and Pfam domains were annotated with HMMER (http://hmmer.org/, default parameters). We additionally searched for the PAM2 motif that interacts with the MLLE domain of PABP proteins [75, 76].

Since the PAM2 motif in all Makorin proteins differs from the described consensus motif [22], a custom Hidden Markov Model was trained on PAM2 motifs from selected Makorin orthologs and used for a HMMER scan of the orthologs (no E-value cutoff). The same procedure was repeated for the recently reported PAM2-like motif (PAM2L) [25].

### Immunofluorescence microscopy

HEK293T cells were seeded on microscopy cover slips and transfected with GFP-tagged MKRN1^wt^ or MKRN1^PAM2mut^. Cells were washed with ice-cold PBS and fixed with 4% paraformaldehyde (Affymetrix) for 20 min. Cells were stained with DAPI (Sigma) and rinsed in PBS, wash buffer (10 mM Tris-HCl pH 7.5), and water. The samples were mounted with ProLong Diamond Antifade Mountant (Life Technologies). For analysis, a Leica SP5 II confocal system (Leica Microsystems) with a × 63 oil immersion NA1.4 objective lens was used, and two pictures were taken per frame. Images were processed in Fiji [77].

### Dual fluorescence translation stall assay via flow cytometry

Knockdowns were performed for 24 h, before the dual fluorescence reporter plasmids were ectopically expressed for 48 h. Cells were washed in PBS and trypsinized. After sedimentation, cells were resuspended in DPBS supplemented with 2 mM EDTA. Cellular GFP and RFP fluorescence was measured using flow cytometry on a LSRFortessa SORP (BD Biosciences). Data analysis was done using FlowJo (v10) (FlowJo, LLC). For statistical testing, paired two-tailed Student’s *t* tests with Benjamini-Hochberg correction were performed on *n* ≥ 6 replicates.

### Complementation approach

For complementation experiments, cell lines stably expressing siRNA2-insensitive *MKRN1* variants were used. To this end, HEK293T cells were transfected with plasmids coding for siRNA2-insensitive *MKRN1*^*wt*^, *MKRN1*^*PAM2mut*^, or *MKRN1*^*RINGmut*^ or an empty vector (EV) control using PEI as described above. Forty-eight hours after transfection, puromycin selection was started (1.5 μg/ml puromycin). Upon single-cell dilutions, cell lines that stably expressed *MKRN1*^*wt*^, mutants or EV were kept in DMEM containing 1.5 μg/ml puromycin. In the dual fluorescence translation stall assay via flow cytometry, cells were seeded in DMEM without puromycin for 24 h. Then, knockdowns were performed for 24 h, before the dual fluorescence reporter plasmids were ectopically expressed for 48 h. Cells were prepared and analyzed as described above.

### CRISPR/Cas9 knockout

The pSpCas9(BB)-2A-Puro (PX459) V2.0 backbone (Addgene #62988) was digested with BbsI, dephosphorylated, and gel-purified using the Qiagen QIAquick Gel Extraction Kit according to the manufacturer’s recommendations [78, 79]. *MKRN1* guide RNAs (see Additional file 1: Table S5) were designed using the ChopChop Website (http://chopchop.cbu.uib.no) [80, 81]. Phosphorylated oligonucleotides were annealed. Using T4 DNA ligase at 16 °C overnight, the oligonucleotides were ligated into the digested vector [78, 79]. The CRISPR-SpCas9 plasmid containing guide RNA targeting *MKRN1* was transfected into HEK293T cells. After 48 h, CRISPR-SpCas9-positive cells were selected using puromycin (1.5 μg/ml) for 5 days. *MKRN1* KO cells were subsequently cultured in DMEM with 10% fetal bovine serum, 1% penicillin/streptomycin, and 1% l-glutamine. Upon single-cell dilutions, MKRN1 protein levels of these cells were assessed by Western Blot, and *MKRN1* mRNA levels were analyzed by qPCR.

### Ubiquitin remnant profiling

Di-glycine remnant profiling was performed as described before [82, 83]. In four different experiments, isotope labels were assigned as follows: experiment 1, *MKRN1* KD1 (siRNA1), *MKRN1* KD2 (siRNA2), and control siRNA with light, medium, and heavy SILAC labels, respectively; experiment 2, *MKRN1* KD2 (siRNA2) and control siRNA with heavy and light SILAC labels, respectively; experiment 3, *MKRN1* KD2 (siRNA2) and control siRNA with heavy and light SILAC labels, respectively; experiment 3, *MKRN1* KD2 (siRNA2) and control siRNA with light and heavy SILAC labels, respectively. Cells were treated with the proteasome inhibitors bortezomib (1 μM, 8 h, replicate 1; Santa Cruz Biotechnology) or MG132 (10 μM, 2 h, replicates 2, 3, 4; Sigma). Proteins were precipitated in acetone. Proteins were digested with endoproteinase Lys-C (Wako Chemicals) and sequencing-grade modified trypsin (Sigma). To purify the peptides, reversed-phase Sep-Pak C18 cartridges (Waters) were used. Modified peptides were enriched using di-glycine-lysine antibody resin (Cell Signaling Technology). The enriched peptides were eluted with 0.15% trifluoroacetic acid in water, then fractionated using micro-column-based strong-cation exchange chromatography (SCX) [84] before being desalted on reversed-phase C18 StageTips [49]. Samples were analyzed by quantitative mass spectrometry and MaxQuant as described above. To identify significantly regulated ubiquitylation sites, the limma algorithm was applied [56]. A *P* value < 0.1 after multiple testing correction was used as a cutoff to determine up- and downregulated ubiquitylation sites. Volcano and dot plots were created in R (version 3.4.3).

### Functional interaction network of MKRN1 ubiquitylation target proteins

The functional protein interaction network analysis was performed by integrating interaction data from the STRING database (score > 0.4), the BioGrid database, and our own findings [85, 86]. Cytoscape (version 3.6.1) was used to visualize the protein interaction network [87].

## Supplementary information

**Additional file 1:** **Figure S1.** Maximum likelihood tree of Makorin orthologs with their protein domain architecture. **Figure S2.** MKRN1 interacts with translational regulators and other RBPs. **Figure S3.** GFP-MKRN1^RINGmut^ interacts with PABPC1/4 and RPS10. **Figure S4.** Signal-over-background transformation allows to estimate MKRN1 binding site strength. **Figure S5.** MKRN1 binds upstream of A-rich stretches. **Figure S6.** Interaction with PABPC1 is required for MKRN1 RNA binding. **Figure S7.** MKRN1 is required to stall ribosomes at K(AAA)_20_ in reporter assays. **Figure S8.** Cross-regulation of MKRN1 and ZNF598. **Figure S9.** Proteome analysis upon *MKRN1* KD and GO term analysis of MKRN1 ubiquitylation targets. **Table S2.** Summary of MKRN1 iCLIP experiments. **Table S5** Oligonucleotides used in this study. **Table S6** siRNAs used in this study. (PDF 2450 kb)

**Additional file 2:** **Table S1.** with MaxQuant analysis of MS data from the SILAC-AP for GFP-MKRN1^wt^, GFP-MKRN1^PAM2mut^, and GFP-MKRN1^RINGmut^. (XLSX 1409 kb)

**Additional file 3:** **Figures S10-S12.** with images of full membranes and different exposure times for Western blots and other analyses as specified. (PDF 2348 kb)

**Additional file 4:** **Table S3.** with MaxQuant analysis of MS data from the ubiquitin remnant profiling from *MKRN1* KD2 in HEK293T cells. (XLSX 7940 kb)

**Additional file 5:** **Table S4.** with MaxQuant analysis of MS data from the proteome analysis from *MKRN1* KD2 in HEK293T cells. (XLSX 2270 kb)

**Additional file 6:** Review history. (DOCX 34 kb)

## Data Availability

Dataset supporting the conclusions of this article are available at the ProteomeXchange Consortium (http://proteomecentral.proteomexchange.org/cgi/GetDataset?ID=PXD011772) [88] via the PRIDE partner repository with the identifier PXD011772 (https://www.ebi.ac.uk/pride/archive/projects/PXD011772) (proteomics) and the Gene Expression Omnibus under the accession number GSE122869 (https://www.ncbi.nlm.nih.gov/geo/query/acc.cgi?acc=GSE122869) (iCLIP) [89]. eCLIP datasets analyzed during the current study were retrieved from the ENCODE data portal (https://www.encodeproject.org/) [34] via accession numbers ENCFF440SQF (PABPC4), ENCFF466HWF(UPF1), ENCFF019LLG (PUM1), ENCFF924WZQ (HNRNPK), ENCFF120WPV (QKI), ENCFF420PXR (CPSF6), and ENCFF430UQQ (TIAL1).
