# Supplementary material for: The RNA-binding ubiquitin ligase MKRN1 functions in ribosome-associated quality control of poly(A) translation
Source: Genome Biol. 2019 Oct 22;20:216. doi: 10.1186/s13059-019-1814-0 (PMC6805484; doi:10.1186/s13059-019-1814-0)
Supplement: Supplementary file 1 — Additional file 1: Figure S1. Maximum likelihood tree of Makorin orthologs with their protein domain architecture. Figure S2. MKRN1 interacts with translational regulators and other RBPs. Figure S3. GFP-MKRN1RINGmut interacts with PABPC1/4 and RPS10. Figure S4. Signal-over-background transformation allows to estimate MKRN1 binding site strength. Figure S5. MKRN1 binds upstream of A-rich stretches. Figure S6. Interaction with PABPC1 is required for MKRN1 RNA binding. Figure S7. MKRN1 is required to stall ribosomes at K(AAA)20 in reporter assays. Figure S8. Cross-regulation of MKRN1 and ZNF598. Figure S9. Proteome analysis upon MKRN1 KD and GO term analysis of MKRN1 ubiquitylation targets. Table S2. Summary of MKRN1 iCLIP experiments. Table S5 Oligonucleotides used in this study. Table S6 siRNAs used in this study. (PDF 2450 kb) [file 13059_2019_1814_MOESM1_ESM.pdf]

Fig. S1

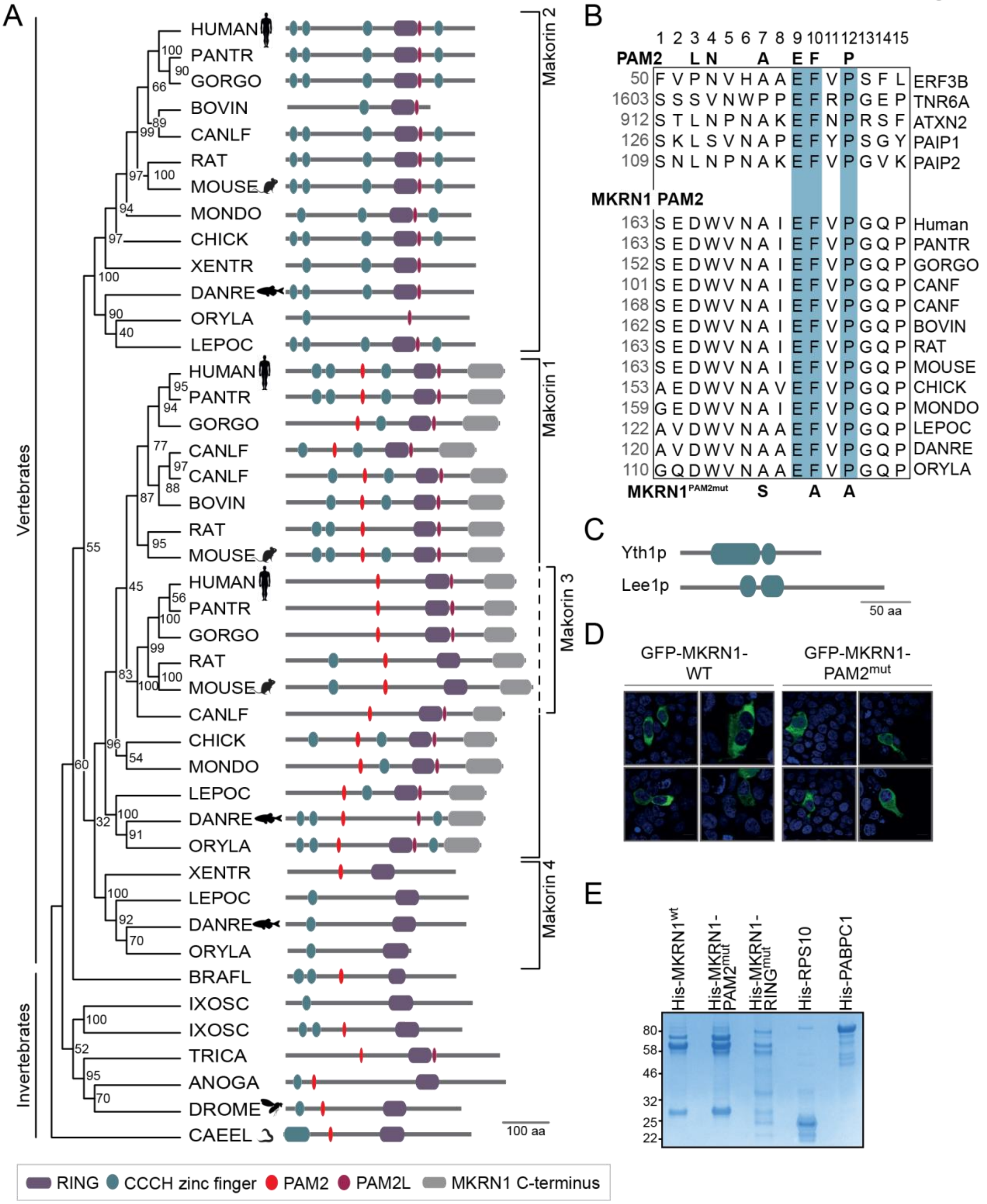

Fig. S1. Maximum likelihood tree of Makorin orthologs with their protein domain architecture. (*A*) Maximum likelihood tree with 100 bootstrap replicates of selected vertebrate and invertebrate orthologs and *C. elegans* as an outgroup. Bootstrap values at each node indicate the number of replicates (out of 100) that support the local tree structure and thereby serve as confidence estimates. Protein schematics (drawn to scale) on the right depict protein domains corresponding to the following PFAM domains: RING-type zinc finger, PF13445; MKRN1 C-terminus, PF15815; CCCH zinc finger, PF15663, PF14608 and PF00642. PAM2 motifs, predicted to interact with the MLLE domain of PABP proteins [1] as well as the recently reported derivative PAM2L

[2], were added separately (see Material and methods). Abbreviated and full species names with corresponding UniProt identifiers in order of appearance: ANOGA, *Anopheles gambiae*, Q7QF83; BOVIN, *Bos taurus*, F1MF12, F6QQR5; BRAFL, *Branchiostoma floridae*, C3Y7M0; CAEEL, *Caenorhabditis elegans*, Q9N373; CANLF, *Canis lupus,* J9P921, E2RRA5, E2REH2, J9P9K3; DANRE, *Danio rerio,* Q4VBT5, Q9DFG8, A9C4A6; DROME, *Drosophila melanogaster,* Q9VP20; CHICKEN, *Gallus gallus,* Q9PTI4, F1NI93; GORGO, *Gorilla gorilla*, G3S6Y3, G3QDU4, G3RZ99; HUMAN, *Homo sapiens*, Q9UHC7, Q9H000, Q13064; IXOSC, *Ixodes scapularis*, B7QIJ9, B7Q4B2; LEPOC, *Lepisosteus oculatus*, W5NGW8, W5N9B2, W5LWJ1; MONDO, *Monodelphis domestica*, F6QPR3, F7F0I3; MOUSE, *Mus musculus*, Q9QXP6, Q9ERV1, Q60764; ORYLA, *Oryzias latipes*, H2MBR3, H2M1P4, H2LQG1; PANTR, *Pan troglodytes*, H2QVH8, H2QM29, H2Q915; RAT, *Rattus norvegicus*, A0A0G2QC40, Q5XI23, D3ZY41; XENTR, *Xenopus tropicalis*, Q6GLD9, B4F720. (*B*) The PAM2 motif in Makorin proteins from vertebrates (bottom, species abbreviations as in (*A*)) shows similarities to PAM2 in known PABPC1-interacting proteins from human (top, protein names given; first amino acid position for all PAM2 motifs indicated on the left in grey). The PAM2 consensus [1] is given above. Positions 9, 10 and 12 within the aligned regions that are highly consistent between all aligned proteins and important for PAM2 function [3] are highlighted in brown. Mutations that were introduced to abrogate the function of the PAM2 motif in human MKRN1 (MKRN1^{PAM2mut}) are shown below. The corresponding UniProt identifiers are Q8IYD1, Q8NDV7, Q99700, Q9H074, Q9BPZ3 (known PABPC1-interacting proteins from human), Q9UHC7, H2QVH8, G3S6Y3, J9P921, E2RRA5, F1MF12, Q5XI23, Q9QXP6, Q9PTI4, F6QPR3, W5NGW8, Q4VBT5, H2MBR3 (Makorin orthologs from vertebrates). (*C*) The closest Makorin orthologs in *Saccharomyces cerevisiae* lack RING domain and PAM2 motif. Domain architecture of Yth1p and Lee1p, which were detected as closest orthologs by HaMStR-OneSeq [4], but were not considered as orthologs due to low FAS scores (0,59 and 0,60, respectively). The annotated PFAM domains are CCCH zinc finger, PF15663, PF00642, PF16131. (*D*) GFP-MKRN1^{wt} and GFP-MKRN1^{PAM2mut} localise mainly to the cytoplasm, with a small fraction being observed in the nucleus [5]. Confocal microscopy of HEK293T cells ectopically expressing GFP-MKRN1^{wt} and GFP-MKRN1^{PAM2mut}. Scale bars indicate 10 µm. (*E*) Recombinantly expressed proteins from *E. coli* were analysed by SDS-PAGE and Coomassie staining. Uncropped gel image is shown in **Additional file 3: Fig. S10I**.

Fig. S2

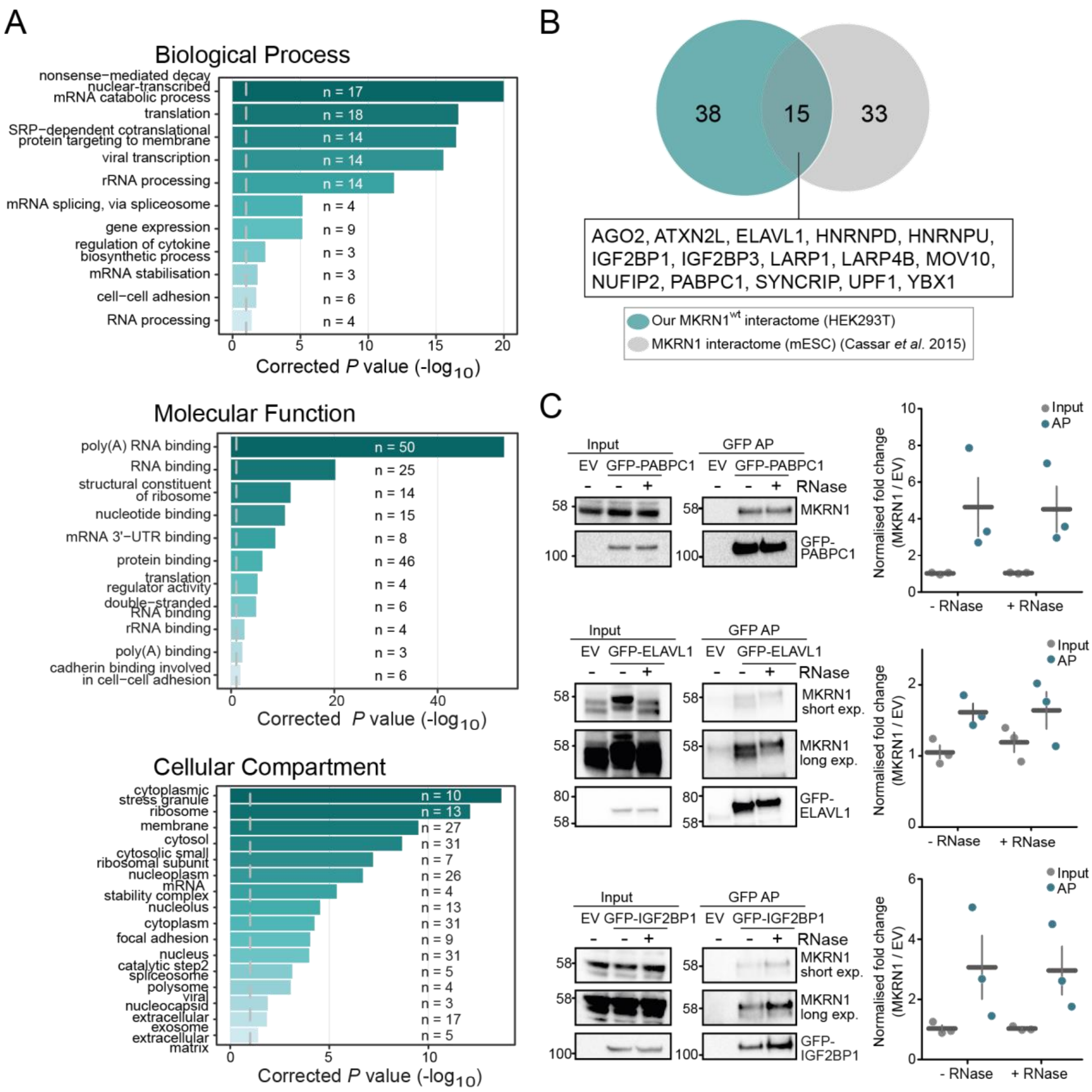

Fig. S2. MKRN1 interacts with translational regulators and other RBPs. (*A*) GO terms enriched for the 53 MKRN1 interactors. *P* values (modified Fisher exact test, Benjamini-Hochberg correction) are depicted for all significant GO terms (corrected *P* value < 0.05) for Biological Process, Molecular Function and Cellular Compartment, together with the number of interactors associated with the respective term. (*B*) Overlap of the 53 significant interaction partners of GFP-MKRN1^{wt} in human HEK293T cells with previously published interactors of MKRN1 in mouse embryonic stem cells (mESC) [6]. (*C*) Reciprocal APs show that MKRN1 interacts with PABPC1, ELAVL1 and IGF2BP1 independently of RNA. AP with GFP-PABPC1, GFP-ELAVL1 and GFP-IGF2BP1 as baits were performed from HEK293T cells in the presence or absence of RNase A and T1. Bait proteins and endogenous MKRN1 were detected by Western blots (replicate 1). Different exposure times (exp.) for MKRN1 are shown for GFP-ELAVL1 and GFP-IGF2BP1 APs. Quantifications (fold changes of the MKRN1 signal over empty vector, EV) of three replicates are shown on the right. Replicates and uncropped gel images are shown in **Additional file 3: Fig. S10J-R**.

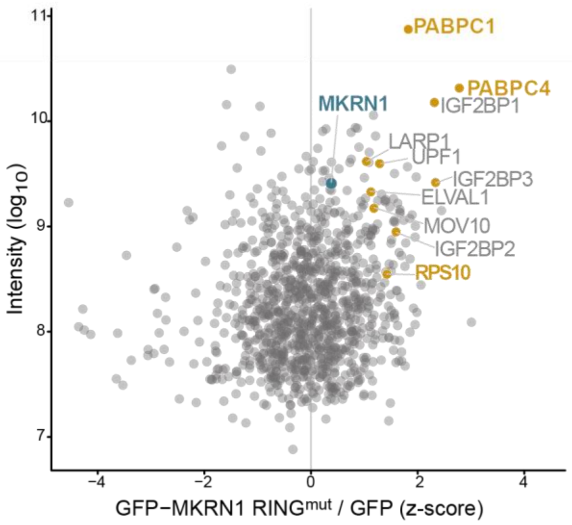

Fig. S3. GFP-MKRN1^{RINGmut} interacts with PABPC1/4 and RPS10. Experiments in HEK293T cells for GFP-MKRN1^{RINGmut} were performed using SILAC-based mass spectrometry. Asymmetrical z-scores of combined SILAC ratios (n = 3 replicates) are plotted against log_{10}-transformed intensities. 1,097 protein groups were quantified in at least two out of three replicates (**Additional file 2: Table S1**). MKRN1 and interesting interaction partners are highlighted.

Fig. S4

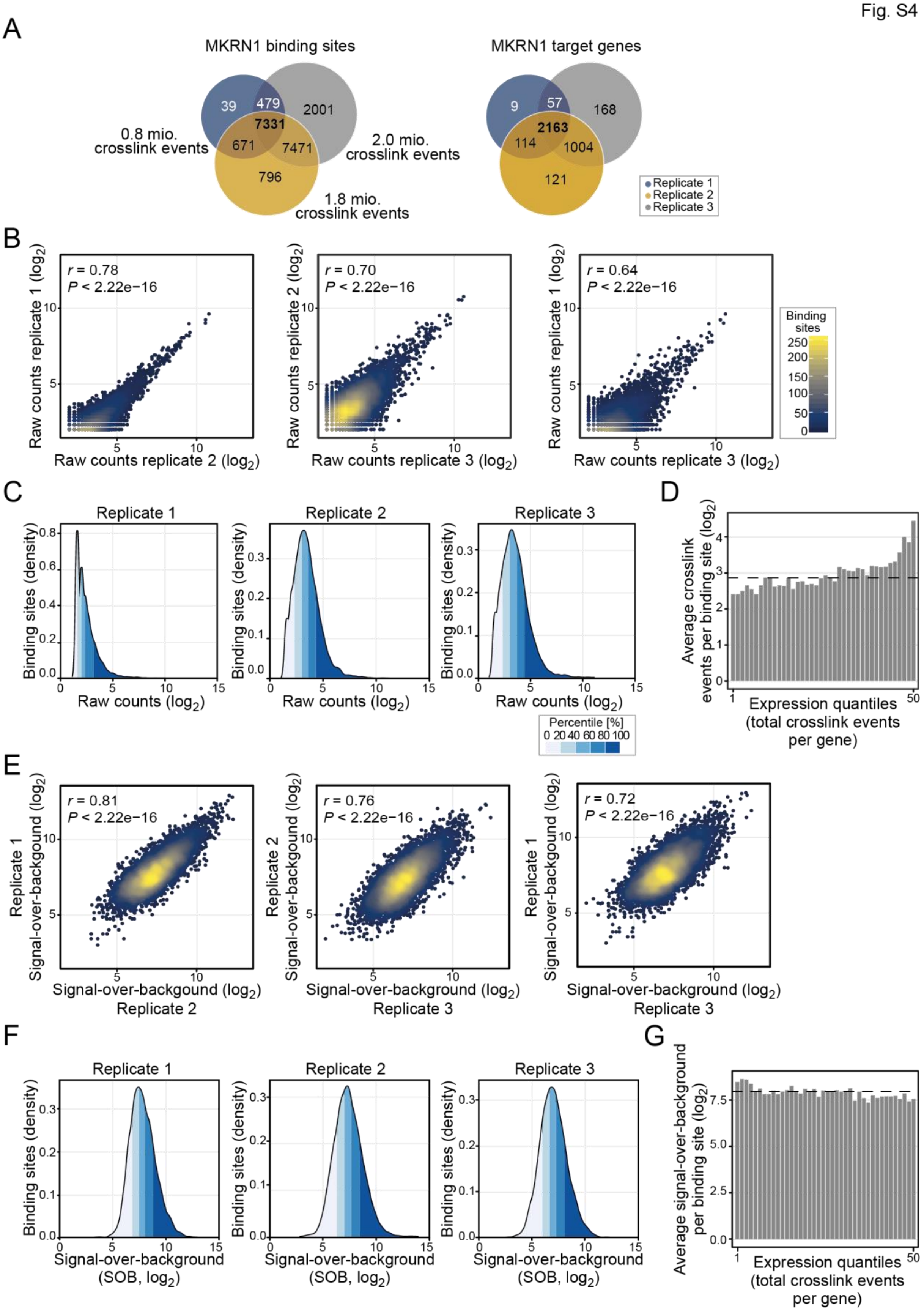

Fig. S4. Signal-over-background transformation allows to estimate MKRN1 binding strength. (*A*) Venn diagramms depict the overlap of binding sites that capture at least three crosslink events in a given replicate (left) and of the associated genes (right).

Total crosslink events in each replicates is given on the sides (**Additional file 1: Table S2**). (*B-D*) Raw iCLIP signal before signal-over-background transformation. (B) Scatter plots show pairwise comparisons of crosslink events per binding site in three replicate MKRN1 iCLIP experiments. Pearson correlation coefficients (*r*) and associated *P* values are given. (*C*) Density plots depict the distribution of crosslink events per binding site in the three replicate experiments. Shades of blue indicate 20% quantiles; top 20% of binding sites with highest counts are denoted by a dashed line. (*D*) Raw iCLIP counts are strongly influenced by the expression level of the underlying gene. MKRN1-bound genes were stratified into 50 bins with increasing expression (using the total number of MKRN1 crosslink events within the 3' UTR as a proxy of a gene's expression level). Shown is the average number of crosslink events per binding site for all binding sites in each bin. Dashed line denotes median across all bins. (*E-G*) Signal-over-background (SOB) values allow to correct for expression-level differences. (*E*) Pairwise comparison of SOB values for the three MKRN1 iCLIP replicate experiments. Scatter plots as in (*B*). (*F*) Distribution of SOB values in the three replicates. Density plots as in (*C*). Shades of blue indicate 20% quantiles. Dashed lines denote the top 20% MKRN1 binding sites with strongest binding that were used for the analyses in **Fig. 2B** and **Additional file 1: Fig. S4A**. (*G*) SOB values are independent of the expression level of the underlying gene. Average SOB values for all binding sites in each expression bin are shown as in (*D*).

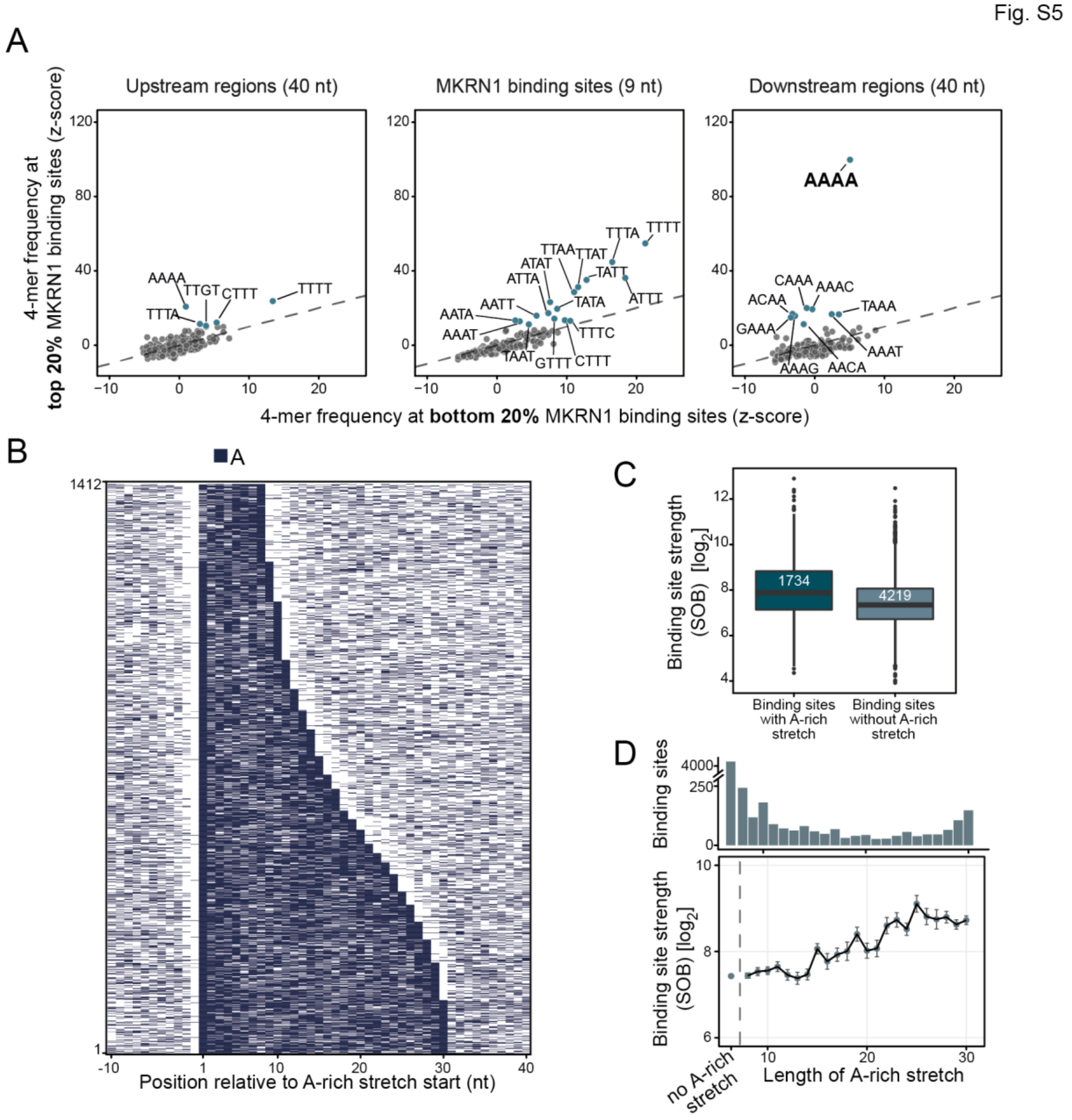

Fig. S5. MKRN1 binds upstream of A-rich stretches. (*A*) The top 20% MKRN1 binding sites show a strong RNA binding preference for AAAA. Scatter plot compares the frequency of 4-mers within the 9-nt MKRN1 binding sites and flanking 40-nt windows for the top 20% and bottom 20% MKRN1 binding sites (according to signal-over-background, SOB). 4-mer frequencies are displayed as z-scores based on background distribution from binding site permutations. (*B*) Heatmap representation of 1,412 non-overlapping A-rich stretches at MKRN1 binding sites, sorted by increasing length (8-30 nt). Only A's are coloured. (*C*) Binding sites with associated A-rich stretches show stronger MKRN1 binding. Boxplot compares the SOB values of MKRN1 binding sites in 3' UTRs with and without associated A-rich stretches. Number of binding sites indicated inside box. (*D*) MKRN1 binding site strength (SOB) increases with length of associated A-rich stretch. Mean and standard deviation of MKRN1 binding sites associated with A-rich stretches of increasing length (x-axis). MKRN1 binding sites without associated A-rich stretches are shown for comparison on the left. Number of binding sites in each category indicated as barchart above.

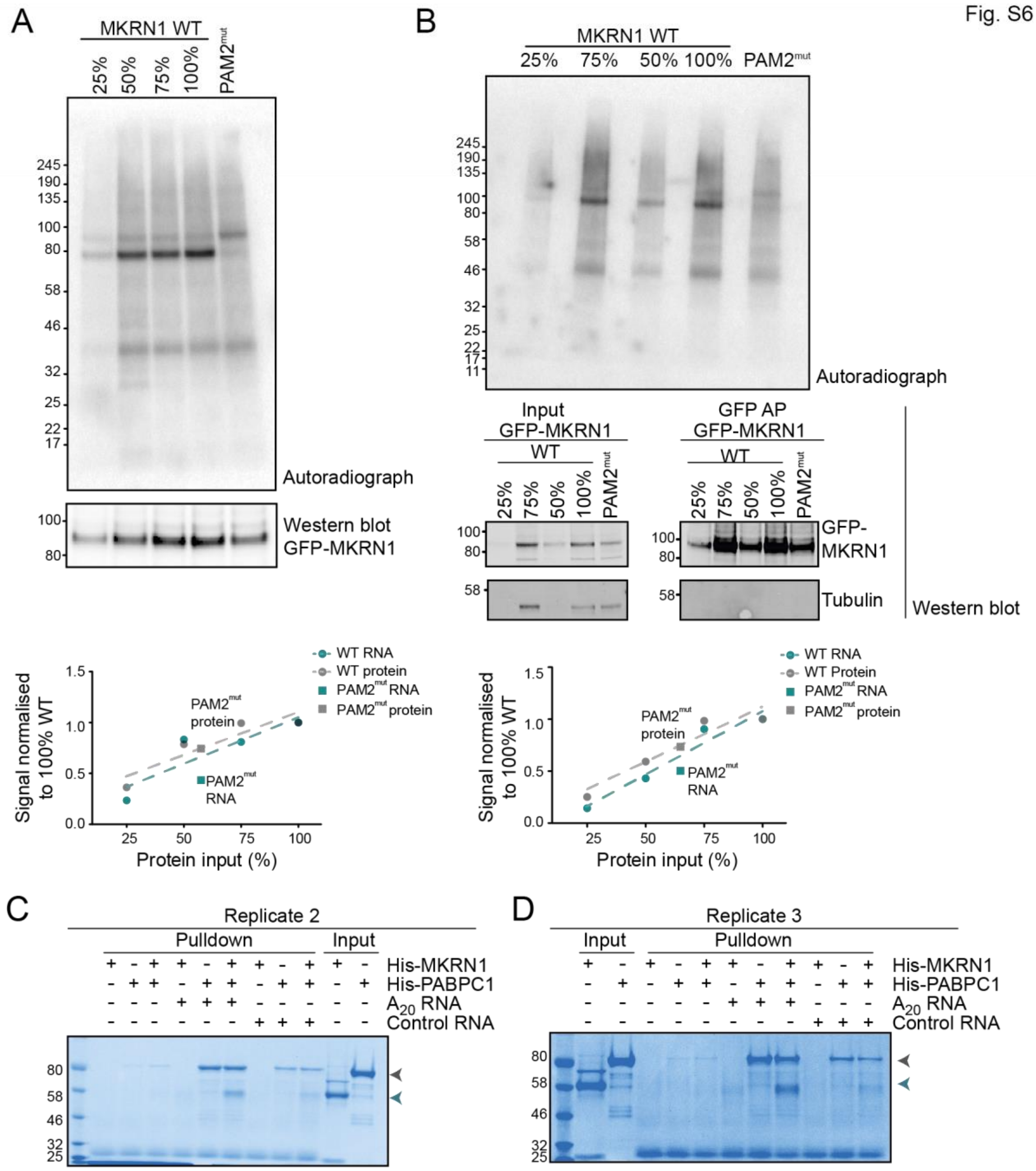

Fig. S6. Interaction with PABPC1 is required for MKRN1 RNA binding. (*A,B*) UV crosslinking experiment to measure the RNA binding of GFP-MKRN1^{wt} and GFP-MKRN1^{PAM2mut}. Autoradiographs (top) and Western blots (bottom) show GFP-MKRN1/RNA complexes and GFP-MKRN1 protein, respectively, in the eluates from replicates 2 (with 4SU and UV crosslinking at 365 nm) (*A*) and 3 (with conventional UV crosslinking at 254 nm) (*B*). For calibration, input samples for GFP-MKRN1^{wt} were diluted to 75%, 50% and 25% prior to GFP AP. Note that samples were loaded in different order in (*B*). Quantifications are given below. Uncropped gel images in **Additional file 3: Fig. S11A,B**. (*C,D*) MKRN1 is recruited to poly(A) RNA with the help of PABPC1. SDS-PAGE (Coomassie staining) shows recovery of recombinant His-MKRN1^{wt} (petrol) and/or His-PABPC1 (grey) from pulldown of bead-coupled RNA oligonucleotides, with the last 22 nt of the *SRSF4* 3' UTR and 20 A (A_{20} RNA) or 20 V nucleotides (Control RNA). Beads without RNA served as controls. Protein binding to RNA was analysed by SDS-PAGE and Coomassie staining. Replicates 2 (*C*) and 3 (*D*) from **Fig. 3E**. Uncropped gel images in **Additional file 3: Fig. S11C-E**.

Fig. S7

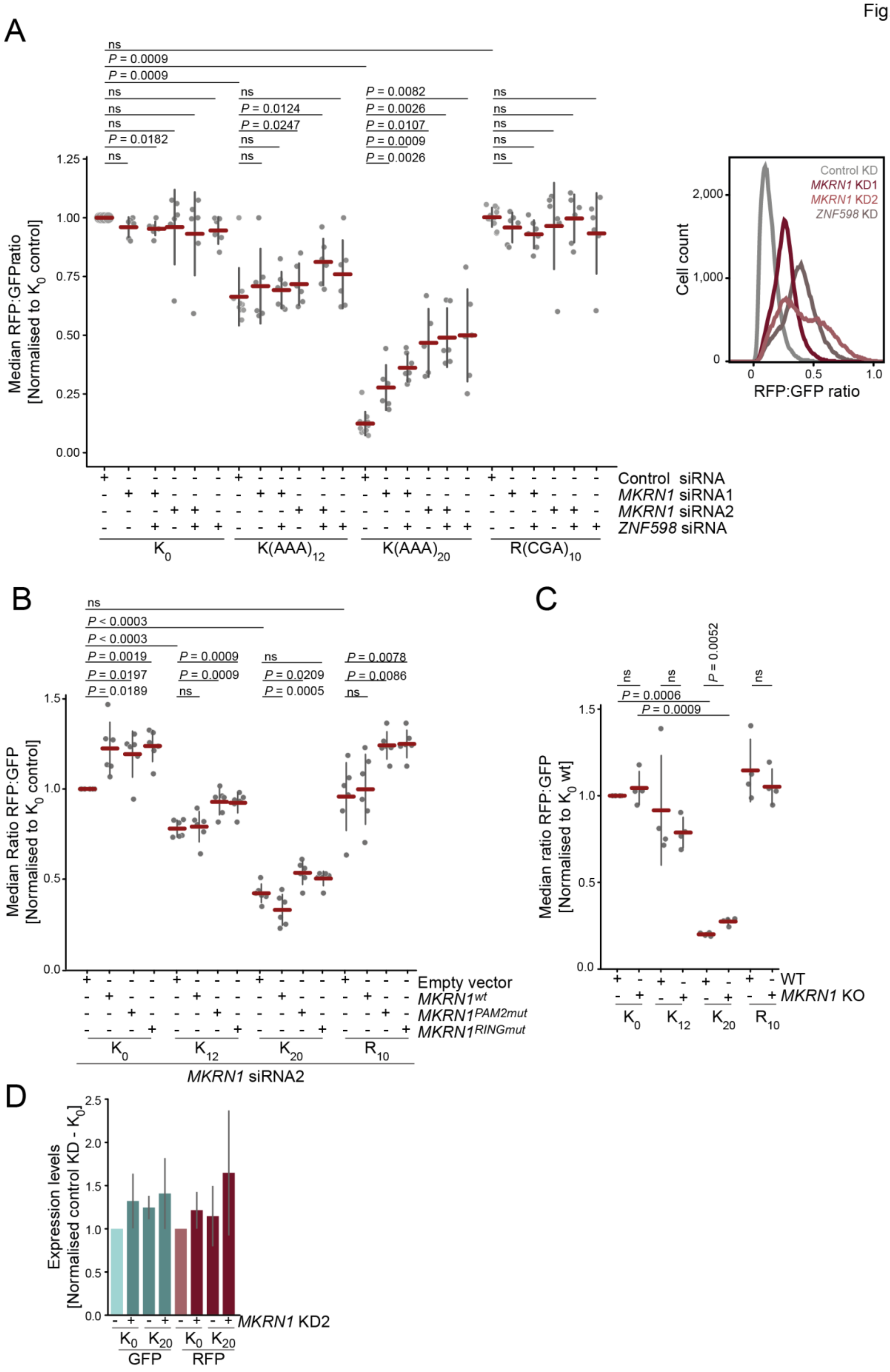
A
Median RFP:GFPratio [Normalised to K_0 control]
Control siRNA
MKRN1 siRNA1
MKRN1 siRNA2
ZNF598 siRNA
K_0
$K(AAA)_{12}$
$K(AAA)_{20}$
$R(CGA)_{10}$
Control KD
MKRN1 KD1
MKRN1 KD2
ZNF598 KD
Cell count
RFP:GFP ratio
B
Median Ratio RFP:GFP [Normalised to K_0 control]
Empty vector
$MKRN1^{wt}$
$MKRN1^{PAM2mut}$
$MKRN1^{RINGmut}$
K_0
K_{12}
K_{20}
R_{10}
MKRN1 siRNA2
C
Median ratio RFP:GFP [Normalised to K_0 wt]
WT
MKRN1 KO
D
Expression levels [Normalised control KD - K_0]
MKRN1 KD2
GFP
RFP

Fig. S7. MKRN1 is required to stall ribosomes at $K(AAA)_{20}$ in reporter assays. (*A*) Translation of dual fluorescence reporter plasmids was assessed by flow cytometry upon *MKRN1* and/or *ZNF598* KD. Median RFP:GFP ratios (normalised to K_0 in control KD) are shown for the reporter plasmids K_0, $K(AAA)_{12}$, $K(AAA)_{20}$, and $R(CGA)_{10}$. Error bars represent standard deviation of the mean (s.d.m., $n \geq 6$ replicates; paired two-tailed Student's t-test, Benjamini-Hochberg correction). Density plot of median RFP:GFP ratios of one replicate experiment with $K(AAA)_{20}$ with control or *MKRN1* KD (two independent siRNAs, KD1 and KD2) or *ZNF598* is shown on the right. (*B*) Translation of dual fluorescence reporter plasmids was assessed in HEK293T cells stably expressing MKRN1 variants by flow cytometry upon *MKRN1* KD2. Median RFP:GFP ratios (normalised to K_0 in control KD) are shown for the reporter plasmids K_0, $K(AAA)_{12}$, $K(AAA)_{20}$, and $R(CGA)_{10}$. Visualisation as in (*A*). (*C*) Translation of dual fluorescence reporter plasmids was assessed by flow cytometry upon *MKRN1* KO. Median RFP:GFP ratios (normalised to K_0 in wild type, WT) are shown for the reporter plasmids K_0, $K(AAA)_{12}$, $K(AAA)_{20}$, and $R(CGA)_{10}$. Visualisation as in (*A*). (*D*) mRNA levels are not affected from ribosome stalling in HEK293T cells. mRNA levels of GFP and RFP of the K_0 and $K(AAA)_{20}$ reporter plasmids were analysed by qPCR in control and *MKRN1* KD2 cells in relation to neomycin levels ($n = 3$ replicates). Error bars indicate s.d.m..

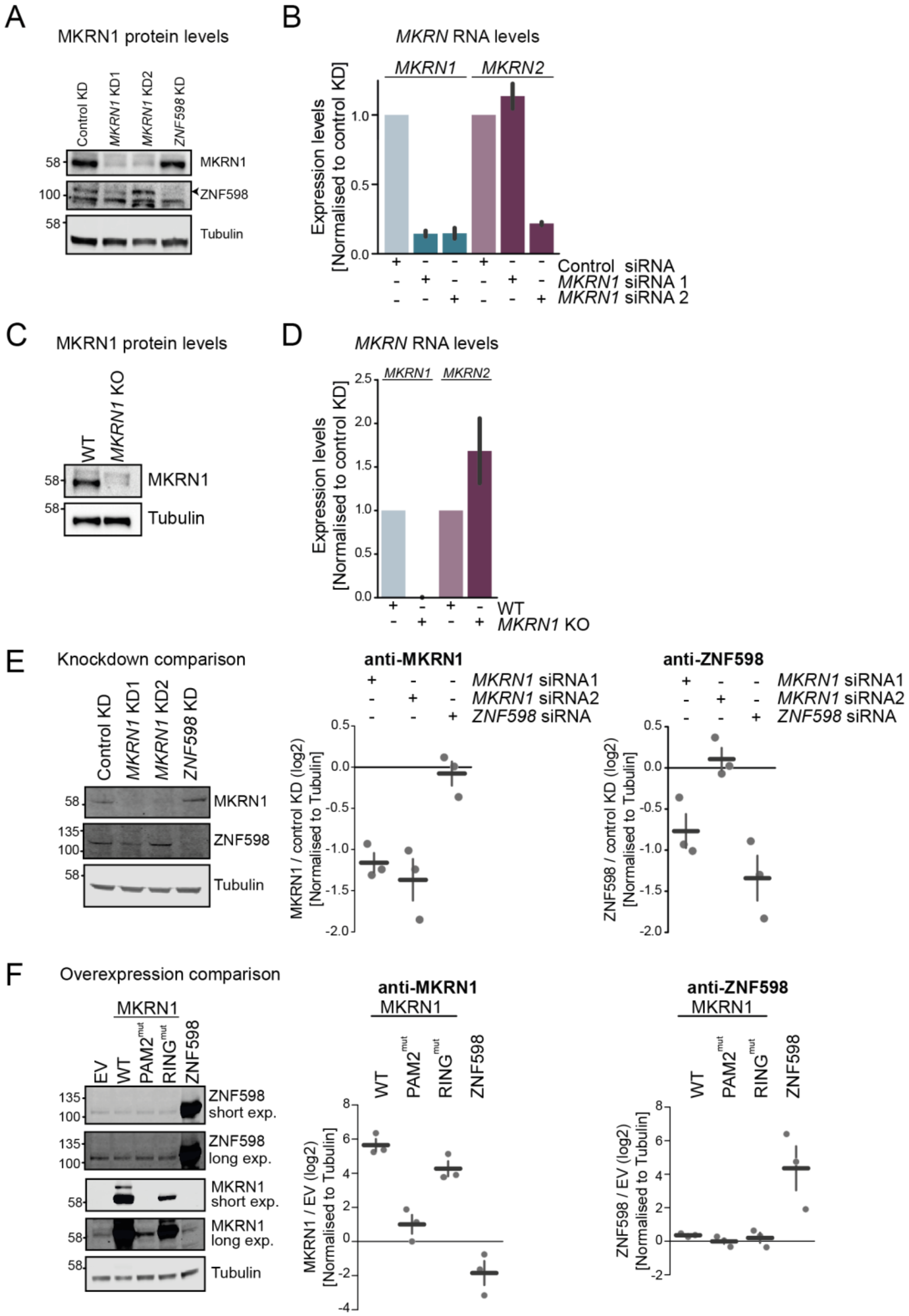

Fig. S8. (*A*) KDs of *MKRN1* and *ZNF598* were assessed by Western blot (n = 3 replicates). Black arrowhead indicates ZNF598. Replicates and uncropped gel images are shown in **Additional file 3: Fig. S12B,C**. (*B*) *MKRN1* KD2 also reduces *MKRN2* levels. Expression levels of *MKRN1* and *MKRN2* were assessed in relation to *ß-actin* levels by qPCR in *MKRN1* KDs (two independent siRNAs, KD1 and KD2) and control KD cells. *MKRN1* KD1 and KD2 were performed for 72 h. Error bars indicate s.d.m. (n = 2 replicates). (*C*) MKRN1 protein levels were assessed by Western blot in *MKRN1* KO and wild type (WT) HEK293T cells. Western blot analysis was performed with

antibodies against MKRN1 and tubulin. Replicates and uncropped gel images are shown in **Additional file 3: Fig. S12A**. (*D*) *MKRN2* levels are elevated in *MKRN1* KO cells. Expression levels of *MKRN1* and *MKRN2* were assessed in *MKRN1* KO and WT HEK293T cells in relation to *HIST1H1E* levels by qPCR (n = 3 replicates). Error bars indicate s.d.m. (*E,F*) Cross-regulation of MKRN1 and ZNF598. (*E*) *MKRN1* KD1 reduces endogenous ZNF598 protein levels. Effect of *MKRN1* KD1, KD2 and *ZNF598* KD for 72 h was assessed by Western blot for endogenous MKRN1 and ZNF598. Quantifications depict MKRN1 or ZNF598 expression levels in *MKRN1* KD or *ZNF598* KD over control KD, normalised to tubulin levels (n = 3 replicates). Replicates and uncropped gel images are shown in **Additional file 3: Fig. S12D,E.** (*F*) *ZNF598* overexpression reduces MKRN1 protein levels. Effect of *ZNF598* and *MKRN1* (WT and mutants) overexpression was tested after 48 h. Quantification as in (*E*). Uncropped gel images for all replicates are in **Additional file 3: Fig. S12F,G**.

Fig. S9

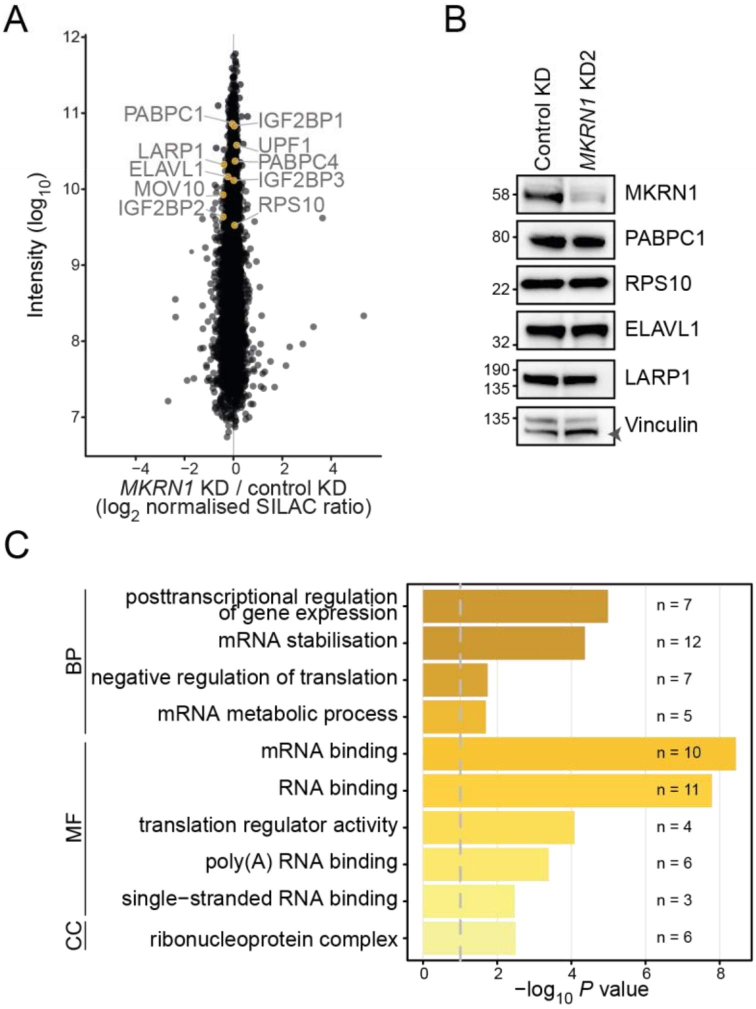

Fig. S9. Proteome analysis upon *MKRN1* KD and GO term analysis of MKRN1 ubiquitylation targets. (*A*) Proteome analysis of *MKRN1* KD2 in HEK293T cells analysed by quantitative mass spectrometry. Log_2-transformed, combined normalised SILAC ratios (n = 3 replicates) are plotted against log_{10}-transformed intensities. 6,425 protein groups were quantified in at least one out of three replicate experiments (**Additional file 5: Table S4**). Selected ubiquitylation targets of MKRN1 are highlighted. (*B*) Total levels of proteins that are ubiquitylated in a MKRN1-dependent manner do not change in *MKRN1* KD2 conditions. Protein levels of MKRN1, PABPC1, RPS10, ELAVL1, LARP1, and vinculin were analysed by Western blot. Vinculin is indicated by a grey arrowhead. Replicates 2 and 3, and uncropped gel images are in **Additional file 3: Fig. S12H**. (*C*) GO terms enriched for the 21 MKRN1 ubiquitylation targets. Corrected *P* values (modified Fisher's exact test, Benjamini-Hochberg correction) are depicted for all significant GO terms (corrected *P* value < 0.05) for Biological Process (BP), Molecular Function (MF) and Cellular Compartment (CC), together with the number of ubiquitylation targets associated with the respective term.

Table S2: Summary of MKRN1 iCLIP experiments. iCLIP experiments with GFP-MKRN1 were performed in three independent replicates.

	Sequenced reads	Uniquely mapped reads	Crosslink events	Binding sites
Replicate 1	3,418,021	1,374,295	844,307	-
Replicate 2	6,527,256	2,805,111	1,759,893	-
Replicate 3	4,660,274	2,258,913	2,058,204	-
**Total**	14,605,551		4,662,404	**7,331**

Table S5: Oligonucleotides used in this study.

Name	Sequence 5' – 3'	Comment
PAMmut	GCCGTTGCCGGGCAACCCTACTGTGGC	MKRN1^{PAM2mut} mutant
	CTCAATAGAATTCACCCAGTCCTCTGAACC	
H307E	CAACTGCAACGAAACCTACTGTCTCAAG	MKRN1^{RINGmut} mutant
	GAGAGGATCCCGAAGCGG	
siRNA2-insensitive	[P][P]tctaattgtaatCACACCTACTGTCTCAAGTGCATTCG	MKRN1 siRNA2-insensitive mutant
	[P][P]cagaatgccaaaGCGGCGCTCACTGGGGTT	
pMX-del-GFP	GAGCTCTACAAAAGCGGTTC	*Deletion of GFP from pMX-DEST53-GFP vector*
	GCTGGCCATGGTGTCTAG	
MKRN1 qPCR	CGATACGGGGAGAACTGTGT	*MKRN1* qPCR primer
	CCTTCTCATGGGCCTCAAT	
MKRN2 qPCR	ACTCACATGAACCCGGAAAG	*MKRN2* qPCR primer
	AGCTGCCTGGATTACTCACC	
ZNF598 qPCR	AACCTCGACAAATGGTCCTG	*ZNF598* qPCR primer
	GTCTTCGTCCTTGAGCTTCG	
ßActin qPCR	TCCTCCCTGGAGAAGAGCTAC	*ß-actin* qPCR primer
	TGGAGTTGAAGGTAGTTCGTG	
HIST1H1E qPCR	AAAGAAGGCGAAGAAGCCGGCT	*HIST1H1E* qPCR primer
	CCTTGGGTTTAACTGCTTTGGCCT	
MKRN1 guide RNAs	CACCGCAATGCACGACTAGAGAAGG	
	AAACCCTTCTCTAGTCGTGCATTG	
20A RNA oligonucleotide	GGAAAUAAACUGUAGAGUUCCAAAAAAAAAAAAAAAAAAAA	RNA Pulldown oligos
CAG RNA oligonucleotide	GGAAAUAAACUGUAGAGUUCCACGGAGCGACCCACCGAGCGC	

Table S6: siRNAs used in this study.

Name	Sequence 5' – 3'	Comment
MKRN1 siRNA1	CAGGCGAAGCUGAGUCAAGAA[dT][dT]	[7]
MKRN1 siRNA2	CGGGAUCCUCUCCAACUGCAA[dT][dT]	[8]
ZNF598 siRNA	CCCUCUAAAGUUGGGAAGA[dT][dT]	Sigma, Rosetta predictions
Control siRNA	UGGUUUACAUGUCGACUAA[dT][dT]	[9]

Supplemental References:

1. Kozlov G, Trempe JF, Khaleghpour K, Kahvejian A, Ekiel I, Gehring K. Structure and function of the C-terminal PABC domain of human poly(A)-binding protein. Proc Natl Acad Sci U S A. 2001;98(8):4409-13.

2. Pohlmann T, Baumann S, Haag C, Albrecht M, Feldbrügge M. A FYVE zinc finger domain protein specifically links mRNA transport to endosome trafficking. Elife. 2015;4.

3. Kozlov G, De Crescenzo G, Lim NS, Siddiqui N, Fantus D, Kahvejian A, et al. Structural basis of ligand recognition by PABC, a highly specific peptide-binding domain found in poly(A)-binding protein and a HECT ubiquitin ligase. EMBO J. 2004;23(2):272-81.

4. Ebersberger I, Simm S, Leisegang MS, Schmitzberger P, Mirus O, von Haeseler A, et al. The evolution of the ribosome biogenesis pathway from a yeast perspective. Nucleic Acids Res. 2014;42(3):1509-23.

5. Hildebrandt A, Alanis-Lobato G, Voigt A, Zarnack K, Andrade-Navarro MA, Beli P, et al. Interaction profiling of RNA-binding ubiquitin ligases reveals a link between posttranscriptional regulation and the ubiquitin system. Sci Rep. 2017;7(1):16582.

6. Cassar PA, Carpenedo RL, Samavarchi-Tehrani P, Olsen JB, Park CJ, Chang WY, et al. Integrative genomics positions MKRN1 as a novel ribonucleoprotein within the embryonic stem cell gene regulatory network. EMBO Rep. 2015;16(10):1334-57.

7. Ko A, Shin JY, Seo J, Lee KD, Lee EW, Lee MS, et al. Acceleration of gastric tumorigenesis through MKRN1-mediated posttranslational regulation of p14ARF. J Natl Cancer Inst. 2012;104(21):1660-72.

8. Kim JH, Park KW, Lee EW, Jang WS, Seo J, Shin S, et al. Suppression of PPARgamma through MKRN1-mediated ubiquitination and degradation prevents adipocyte differentiation. Cell Death Differ. 2014;21(4):594-603.

9. Heidelberger JB, Voigt A, Borisova ME, Petrosino G, Ruf S, Wagner SA, et al. Proteomic profiling of VCP substrates links VCP to K6-linked ubiquitylation and c-Myc function. EMBO Rep. 2018;19(4).
