# Supplementary material for: The RNA-binding ubiquitin ligase MKRN1 functions in ribosome-associated quality control of poly(A) translation
Source: Genome Biol. 2019 Oct 22;20:216. doi: 10.1186/s13059-019-1814-0 (PMC6805484; doi:10.1186/s13059-019-1814-0)
Supplement: Supplementary file 3 — Additional file 3: Figures S10-S12. with images of full membranes and different exposure times for Western blots and other analyses as specified. (PDF 2348 kb) [file 13059_2019_1814_MOESM3_ESM.pdf]

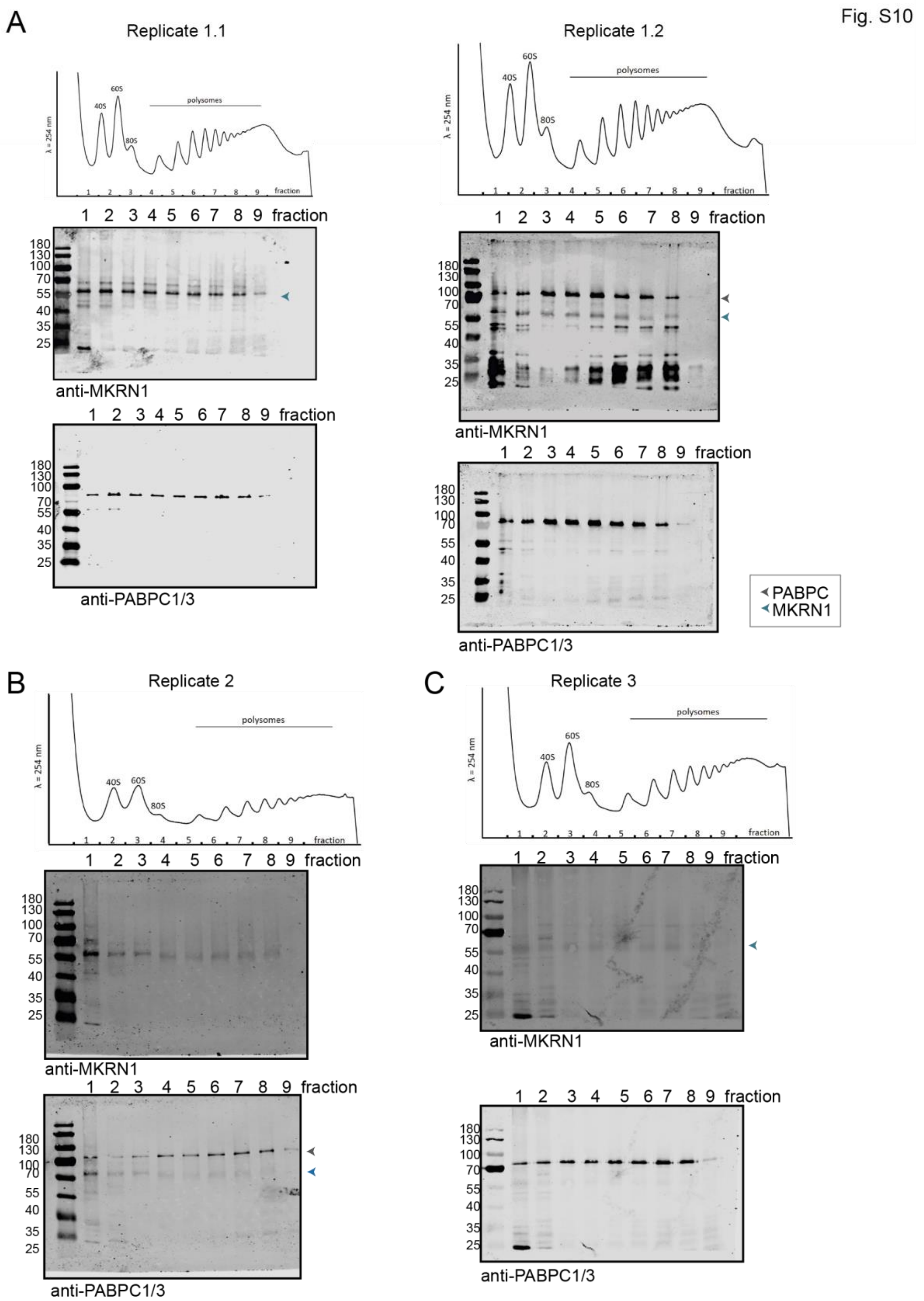

Fig. S10. Images of full membranes and different exposure times (exp.) for Western blot analyses in **Fig. 1B,D,F, Additional file 1: Fig. S1E**, and **S2C**. (*A*-*C*) Images of full membranes and replicate experiments for Western blot analyses of MKRN1 and PABPC1 in polysome profiling experiments in **Fig. 1B**. A 10%–50% sucrose gradient of cycloheximide-treated HEK293R cell extracts. Shown are the Western blot analyses of individual gradient fractions with antibodies against MKRN1 and PABPC1/3 (n = 3

replicates, plus one technical replicate). UV absorbance was measured at λ = 254 nm. (*A*) Left, Images of full membranes for MKRN1 and PABPC1/3 antibodies are shown for replicate 1 (replicate 1.1, as in **Fig. 1B**). Right, Full membranes for both antibodies are shown for a technical replicate of replicate 1.1 (replicate 1.2). (*B,C*) Images of full membranes for both antibodies are shown for replicates 2 (*B*) and 3 (*C*).

Fig. S10

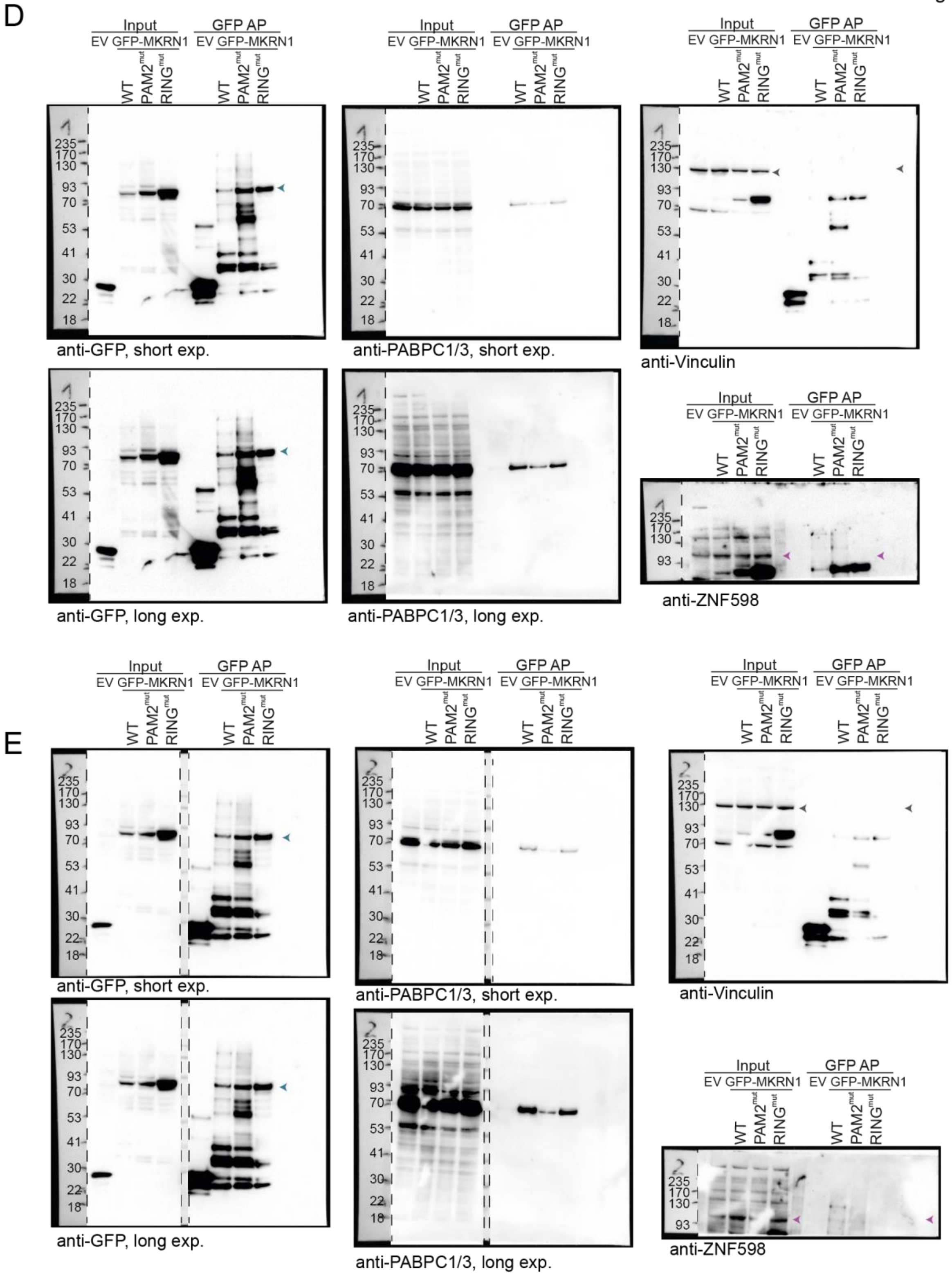

D
Input
GFP AP
EV GFP-MKRN1
WT
PAM2mut
RINGmut
235
170
130
93
70
53
41
30
22
18
anti-GFP, short exp.
anti-GFP, long exp.
anti-PABPC1/3, short exp.
anti-PABPC1/3, long exp.
anti-Vinculin
anti-ZNF598
E
anti-GFP, short exp.
anti-GFP, long exp.
anti-PABPC1/3, short exp.
anti-PABPC1/3, long exp.
anti-Vinculin
anti-ZNF598

Fig. S10

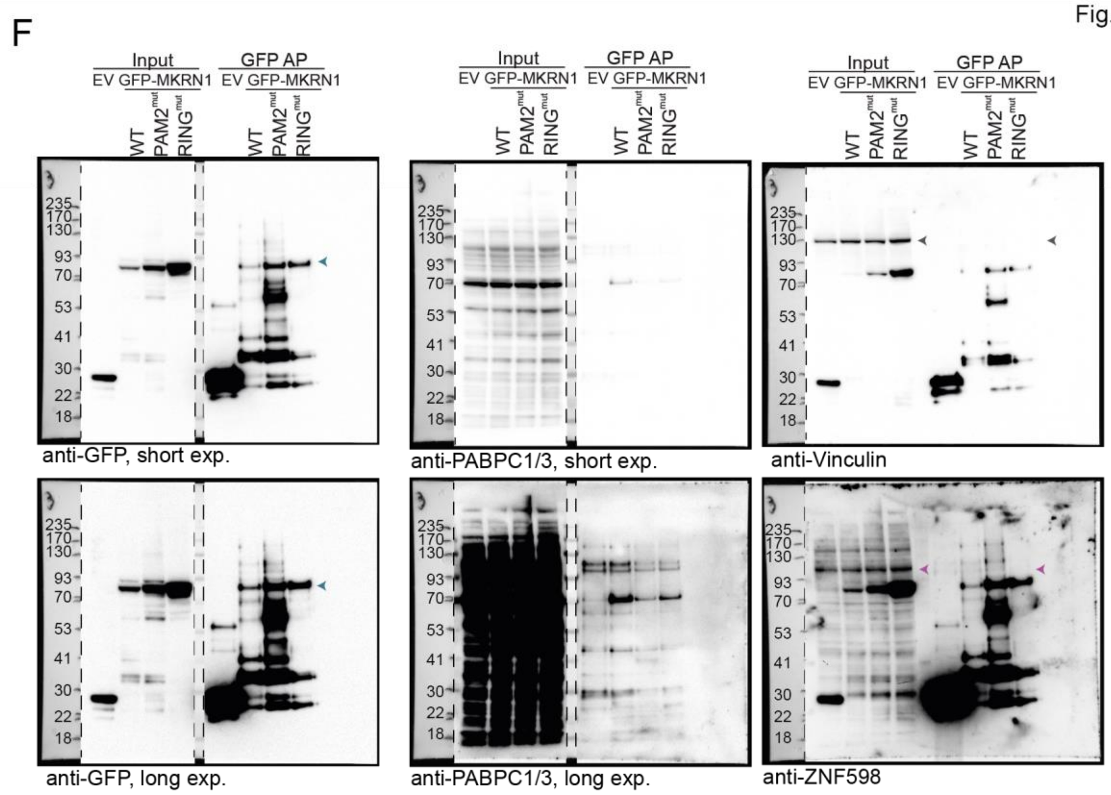

(*D-F*) Images of full membranes and different exposure times (exp.) for Western blot analyses in **Fig. 1D**. PABPC1 interacts strongly with MKRN1^{wt} and MKRN1^{RINGmut} but only to a lesser extend with MKRN1^{PAM2mut}. Western blot analysis was performed with antibodies against PABPC1/3 and GFP. Images of full membranes and different exposure (exp.) times for both antibodies are shown for replicate 1 (*D*), which is presented in **Fig. 1D**, as well as replicates 2 (*E*) and 3 (*F*). Petrol arrowheads indicate GFP-MKRN1. Grey and pink arrowheads indicate vinculin and ZNF598, respectively.

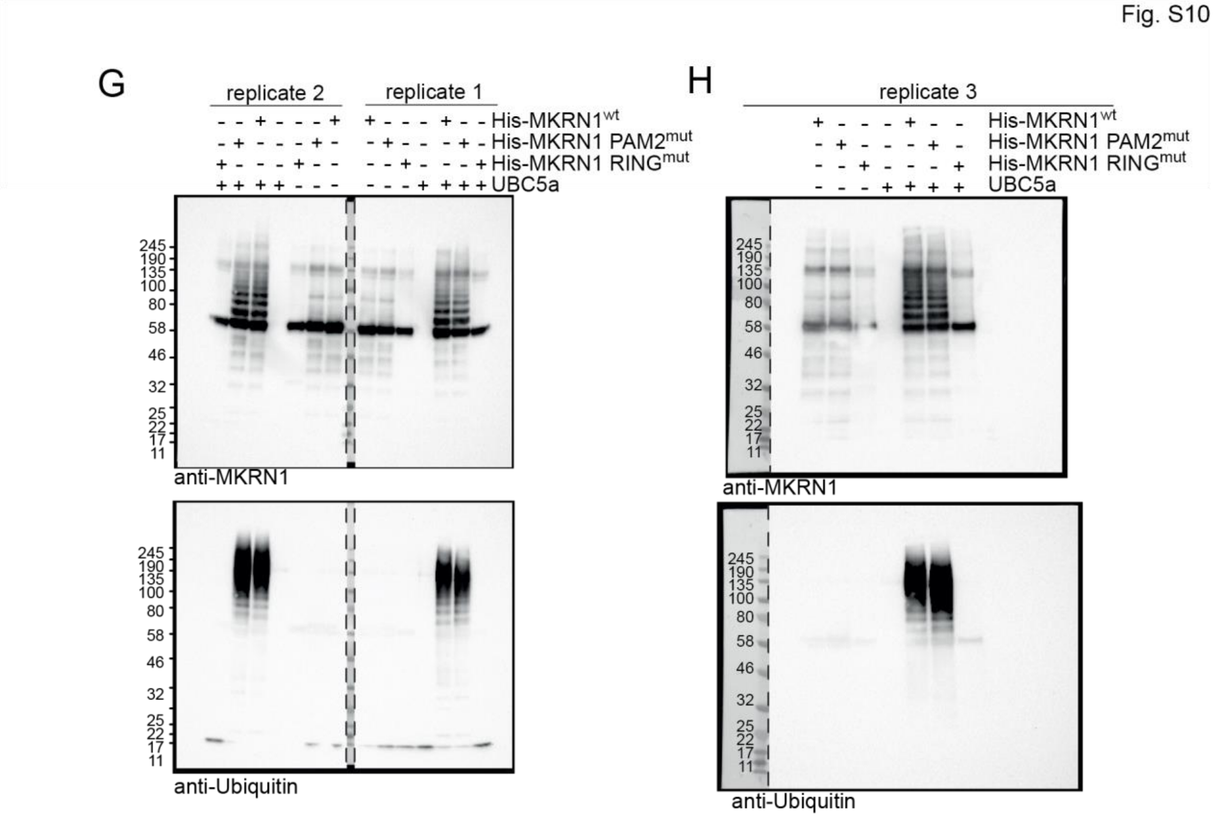

(*G,H*) Recombinant His-MKRN1^{wt} and His-MKRN1^{PAM2mut} are capable of autoubiquitylation opposed to His-MKRN1^{RINGmut}. Recombinant His-MKRN1^{wt}, His-MKRN1^{PAM2mut} and His-MKRN1^{RINGmut} proteins were incubated with or without the E2 enzyme UBC5a. UBC5a without an E3 enzyme was used as a control. Autoubiquitylation of MKRN1 was assessed by Western blot. Western blot analysis was performed with antibodies against MKRN1 and Ubiquitin. Images of full membranes for both antibodies are shown for replicate 1 (*G*, right), which is presented in **Fig. 1F**, and 2 (*G*, left), as well as for replicate 3 (*H*). Note the opposite order of replicates 1 and 2 (2 left, 1 right) in (*G*).

Fig. S10

I

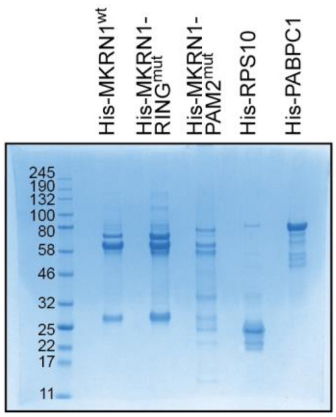

(*I*) Image of the full gel for recombinantly expressed proteins, which were purified from *E. coli*, as shown in **Additional file 1: Fig. S1E**.

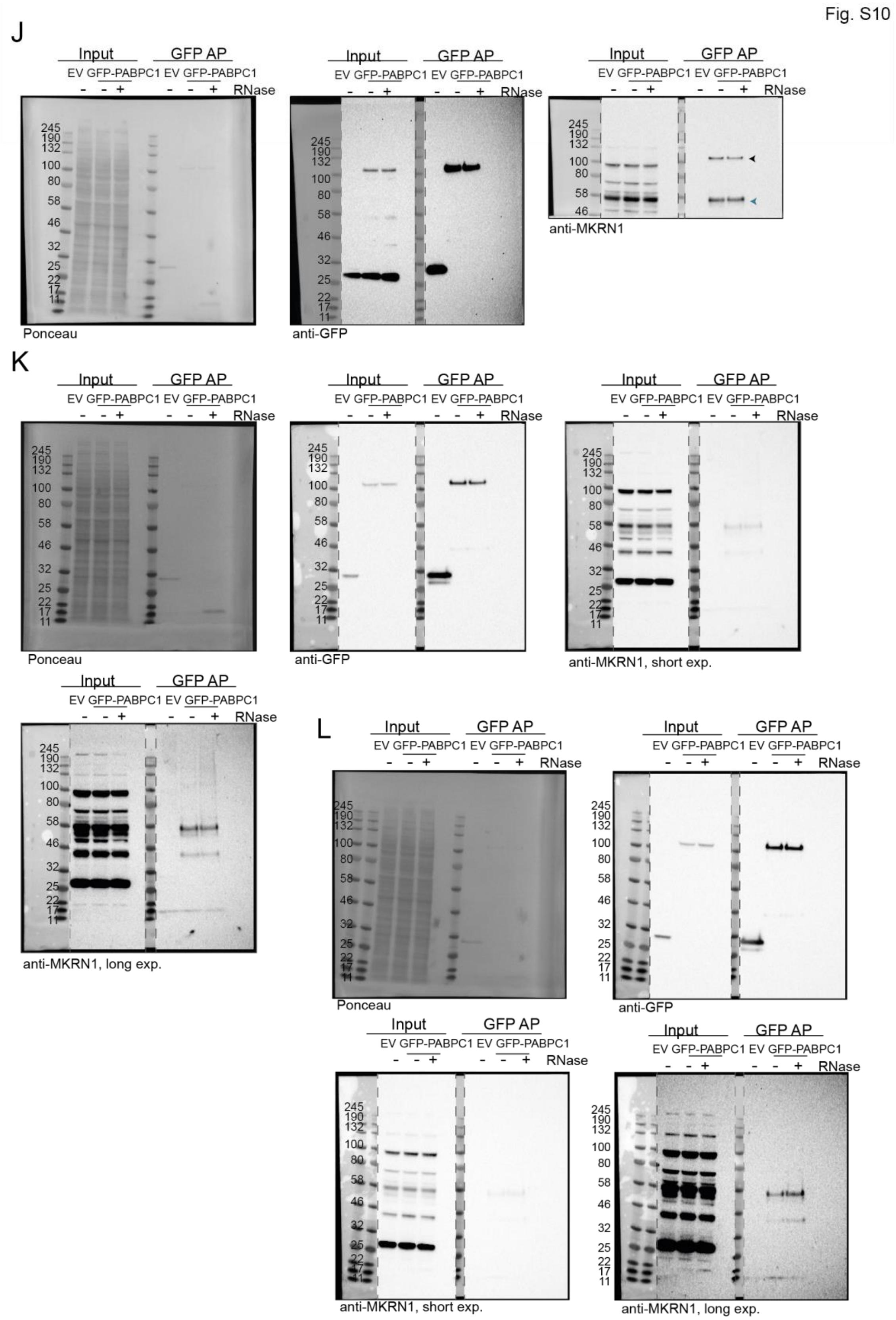

(*J-L*) Endogenous MKRN1 interacts with GFP-PABPC1 independent of RNA. Western blot analysis was performed with antibodies against MKRN1 and GFP. Images of full membranes and different exposure times for both antibodies are shown for replicate 1 (*J*), shown in **Additional file 1: Fig. S2C**, 2 (*K*) and 3 (*L*). Black and petrol arrowheads indicate GFP-PABPC1 and MKRN1, respectively.

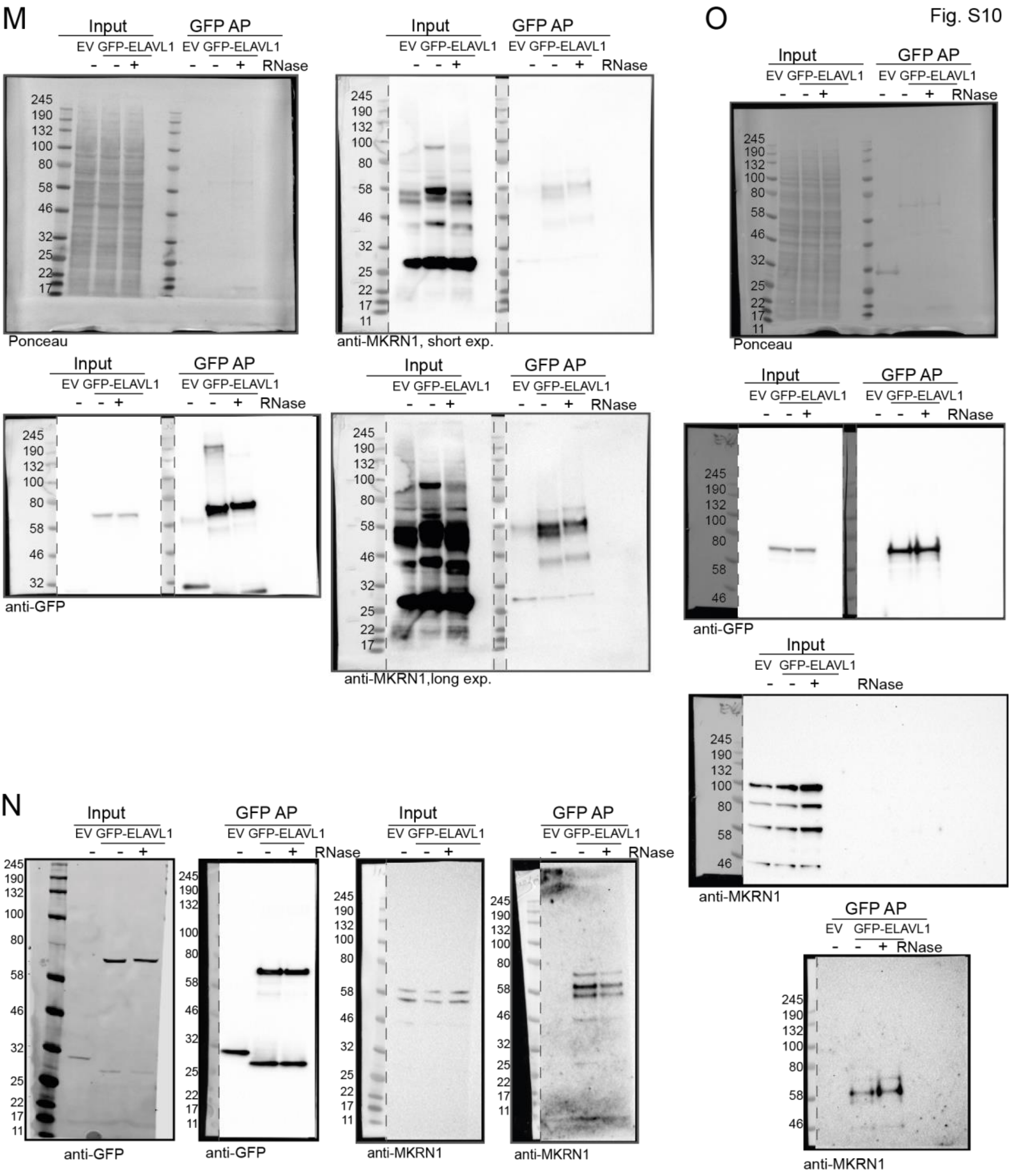

(*M*-*O*) Endogenous MKRN1 interacts with GFP-ELAVL1 independent of RNA. Western blot analysis was performed with antibodies against MKRN1 and GFP. Images of full membranes and different exposure times for both antibodies are shown for replicate 1 (*M*) which is presented in **Additional file 1: Fig. S2C**, as well as replicates 2 (*N*) and 3 (*O*).

Fig. S10

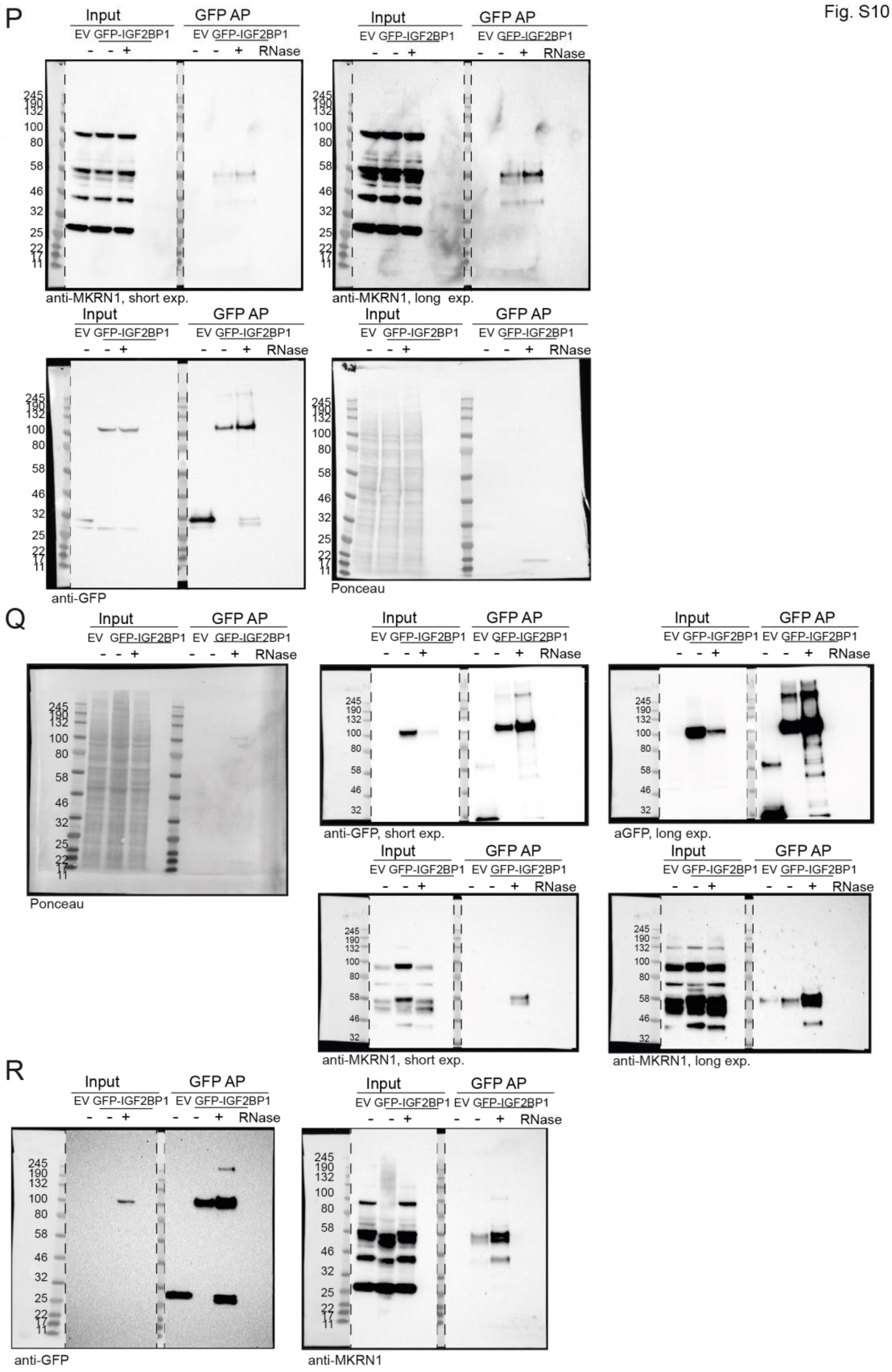

(*P-R*) Endogenous MKRN1 interacts with GFP-IGF2BP1 independent of RNA. Western blot analysis was performed with antibodies against MKRN1 and GFP. Images of full membranes and different exposure times for both antibodies are shown for replicate 1 (*P*) which is presented in **Additional file 1: Fig. S2C**, as well as replicates 2 (*Q*) and 3 (*R*).

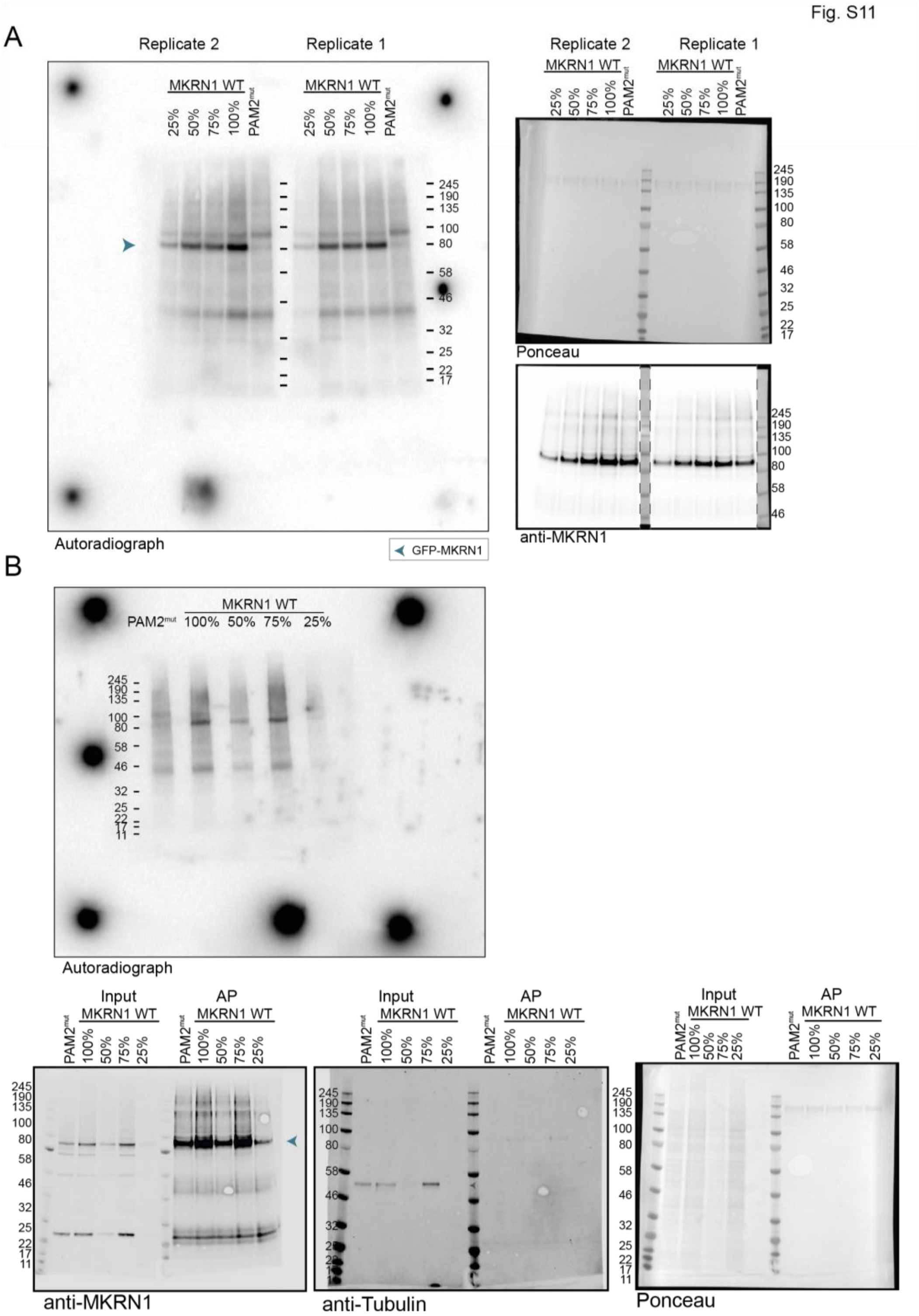

Fig. S11. Images of full membranes for Western blot and SDS-PAGE (Coomassie staining) analyses as in **Fig. 3D** and **Additional file 1: Fig. S6.** (*A,B*) Images of full membranes of autoradiographs and Western blot analyses in **Fig. 3D** (replicate 1) and **Additional file 1: Fig. S6A,B** (replicates 2 and 3). UV crosslinking experiments to measure the RNA binding capacity of GFP-MKRN1^{wt} and GFP-MKRN1^{PAM2mut}. Autoradiographs (*A*, left; *B*, top) and Western blots (*A*, right; *B*, bottom) show GFP-MKRN1/RNA complexes and GFP-MKRN1 protein, respectively, in the eluates from replicates 1 and 2 (with 4SU and UV crosslinking at 365 nm) (*A*) and 3 (with conventional UV crosslinking at 254 nm) (*B*). (*B*) Images of full membranes of Western

blot analyses with both antibodies are shown for replicate 3 (*B*). Petrol arrowheads indicate GFP-MKRN1-RNA complexes (*A*) or GFP-MKRN1 protein (*B*).

Fig. S11

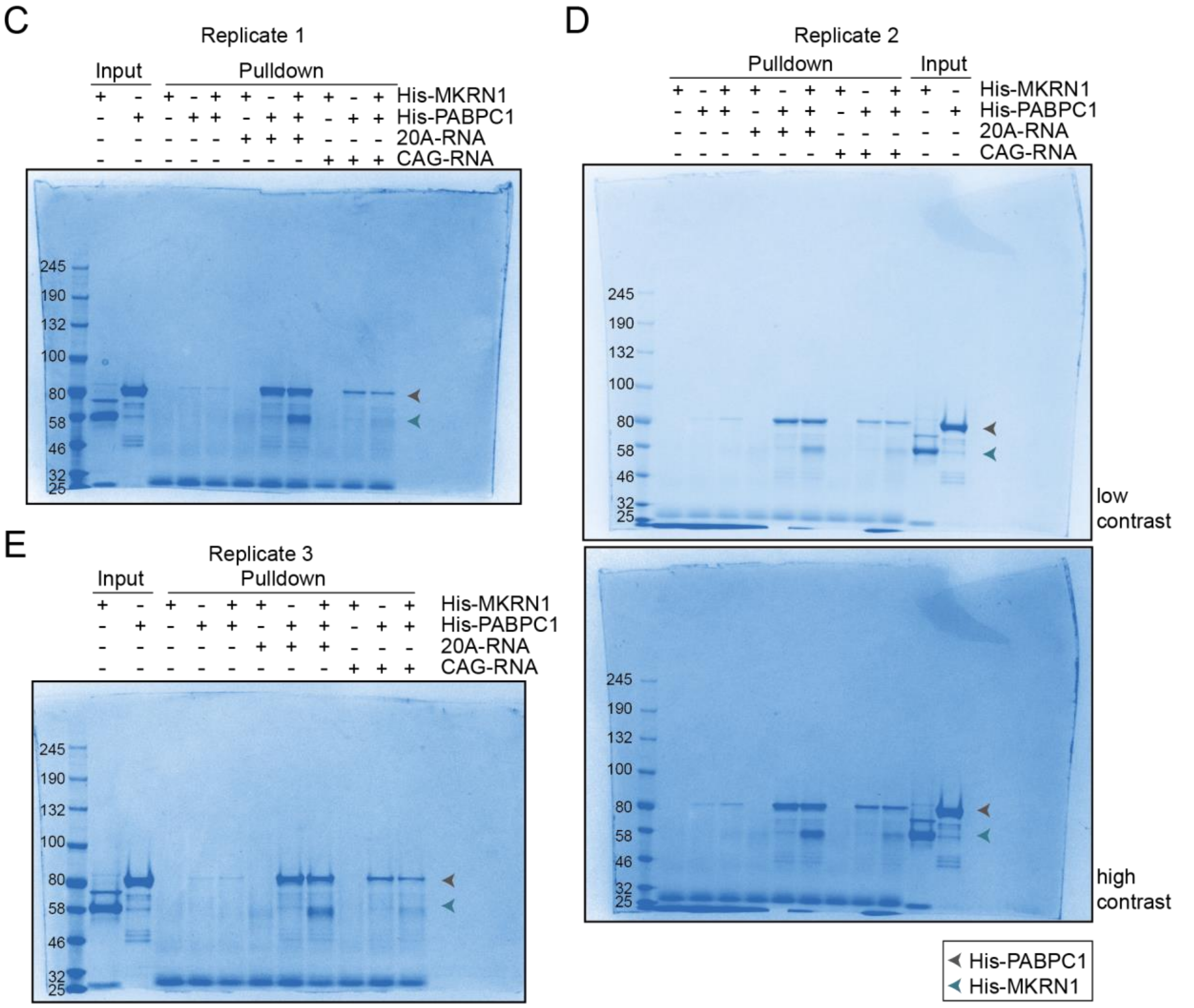

(*C*-*E*) Images of full gels of Coomassie SDS-PAGE analyses as presented in **Fig. 3E** (replicate 1; *C*), and **Additional file 1: Fig. S6C** (replicate 2; *D*) and **Additional file 1: Fig. S6D** (replicate 3, *E*) are shown. Petrol and grey arrowheads indicate His-MKRN1 and His-PABPC1, respectively.

Fig. S12

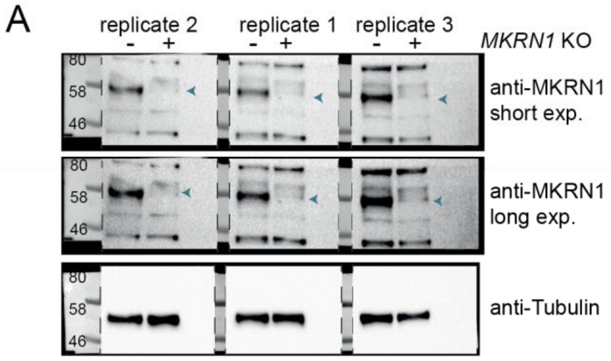

Fig. S12. Images of full membranes for Western blot analyses in **Additional file 1: Fig. S8A,C,E,F, Fig. 5G**, and **Additional file 1: Fig. 9B**. (*A*) Images of full membranes for Western blot analyses in **Additional file 1: Fig. S8C**. Images of full membranes are presented for replicates 1, 2 and 3. MKRN1 protein levels were assessed by Western blot in *MKRN1* KO HEK293T (*MKRN1* KO) and HEK293T wild type (WT) cells. Western blot analysis was performed with antibodies against MKRN1 and tubulin. Petrol arrowheads indicate MKRN1.

Fig. S12

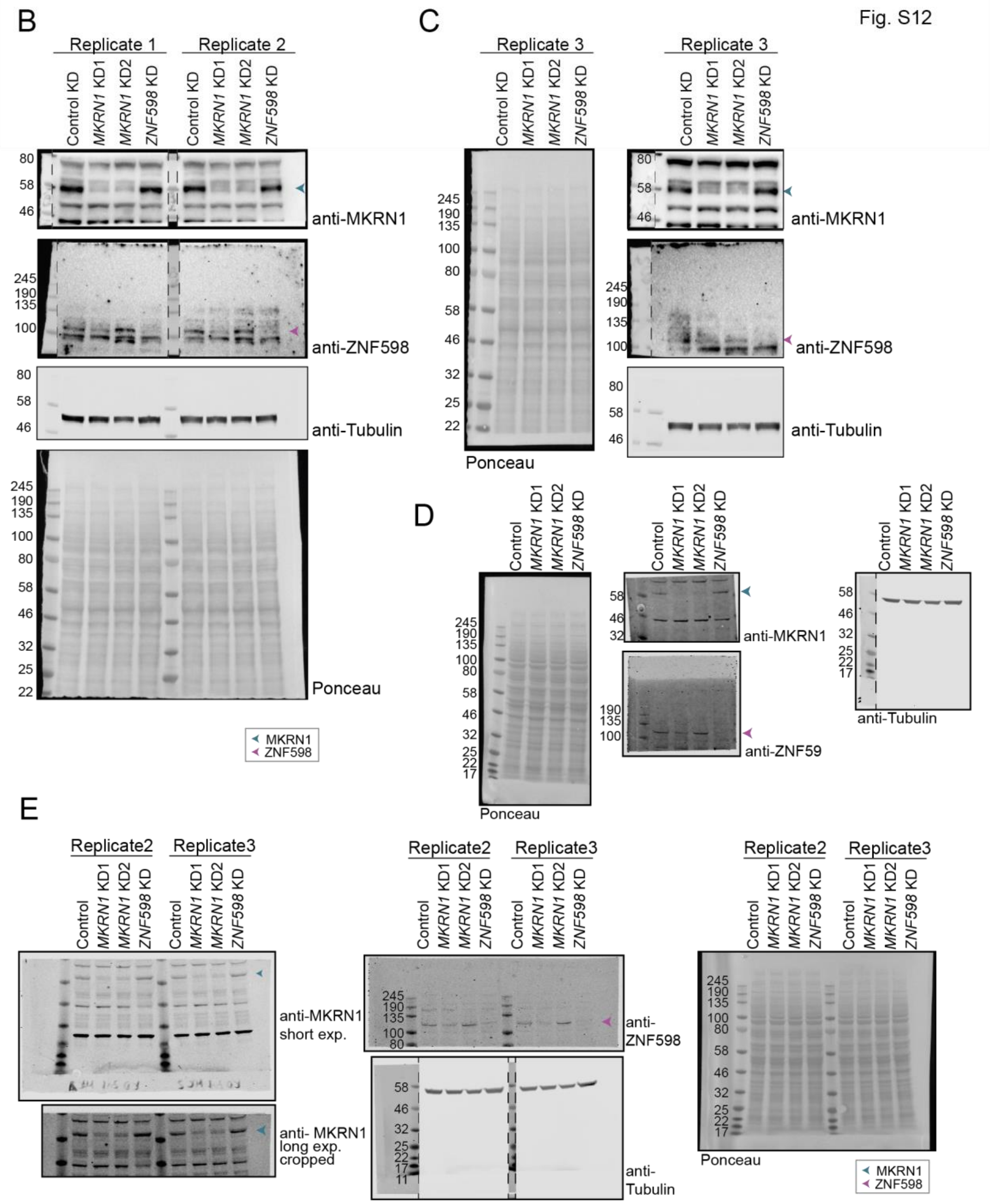
B
C
Replicate 1
Replicate 2
Replicate 3
Control KD
MKRN1 KD1
MKRN1 KD2
ZNF598 KD
anti-MKRN1
anti-ZNF598
anti-Tubulin
Ponceau
MKRN1
ZNF598
D
Control
anti-ZNF59
E
Replicate2
Replicate3
anti-MKRN1 short exp.
anti- MKRN1 long exp. cropped
anti- ZNF598
anti- Tubulin
80
58
46
245
190
135
100
32
25
22
17
11

Fig. S12

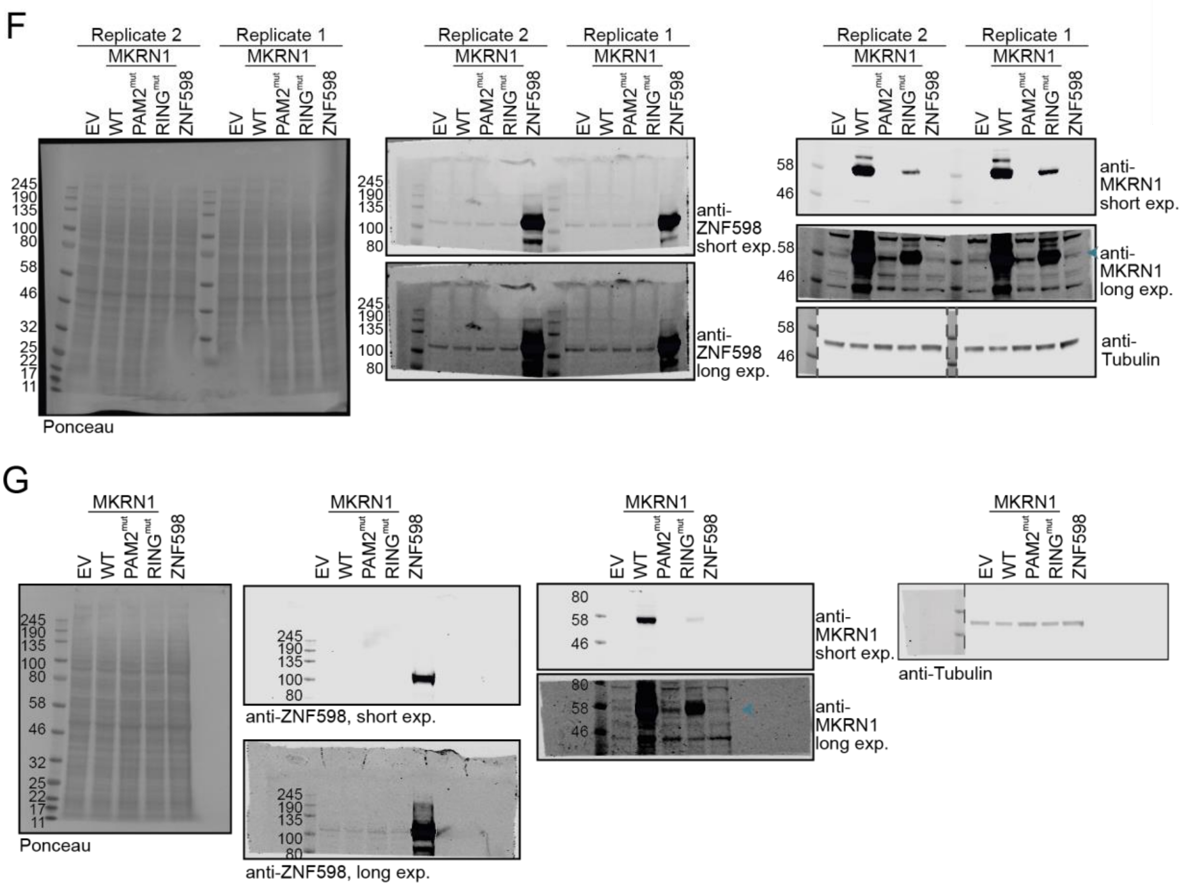

(*B-G*) Images of full membranes and different exposure (exp.) times for Western blot analyses in **Additional file 1: Fig. S8A,E,F.** (*B,C*) KDs of *MKRN1* and *ZNF598* assessed by Western blot (n=3 replicates) from **Additional file 1: Fig. S8A**. Western blot analysis was performed with antibodies against MKRN1, ZNF598, and tubulin. Petrol and pink arrowheads indicate MKRN1 (53 kDa) and ZNF598 (99 kDa), respectively. Uncropped gel images of replicates 1 and 2 (*B*) and 3 (*C*). (*D,E*) Images of full membranes are shown for cross-regulation between *MKRN1* and *ZNF598* KD from **Additional file 1: Fig. S8E**. *MKRN1* KD1 reduces endogenous ZNF598 protein levels. Western blot analysis was performed with antibodies against MKRN1, ZNF598, and tubulin. Coloured arrowheads as in (*B*). Uncropped gel images of replicate 1 (*D*) and replicates 2 and 3 (*E*). (*F,G*) Images of full membranes are shown for cross-regulation of *MKRN1* and *ZNF598* overexpression (OE) from **Additional file 1: Fig. S8F**. *ZNF598* OE reduces MKRN1 protein levels. Western blot analysis was performed with antibodies against MKRN1, ZNF598, and tubulin. Black arrowheads indicate MKRN1. Images of full membranes and different exposure times (exp.) for both antibodies are shown for replicates 1, 2 (*F*), and 3 (*G*). Note the opposite order of replicates 1 and 2 (2 left, 1 right) in (*F*). Coloured arrowheads as in (*B*).

Fig. S12

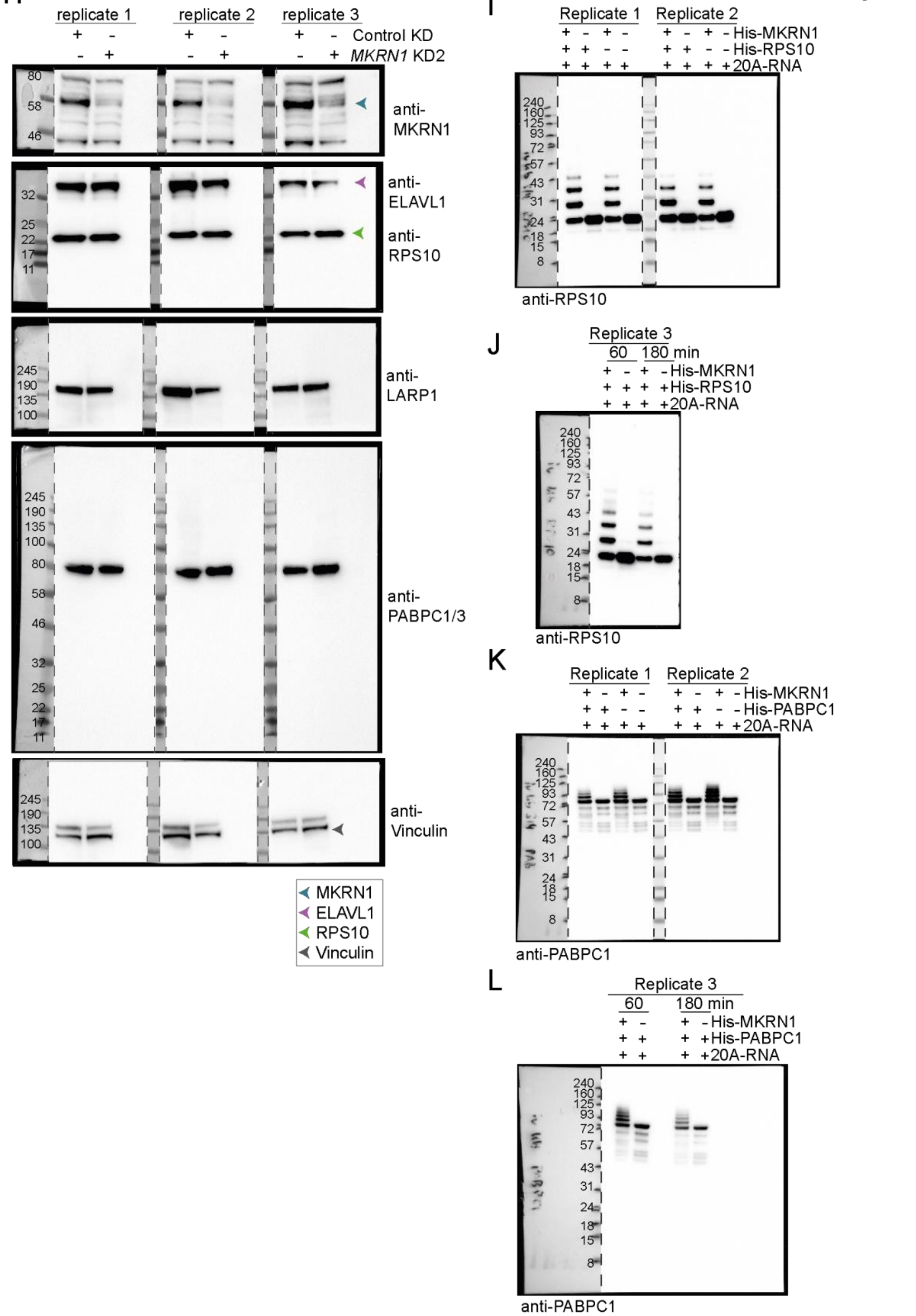

(*H*) Images of full membranes for Western blot analyses for replicate 1, as presented in **Additional file 1: Fig. S9B**, replicate 2, and 3 are shown. Western blot analyses were performed with antibodies against MKRN1 (petrol arrowhead), ELAVL1 (pink

arrowhead), RPS10 (green arrowhead), LARP1, PABPC1/3, and vinculin (grey arrowhead). (*I,J*) His-RPS10 was incubated with or without His-MKRN1 and with or without A_{20} RNA oligonucleotides. Images of full membranes for Western blot analyses for replicate 1, as presented in **Fig. 5G** (left), replicate 2 (both *I*) and replicate 3 (*J*) are shown. Western blot analysis was performed with an antibody against RPS10. (*K,L*) His-PABPC1 was incubated with or without His-MKRN1 and with or without A_{20} RNA oligonucleotides. Images of full membranes for Western blot analyses for replicate 1, as presented in **Fig. 5G** (right), replicate 2 (both *K*), and 3 (*J*) are shown. Western blot analysis was performed with antibody against PABPC1/3.
