# Supplementary material for: The RNA-binding ubiquitin ligase MKRN1 functions in ribosome-associated quality control of poly(A) translation
Source: Genome Biol. 2019 Oct 22;20:216. doi: 10.1186/s13059-019-1814-0 (PMC6805484; doi:10.1186/s13059-019-1814-0)
Supplement: Supplementary file 6 — Additional file 6: Review history. (DOCX 34 kb) [file 13059_2019_1814_MOESM6_ESM.docx]

Review History

**First round of review**

**Reviewer 1**

**Are you able to assess all statistics in the manuscript, including the appropriateness of statistical tests used?** Yes, and I have assessed the statistics in my report.

**Comments to author:**

The authors report that the RNA-binding ubiquitin ligase MKRN1 functions in RQC. Interaction with PABP is shown to be necessary for the RNA binding and activity in RQC of MKRN1, which is also reported to ubiquitinate RPS10. This results in ribosome stalling prior to entry into an A-tract. The authors suggest this leads to further collisions that are sensed by ZNF598 and resolved by the RQC machinery.

The work is of good quality and the findings are novel and will be of wide interest. Some points should be addressed prior to publication.

Major:
1: To detect direct ubiquitination by MKRN1, it seems to be more appropriate to compare ubiquitin remnant profiles of MKRN1 wt and RING-mut rather than MKRN1 wt and MKRN1 KD cells, as in the KD, any indirect effects could also play a role. Can the authors at least show for RPS10 that it is no longer ubiquitinated in MKRN1 RING-mut?

Minor:
2: The authors report strong binding of MKRN1 upstream of pA sites. It was very recently reported that the ZNF598 orthologue in yeast, Hel2, shows a similar binding pattern (Winz et al., Nat. Comm, 2019), but this was not observed for ZNF598 (Garzia et al., Nat. Comm. 2017). This suggests the possibility that the roles of yeast Hel2 have in some sense been split between human MKRN1 and ZNF598.

3: P13: The authors propose that MKRN1 acts to stabilize PABP on shorter A stretches. It would be useful to briefly explain how the authors envisage this to work.

4: Abbreviation list; SIALC => SILAC 

5: Part of the Discussion reads like a replication of the Results.

6: Fig. 1 A & D. It would be helpful to add a table presenting the underlying data, at least for the significant proteins, as many of the dots are not labelled in the plot. This could be mentioned in the figure caption. 
The ribosomal proteins are difficult to distinguish from other proteins. Consider better optical distinction.

7: Figs. 5E and 5F. The colours are difficult to distinguish in the PDF.

8: Fig. 6; Consider placing "premature" in parentheses, as in the figure caption, as data suggest that there could be activity also at canonical poly(A) sites.

9: Fig. S2B mentions NMD as top biological process, but there is no discussion of this in the paper. This could be interesting and the authors might usefully comment.

10: Fig. S3 seems to lack a colour code.

11: Fig S4D. Is this the same as the main text figure? If so, it should probably be mentioned.

12: Line 43; extra "the"

13: Line 140; 4,6 => 4.6

14: Line 166: By "increased A-content" do the authors intend "terminal, non-templated A tracts"? If so, something like this would be clearer.

**Reviewer 2**

**Are you able to assess all statistics in the manuscript, including the appropriateness of statistical tests used?** No, I do not feel adequately qualified to assess the statistics.

**Comments to author:**

In this manuscript, Hildebrandt and colleagues analyse the RNA binding profile of the E3 ubiquitin ligase MKRN1. They find that MKRN1 binds upstream of A-stretches, that RNA binding is lost when MKRN1 cannot bind to PABP, that MKRN1 ubiquitylates RPS10 and other RBPs related with translational control, and that MKRN1 is involved in RQC using reporter assays. The experiments are well-performed and the data support the proposed model for MKRN1 function. However, there are some over-statements. In addition, further controls will help support the central argument of this manuscript:

1) It is unclear why, in the first place, the authors focused on MKRN1. Can the authors explain this in the introduction? 

2) The authors state that MKRN1 "marks" the beginning of poly(A) tails. However, this could be expected from many RBPs, as many RBPs bind to the 3' UTR while very few bind to the poly(A) tail. How does MKRN1 distribution compare with that of other 3' UTR binding proteins?

3) The authors state that "MKRN1 is positioned upstream of poly(A) sequences through direct interaction with PABP" (page 12, lanes 243-244). The strongest evidence for this is that the MKRN1-PAM2 mutant, which loses its interaction with PABP, also loses RNA binding. But it could also be that the structure of the PAM2 mutant is distorted so that it cannot bind RNA anymore. This is supported by the low levels of this mutant when over-expressed in cells (see also comment 4). Can the authors provide evidence that the MKRN1-PAM2 mutant is functional in other assays (e.g. ubiquitylation), and therefore is properly folded? 

4) Figure 1C: The PAM2 mutant is barely expressed, and this might be the reason why no signal is detected in the IP. A new experiment with reasonable expression of this mutant should be shown. Similarly, the "slight increase in PABPC1 binding" alluded to in page 7 (lanes 128-129) for the RING mutant could be due to the much higher expression of this protein and not to stabilization. Given the large differences in expression, a simple correction to input might be misleading in this case.

5) Related with comment 3, the fact that A-stretches are close to MRNK1 sites does not mean that PABP and MKRN1 bind together to the mRNA, as stated along the manuscript. In addition, in the discussion the authors hypothesize a mutually dependent (cooperative?) binding of PABP and MKRN1 to short A-stretches. Gel mobility shift assays should help reinforce the author's hypothesis and will be instrumental for the conclusions.

6) There are no changes in the levels of RPS10, PABPC1/C4, IGFBP1/2/3, ELAVL1 and MOV10 upon MKRN1 depletion (Sup Fig S8B). Why does a defect in ubiquitylation of these substrates not reflect at the total protein level? Why would these substrates show increased interaction with the MKRN1-RINGmut (Fig S7B) if their increased presence cannot be attributed to increased levels? 

7) Can the authors show a Venn diagram depicting the degree of overlap of peaks and genes identified in the 3 MKRN1 iCLIP replicates?

8) Please, explain the difference between Figure 2D and S4D. Note that there are several mistakes in the legend of Fig S4 (panel descriptions swapped or missed).

9) Fig 3D: Please, indicate that the bottom panel is a western-blot.

10) Page 9, lanes 164-166: It would be more correct to state "In accordance with our hypothesis that MKRN1 binds upstream of the poly(A) tail…".

11) Page 9, lanes 158-160: Eight adenosines is not the RNA footprint of PABP, but of one RRM of PABP. Please, correct.

12) Page 10 (lane 205): That the effects of MKRN1 and ZNF598 are not additive rather means that they work in the same pathway, reinforcing the model proposed by the authors.

13) Page 10 and Figure 4: Please, explain the stalling reporter as well as the principle of the assay in detail. One should not have to go to the indicated reference to understand it. 

14) Page 11, lane 228: Please, clarify in the main text that the interactions reported in this manuscript have been included. 

15) Page 13, lane 267: This title should be tuned down by removing "to stall ribosomes" or by adding the word "model".

16) The word "indicate" should be substituted by "suggest" in all instances where the author's conclusions are just inferences and no direct experimental data are provided.

**Reviewer 3**

**Are you able to assess all statistics in the manuscript, including the appropriateness of statistical tests used?** No, I do not feel adequately qualified to assess the statistics.

**Comments to author:**

The manuscript by Hildebrandt and colleagues explores the role of the E3 ligase MKRN1 in ribosome-quality control. Prior work on the protein implicated it in a multitude of processes including control of telomere length, RNA polymerase II, cell cycle and regulation of p53 levels. The authors in this paper focus on one particular aspect of MKRN1's function in regulating translation through its interaction with polyA-binding protein (PABP). In the first set of experiments, they confirmed this interaction with mass-spec analysis as well as co-immunoprecipitation, and identified a PAM2 motif in MKRN1 responsible for interacting with PABP. In the second set of experiments, the authors went on to show that this interaction between MKRN1 and PABP could recruit the factor to polyA stretches in coding sequences. These first two set of experiments by and large served as confirmation for what others have reported in previous papers. Arguably, the most interesting pieces of observations came from the third set of experiments, during which the authors showed a potential role for the factor in promoting ribosome stalling on polyA sequences and that its depletion results in reduction of ribosomal protein ubiquitination. Recent data have revealed that ubiquitination of ribosomal proteins of the small subunit by ZNF598 is important for ribosome rescue, RNA cleavage and degradation of the nascent peptide. The authors claim that MKRN1 acts upstream of ZNF598 to prevent the ribosome from translating polyA sequences by adding ubiquitin chains to the leading ribosome.

While some of the observations made by the authors were interesting and could add to our understanding of ribosome-quality control, I felt that the authors made a huge leap coming up with their final model with the limited data presented. Many of the ideas were underdeveloped and the authors appear to have overinterpreted their data. For instance, the authors claim that MKRN1 is responsible for ubiquitinating ribosomal proteins based on the observation that ubiquitination is reduced when the protein is depleted. An alternative model is that the protein might be responsible for recruiting ZNF598 or another E3 ligase. Why the authors did not try to rescue this effect with different MKRN1 constructs is unclear. Furthermore, why the authors used siRNAs instead of CRISPR was not obvious. The authors should also consider carrying out in vitro ubiquitination assays to show that MKRN1 is capable of adding ubiquitin chains to uS10. This is standard in the field, and earlier studies on ZNF598 used these sort of analyses to assign a function for the protein. The authors should also consider validating some of their mass spec analysis using western blotting on in vivo targets. Overall, while the study has the potential of adding to the emerging field of ribosome ubiquitination and mRNA-quality control, in its current form the paper falls short of being appropriate for Genome Biology

Other issues:
1)     The authors provided data that showed depletion of MKRN1 by siRNAs leads to an increase in translation of a polyA-containing reporter and claim that this derepression is similar to what others observed for ZNF598 knockdown. Others have reported almost complete derepression, which is not the case for MKRN1 knockdown, which shows 2 to 3 fold derepression at best. 
2)     Is MKRN1 associated with polysomes? This is important to assess since PABP is.
3)     qPCR or northern analysis of the stalling reporter should be conducted to assess the effect of the factor on no-go decay.
4)     Figure 1C, the claim that PAM2mut does not interact with PABP is not convincing since that particular construct is hardly expressed. The quantification in panel D does not appear to show significant difference between the wild type and the mutant.

**Authors Response**

**Point-by-point responses to the reviewers’ comments:**

Reviewer #1:

The authors report that the RNA-binding ubiquitin ligase MKRN1 functions in RQC. Interaction with PABP is shown to be necessary for the RNA binding and activity in RQC of MKRN1, which is also reported to ubiquitinate RPS10. This results in ribosome stalling prior to entry into an A-tract. The authors suggest this leads to further collisions that are sensed by ZNF598 and resolved by the RQC machinery.

The work is of good quality and the findings are novel and will be of wide interest. Some points should be addressed prior to publication.

Major:
1: To detect direct ubiquitination by MKRN1, it seems to be more appropriate to compare ubiquitin remnant profiles of MKRN1 wt and RING-mut rather than MKRN1 wt and MKRN1 KD cells, as in the KD, any indirect effects could also play a role. Can the authors at least show for RPS10 that it is no longer ubiquitinated in MKRN1 RING-mut?

*In order to demonstrate that MKRN1 can directly modify RPS10 and PABPC1, we have now performed in vitro ubiquitylation assays with recombinant MKRN1 purified from E. coli. We show that recombinant MKRN1wt can efficiently ubiquitylate RPS10 and PABPC1, both identified as putative substrates of MKRN1 by ubiquitin remnant profiling. 

The in vitro ubiquitylation data is presented in the new Figure 5G. 

In addition, we analysed the impact of MKRN1RINGmut on ribosome stalling. This was done by complementing the MKRN1 KD cells with stable integration of MKRN1wt, MKRN1PAMmut or MKRN1RINGmut constructs. Only MRKN1wt was capable of partially reverting the KD effect, such that ribosome stalling was restored, whereas neither of MKRN1 mutants was functional in this assay.* 
 *The complementation is shown in the new Figure 4D.*

Minor:
2: The authors report strong binding of MKRN1 upstream of pA sites. It was very recently reported that the ZNF598 orthologue in yeast, Hel2, shows a similar binding pattern (Winz et al., Nat. Comm, 2019), but this was not observed for ZNF598 (Garzia et al., Nat. Comm. 2017). This suggests the possibility that the roles of yeast Hel2 have in some sense been split between human MKRN1 and ZNF598.

*We thank the Reviewer for pointing this out. We have added the following text in the discussion of the revised manuscript: 
“However, a recent study in yeast has shown that Hel2p binds before and also after the stop codon within 3’ UTRs of mRNAs (Winz et al. 2019) while such a binding pattern has not been observed for ZNF598 in human cells (Garzia et al. 2017). Even though MKRN1 and Hel2p binding patterns are not identical, this suggests the possibility that the roles of yeast Hel2p might have been split between MKRN1 and ZNF598 in human cells.”*

3: P13: The authors propose that MKRN1 acts to stabilize PABP on shorter A stretches. It would be useful to briefly explain how the authors envisage this to work.

*In order to address the conjoint binding of MKRN1 and PABP, we have now performed in vitro RNA pulldown assays, in which we incubated recombinant PABPC1 and/or MKRN1 with bead-coupled RNAs with or without a poly(A) stretch. We could show that PABPC1 strongly increased the pulldown of MKRN1 with the A20-harbouring RNA but not the control RNA. This result supports the model that PABPC1 stabilises MKRN1 at longer A-stretches. 

The RNA pulldown experiment is shown in the new Figure 3E. 

Further experiments would need to be performed to investigate the effects of MKRN1 on PABPC1 binding to shorter A-stretches. We have therefore removed the respective sentences from the revised manuscript.*

4: Abbreviation list; SIALC => SILAC

*This was corrected.*

5: Part of the Discussion reads like a replication of the Results.

*We have modified the discussion to avoid the repetition of the results.* 

6: Fig. 1 A & D. It would be helpful to add a table presenting the underlying data, at least for the significant proteins, as many of the dots are not labelled in the plot. This could be mentioned in the figure caption. 
The ribosomal proteins are difficult to distinguish from other proteins. Consider better optical distinction.
*We now provide the complete list of quantified protein groups in the MKRN1 SILAC APs shown in Figure 1A and 1D (Figure 1A,E in the revised manuscript) as Supplemental Table S1 (Excel). 

The table include the MaxQuant output and results of limma analysis that was used to identify significantly enriched proteins from the AP experiments. The significantly enriched proteins are colour-coded.*

*We have changed the colour scheme in Figure 1A,D (Figure 1A,E in the revised manuscript).*

7: Figs. 5E and 5F. The colours are difficult to distinguish in the PDF.

*We have changed the colour scheme in Figure 5E,F.* 

8: Fig. 6; Consider placing "premature" in parentheses, as in the figure caption, as data suggest that there could be activity also at canonical poly(A) sites.

*This has been done in the revised manuscript.* 

9: Fig. S2B mentions NMD as top biological process, but there is no discussion of this in the paper. This could be interesting and the authors might usefully comment.

*We thank the Reviewer for pointing this out. We now mention this finding in the Results section: “Nonsense-mediated decay” and “translation” were the most significantly enriched Gene Ontology (GO) terms for the MKRN1 interaction partners”. We decided to not include it in the discussion, since we did not follow it up and it would be very speculative.* 

10: Fig. S3 seems to lack a colour code.

*The colour codes were added in the revised figure (now Supplemental Figure 4).* 

11: Fig S4D. Is this the same as the main text figure? If so, it should probably be mentioned.

*Both figures address the link between MKRN1 binding strength and downstream poly(A) sequences. In order to capture the RNA binding preference of MKRN1, we used two different metrics to describe the enrichment of A’s downstream of the MKRN1 binding sites: 

(i) We defined “A-rich stretches” (referred to as “A-stretches”) as windows up to 30-nt which show at least 70% of A nucleotides (for details, see Methods on page 28f). These A-stretches were used in most analyses, including Supplemental Figure S4D (now Supplemental Figure S5D) which shows that MKRN1 binding site strength steadily increases with A-stretch length (“longer A-stretches associated with stronger MKRN1 binding”, page 9, line 283f). 

(ii) We complemented the A-stretch analysis with a more stringent search for continuous stretches of uninterrupted A’s (referred to as “A-runs”) within the A-stretches. Figure 2D shows that MKRN1 binding site strength increases up to 8 A’s and then plateaus. This A8 preference nicely matched the RNA footprint of one RRM domain of PABP, as recently determined by the Passmore lab (Webster et al, 2018). 

In order to make the results clearer, we revised the terminology. We now use “A-rich stretch” and “continuous A’s” throughout text and figures.* 

12: Line 43; extra "the"
13: Line 140; 4,6 => 4.6

*This was corrected.* 

14: Line 166: By "increased A-content" do the authors intend "terminal, non-templated A tracts"? If so, something like this would be clearer.

*In the initial analysis in Figure 3B, we determined the fraction of A nucleotides in the unmapped reads, irrespective of their position in the read. Following the Reviewer’s suggestion, we now complemented this analysis by explicitly counting the number of continuous A’s from the 3’ end of the reads. The elevated number of A’s at read 3’ ends in the MKRN1 data supports the assumption that reads with increased A-content derive from poly(A) tails. 

We included the new analysis as a new inset in Figure 3B.* 

Reviewer #2: 

In this manuscript, Hildebrandt and colleagues analyse the RNA binding profile of the E3 ubiquitin ligase MKRN1. They find that MKRN1 binds upstream of A-stretches, that RNA binding is lost when MKRN1 cannot bind to PABP, that MKRN1 ubiquitylates RPS10 and other RBPs related with translational control, and that MKRN1 is involved in RQC using reporter assays. 

The experiments are well-performed and the data support the proposed model for MKRN1 function. However, there are some over-statements. In addition, further controls will help support the central argument of this manuscript: 

1) It is unclear why, in the first place, the authors focused on MKRN1. Can the authors explain this in the introduction? 

*We initially started to work on MKRN1 because of its interesting domain structure combining RNA-binding domains with a RING E3 ubiquitin ligase function. Based on its interaction partners and its particular binding pattern upstream of poly(A) sequences, we hypothesised that it could be involved in RQC. 

We added a following sentence in the introduction to clarify this: “The presence of several RNA binding domains and a RING domain in MKRN1 prompted us to study the function of MKRN1 in human cells.”*

2) The authors state that MKRN1 "marks" the beginning of poly(A) tails. However, this could be expected from many RBPs, as many RBPs bind to the 3' UTR while very few bind to the poly(A) tail. How does MKRN1 distribution compare with that of other 3' UTR binding proteins?

*Following the Reviewer’s suggestion, we tested how MKRN1 binding compares to other RBPs in the 3’ UTR. To this end, we used publicly available eCLIP data sets from ENCODE for several RBPs. Specifically, we chose TIAL1, PUM1, QKI, UPF1 and HNRNPK, which are all known to fulfil different functions in the 3’ UTR, as well as PABPC4 and CPSF6, a component of the cleavage and polyadenylation machinery, which are expected to bind near/at poly(A) tails. Whereas TIAL1, PUM1, QKI, UPF1 and HNRNPK binding was distributed throughout 3’ UTR bodies, PABPC4 and CPSF6 peaked together with MKRN1 towards the polyadenylation sites. This supports the notion that the observed binding pattern of MKRN1 is not just a result of general 3’ UTR binding, but reflects a specific enrichment near poly(A) sites. We conclude that MKRN1, but not other 3’ UTR-binding proteins, binds upstream of poly(A) tails. 

The new analysis of eCLIP data are shown in the new Figure 3C.*

3) The authors state that "MKRN1 is positioned upstream of poly(A) sequences through direct interaction with PABP" (page 12, lanes 243-244). The strongest evidence for this is that the MKRN1-PAM2 mutant, which loses its interaction with PABP, also loses RNA binding. But it could also be that the structure of the PAM2 mutant is distorted so that it cannot bind RNA anymore. This is supported by the low levels of this mutant when over-expressed in cells (see also comment 4). Can the authors provide evidence that the MKRN1-PAM2 mutant is functional in other assays (e.g. ubiquitylation), and therefore is properly folded?

*We appreciate the Reviewer’s concern about the integrity of MKRN1PAM2mut. We had therefore chosen, in the first place, to inactivate the PAM2 motif by mutating three amino acid residues, rather than by deleting the complete motif as previously done (Miroci et al. 2012). 

In response to the raised concern, we now performed additional experiments to support that MKRN1PAM2mut is functional: 

1. We purified recombinant MKRN1 proteins from E. coli and performed in vitro ubiquitylation assays. We could show that MKRN1PAM2mut can autoubiquitylate with same efficiency as wild type protein. As a control, the ligase-dead MKRN1RINGmut is defective in these assays. 

The autoubiquitylation experiments are shown in the new Figure 1F.*

*2. We checked the subcellular localisation of GFP-tagged MKRN1wt and MKRN1PAM2mut in cells using confocal microscopy. We did not observed any aggregation of MKRN1PAM2mut nor any difference in the localisation of MKRN1PAM2mut compared to the wild type protein. 

Representative images are shown in the new Supplemental Figure S1D. 

3. In order to substantiate the impact of PABP on MKRN1 RNA binding, we performed in vitro RNA pulldown assays, in which we incubated recombinant PABPC1 and/or MKRN1 with bead-coupled RNAs with or without an A-stretch. We could show that PABPC1 strongly increased the pulldown of MKRN1 with the A20-harbouring RNA but not the control RNA. 

The in vitro RNA pulldown experiments are shown in the new Figure 3E. 

Together, these results provide evidence that the PAM2 mutation did not impair the overall integrity of MKRN1PAM2mut, but specifically abolished the PABP interaction in an otherwise functional protein.* 

4) Figure 1C: The PAM2 mutant is barely expressed, and this might be the reason why no signal is detected in the IP. A new experiment with reasonable expression of this mutant should be shown. Similarly, the "slight increase in PABPC1 binding" alluded to in page 7 (lanes 128-129) for the RING mutant could be due to the much higher expression of this protein and not to stabilization. Given the large differences in expression, a simple correction to input might be misleading in this case.

*In the original figure, the expression of MKRN1PAMmut was lower compared to wild type and MKRN1RINGmut. However, we did not observe a consistent trend that MKRN1PAM2mut is less expressed than the wild type protein in other experiments. We consistently see that MKRN1RINGmut is more expressed compared to wild type, which is probably due to the loss of autoubiquitylation and decrease in subsequent proteasome degradation. We have now replaced the GFP-MKRN1 AP with new AP in which the levels MKRN1PAM2mut are not anymore lower compared to MKRN1WT and MKRN1RINGmut (new Figure 1D). The new data more clearly shows that binding of MKRN1PAM2mut to PABPC1 is strongly compromised.* 

*We agree with the Reviewer that the slight increase in PABPC1 binding that we see for MKRN1RINGmut could result from differences in absolute protein levels. In fact, a follow-up with targeted Western blot experiments now pointed to a reduced interaction of MKRN1RINGmut and PABPC1 (shown in the new Figure 1D). We share the Reviewer’s concern that any fluctuations seen with this mutant could be a result of the substantially altered protein levels. We have made this clear in the Results section by adding the following sentence: 

“In Western blot experiments, we noted a reduced interaction of MKRN1RINGmut with PABPC1. A possible explanation could be the substantially increased levels of MKRN1RINGmut in the cells that may skew the normalisation in the Western blot experiments (Fig. 1D, Supplemental Fig. S3 and Supplemental Table S1).”*

5) Related with comment 3, the fact that A-stretches are close to MRNK1 sites does not mean that PABP and MKRN1 bind together to the mRNA, as stated along the manuscript. In addition, in the discussion the authors hypothesize a mutually dependent (cooperative?) binding of PABP and MKRN1 to short A-stretches. Gel mobility shift assays should help reinforce the author's hypothesis and will be instrumental for the conclusions.

*In order to substantiate the impact of PABP on MKRN1 RNA binding, we performed in vitro RNA pulldown assays, in which we incubated recombinant PABPC1 and/or MKRN1 with bead-coupled RNAs with or without an A-stretch. We could show that PABPC1 strongly increased the pulldown of MKRN1 with the A20-harbouring RNA but not the control RNA. 

The in vitro RNA pulldown experiments are shown in the new Figure 3E. 

As mentioned in our response to Minor comment #3 from Reviewer #1, we removed the comment on a possible effect of MKRN1 binding on PABPC1 binding from the manuscript, since further experimentation with RNAs harbouring short A-stretches would be required to support such a statement.* 

6) There are no changes in the levels of RPS10, PABPC1/C4, IGFBP1/2/3, ELAVL1 and MOV10 upon MKRN1 depletion (Sup Fig S8B). Why does a defect in ubiquitylation of these substrates not reflect at the total protein level? Why would these substrates show increased interaction with the MKRN1-RINGmut (Fig S7B) if their increased presence cannot be attributed to increased levels?

*The Reviewer is right that none of the ubiquitylation targets changes in abundance in total cell lysate of MKRN1 KD cells. In order to independently confirm these MS data, we now performed Western blots for PABPC1, RPS10, ELAVL1, LARP1, and Vinculin as a control in control and MKRN1 KD cells. 

This data is shown in the new Supplemental Figure S9C. 

Regarding the protein interactions of MKRN1RINGmut, we initially hypothesised that direct comparison of the MKRN1wt and MKRN1RINGmut interactomes would enable the identification of ubiquitylation substrates of MKRN1 (that might be stabilized in cells expressing MKRN1RINGmut). However, we agree with the Reviewer that such effects, if present, were potentially overshadowed by the higher levels of MKRN1RINGmut in cells, which resulted in higher amounts of the mutant protein being pulled down. We therefore decided to no longer refer to the MKRN1RINGmut interactome in the context of ubiquitylation target identification.* 

7) Can the authors show a Venn diagram depicting the degree of overlap of peaks and genes identified in the 3 MKRN1 iCLIP replicates?

*We now provide Venn diagrams showing the overlap of binding sites and target genes identified in three MKRN1 iCLIP replicates in new Supplementary Figure S4G.*

8) Please, explain the difference between Figure 2D and S4D. Note that there are several mistakes in the legend of Fig S4 (panel descriptions swapped or missed).

*In brief, we used two different metrics to capture the A enrichment downstream of the MKRN1 binding sites, namely A-rich stretches (>70% A) and continuous A-runs. For more details, please see our response to Minor comment #11 of Reviewer #1. 

As outlined above, we now changed the terminology to better delimit the two approaches. In addition, we corrected the legend in the revised version (now Supplemental Figure S5).* 

9) Fig 3D: Please, indicate that the bottom panel is a western-blot.

*We changed the figure accordingly.*

10) Page 9, lanes 164-166: It would be more correct to state "In accordance with our hypothesis that MKRN1 binds upstream of the poly(A) tail…".

*We have revised the text accordingly.*

11) Page 9, lanes 158-160: Eight adenosines is not the RNA footprint of PABP, but of one RRM of PABP. Please, correct.

*We have corrected this.*

12) Page 10 (lane 205): That the effects of MKRN1 and ZNF598 are not additive rather means that they work in the same pathway, reinforcing the model proposed by the authors.

*We agree with the Reviewer. We changed the sentence in the results section as follows: “Simultaneous depletion of MKRN1 and ZNF598 was not additive, indicating that both proteins might work in the same pathway”.*

13) Page 10 and Figure 4: Please, explain the stalling reporter as well as the principle of the assay in detail. One should not have to go to the indicated reference to understand it.

*We have now explained the principle of the ribosome stalling reporter assay in the Results section.*

14) Page 11, lane 228: Please, clarify in the main text that the interactions reported in this manuscript have been included.

*We changed this.*

15) Page 13, lane 267: This title should be tuned down by removing "to stall ribosomes" or by adding the word "model". 

*We have changed the title to “MKRN1 ubiquitylates RPS10 and PABPC1”.* 

16) The word "indicate" should be substituted by "suggest" in all instances where the author's conclusions are just inferences and no direct experimental data are provided. 

*We have done this throughout the manuscript.*

Reviewer #3: 

The manuscript by Hildebrandt and colleagues explores the role of the E3 ligase MKRN1 in ribosome-quality control. Prior work on the protein implicated it in a multitude of processes including control of telomere length, RNA polymerase II, cell cycle and regulation of p53 levels. The authors in this paper focus on one particular aspect of MKRN1's function in regulating translation through its interaction with polyA-binding protein (PABP). 

In the first set of experiments, they confirmed this interaction with mass-spec analysis as well as co-immunoprecipitation, and identified a PAM2 motif in MKRN1 responsible for interacting with PABP. In the second set of experiments, the authors went on to show that this interaction between MKRN1 and PABP could recruit the factor to polyA stretches in coding sequences. These first two set of experiments by and large served as confirmation for what others have reported in previous papers. 

*We agree that the interaction of MKRN1 and PABPC1 has been reported before, and we reference the respective studies several times. However, the finding that MKRN1 binds at poly(A) stretches of mRNA together with PABPC1, which is crucial for the present work, has not been reported in any previous study.* 

1) Arguably, the most interesting pieces of observations came from the third set of experiments, during which the authors showed a potential role for the factor in promoting ribosome stalling on polyA sequences and that its depletion results in reduction of ribosomal protein ubiquitination. Recent data have revealed that ubiquitination of ribosomal proteins of the small subunit by ZNF598 is important for ribosome rescue, RNA cleavage and degradation of the nascent peptide. The authors claim that MKRN1 acts upstream of ZNF598 to prevent the ribosome from translating polyA sequences by adding ubiquitin chains to the leading ribosome. While some of the observations made by the authors were interesting and could add to our understanding of ribosome-quality control, I felt that the authors made a huge leap coming up with their final model with the limited data presented. Many of the ideas were underdeveloped and the authors appear to have overinterpreted their data. For instance, the authors claim that MKRN1 is responsible for ubiquitinating ribosomal proteins based on the observation that ubiquitination is reduced when the protein is depleted. An alternative model is that the protein might be responsible for recruiting ZNF598 or another E3 ligase. 

*We have now performed in vitro ubiquitylation with recombinant MKRN1 purified from E. coli to demonstrate that MKRN1 can directly modify RPS10 and PABPC1 (see also Major comment #1 by Reviewer #1). The in vitro ubiquitylation data is presented in the new Figure 5G. 

In addition, we have performed a pulldown assay using GFP-MKRN1 and could not detect ZNF598 as interaction partner either by SILAC-AP-MS (Figure 1A, Supplementary Table 1) or by Western blots (new Figure 1D). The pulldown could recover PABPC1 as positive control. Similarly, we did not observe ZNF598 as a ubiquitylation target of MKRN1 in the ubiquitin remnant profiling. 

Although this provides a strong indication that MKRN1 and ZNF598 do not interact in cells, we cannot completely exclude this possibility. We have added in the results the following sentence: “We could not detect the interaction between MKRN1 and ZNF598 in pulldown experiments.”* 

2) Why the authors did not try to rescue this effect with different MKRN1 constructs is unclear. 

*We have now added complementation experiments in the revised manuscript. The specific impact of MKRN1 on ribosome stalling was supported by complementing the MKRN1 KD cells with stable integration of MKRN1WT, MKRN1PAMmut or MKRN1RINGmut constructs. Only MRKN1wt was capable of partially reverting the KD effect, such that ribosome stalling was restored, whereas neither of the MKRN1 mutants was functional in this assay.* 

*The complementation is shown in the new Figure 4D.* 

3) Furthermore, why the authors used siRNAs instead of CRISPR was not obvious. 

*We have now generated MKRN1 KO cells using CRISPR/Cas9 genome editing. The validation of these cells using Western blot and qPCR to monitor MKRN1 protein and mRNA levels, respectively, is shown in the new Supplemental Figure 8A,B. 

Similarly as in MKRN1 KD cells, we observed a decrease in ribosome stalling at poly(A) sequences in MKRN1 KO cells using the flow cytometry-based assay with the dual fluorescence reporter published by the Hedge Lab. 

The experiments in MKRN1 KO cells are shown in the new Figure 4C. 

We are convinced that our data with transient knockdown of MKRN1 is biologically highly relevant. Transient transfection of siRNA has some advantages over stable knockout of proteins using CRISPR/Cas9. For instance, stable knockout of MKRN1 (or any other protein) might lead to adaptation of cells and the dependency of cells in culture on redundant pathways. Indeed, we have observed a slight increase in MKRN2 mRNA levels in MKRN1 KO cells. For that reason, we believe that usage of transient siRNA-mediated knockdown and stable KO cell model systems is complementary and both biologically relevant.* 

4) The authors should also consider carrying out in vitro ubiquitination assays to show that MKRN1 is capable of adding ubiquitin chains to uS10. This is standard in the field, and earlier studies on ZNF598 used these sort of analyses to assign a function for the protein. 

*We have now performed in vitro ubiquitylation with recombinant MKRN1 purified from E. coli to demonstrate that MKRN1 can directly modify RPS10 and PABC1. 

The in vitro ubiquitylation data is presented in the new Figure 5G.* 

5) The authors should also consider validating some of their mass spec analysis using western blotting on in vivo targets. 

*We pulled down ubiquitylated proteins from cells expressing tagged ubiquitin, but failed to convincingly detect ubiquitylation of endogenous RPS10 by Western blot under any condition. This included combinations of DTT treatment and ZNF598 KD, as reported for instance in Juszkiewicz & Hegde, 2017. A possible explanation is that only a minor pool of ribosomes is stalled at poly(A) sequences. 

However, as outlined above, we now performed in vitro ubiquitylation assays with MKRN1 and RPS10 (new Figure 5G). Together with in vivo ubiquitin remnant profiling data, this strongly supports our hypothesis that MKRN1 can directly ubiquitylate RPS10.* 

6) Overall, while the study has the potential of adding to the emerging field of ribosome ubiquitination and mRNA-quality control, in its current form the paper falls short of being appropriate for Genome Biology 

Other issues: 

1) The authors provided data that showed depletion of MKRN1 by siRNAs leads to an increase in translation of a polyA-containing reporter and claim that this derepression is similar to what others observed for ZNF598 knockdown. Others have reported almost complete derepression, which is not the case for MKRN1 knockdown, which shows 2 to 3 fold derepression at best. 

*In our study, we use the same reporter constructs and analysis strategy as reported in Juszkiewicz & Hegde, 2017 and Sundaramoorthy et al. 2017 (kindly provided by Ramanujan S. Hegde). In both publications, the observed derepression upon ZNF598 knockdown is around 4-fold (see Figure 2A in Juszkiewicz & Hegde, 2017 below). In our experiments, we detect a comparable effect size of derepression, both for ZNF598 KD (4.1-fold) as well as for MKRN1 KD2 (3.6-fold). 

The seemingly modest effect can most likely be attributed to frameshifts at sequential AAA codons. This has been addressed in Juszkiewicz & Hegde, 2017 as follows: 
“Because of possible frameshifting at sequential AAA codons (Arthur et al., 2015; Koutmou et al., 2015), the RFP:GFP ratio is likely to underestimate the overall level of read-through. When correcting for this effect by quantifying RFP production in each reading frame, we can deduce that (KAAA)12 and (KAAA)20 show ~10% and ~60% stalling, respectively (Figures S1A and S1B).” 

[copied from Juszkiewicz & Hegde, 2017, page 745] 

“Knockdown of ZNF598 in the stable cell line expressing the (KAAA)21 construct showed an increased RFP:GFP ratio (Figure 2A) and increased levels of full-length FLAG-SR-K21 (Figure 2B), indicating read-through of the poly(A) region. After compensating the RFP signal for frameshifting that occurs during poly(A) translation (Figures S2A and S2B), we estimate that read-through of (KAAA)21 in the absence of ZNF598 is ~90% of that seen for the (K)0 construct. Thus, almost no terminal stalling at (KAAA)21 occurs in ZNF598 knockdown cells.” 

[copied from Juszkiewicz & Hegde, 2017, page 745] 

We decided to not correct our measurements for frameshifting, but rather to present the raw results, as it is also done in the main figures of the two publications.* 

2) Is MKRN1 associated with polysomes? This is important to assess since PABP is. 

*We have now tested whether MKRN1 is associated with polysomes using sucrose gradient centrifugation experiments. MKRN1 was clearly present in polysomal fractions and co-sedimented with PABPC1, indicating that together with PABPC1, MKRN1 is associated with translating ribosomes. 

The polysome fractionation data is shown in the new Figure 1B.* 

3) qPCR or northern analysis of the stalling reporter should be conducted to assess the effect of the factor on no-go decay. 

*We have now performed qPCR on the stalling reporter mRNA as suggested by the Reviewer. The mRNA levels were not significantly affected in MKRN1 KD cells. This is in agreement with the previous study from the Hegde lab (Juszkiewicz & Hegde, 2017) in which the authors did not observe a change of GFP levels in (K)0 versus (KAAA)21 reporter-expressing HEK293 cells.* 

4) Figure 1C, the claim that PAM2mut does not interact with PABP is not convincing since that particular construct is hardly expressed. The quantification in panel D does not appear to show significant difference between the wild type and the mutant. 

*See Major comment #4 by Reviewer #1 above. 

In original figure, the expression of MKRN1PAMmut was lower compared to wild type and MKRN1RINGmut. However, the reduced expression of MKRN1PAM2mut compared to the wild type protein was not a consistent trend in other experiments. However, we repeatedly saw that MKRN1RINGmut is more expressed compared to wild type and MKRN1PAM2mut, which is probably due to the loss of autoubiquitylation and decrease in subsequent proteasome degradation. 

In order to level this confounding effect, we have now replaced the experiment in Figure 1D with a new GFP-MKRN1 AP in which the levels of MKRN1PAM2mut are not lower compared to MKRN1WT (new Figure 1E). The new data more clearly shows that binding of MKRN1PAM2mut to PABPC1 is strongly compromised.*

**Second round of review**

**Reviewer 1**

I am happy to recommend publication of the revised MS, which will be of wide interest in the field of translational quality control.

**Reviewer 2**

The authors have adequately addressed my concerns and I have no further comments except for:
1) Please, pay attention to mention the Figures correctly in the main text. There are several mistakes (e.g. when referring to Figure 4 and Figs S6-S8 in page 12).
2) Regarding the new panels of Figure 4, the changes in MKRN1 KO cells (Fig 4C) are very small compared to WT cells. Therefore, I think the sentence "Reporter assays confirmed that complete loss of MKRN1 impaired ribosome stalling at K(AAA)20" is too strong. Similarly, in Fig 4D, adding wt MKRN1 only partially restores ribosome stalling, as the authors correctly mention.

**Reviewer 3**

The revised manuscript is much better, and I appreciate the fact that the authors spent a lot of effort trying to address my concerns and those of the other reviewers. The new in vitro ubiquitination data adds support to the authors’ model. 

I still have some issues with some of the arguments made by the authors:

1)     I was curious as to why the authors did not include the light fractions when analyzing MRKN1 association with the polysomes. 
2)     Could the author explain why they used purified uS10 for the in vitro ubiquitination assays? Why not use ribosomes? Also, could they show that this is specific to uS10? Why did not they show that in vivo uS10 is not ubiquitinated in the RING mutant background as reviewer asked?
3)     Figure 4B, why is the effect of MKRN1 knockdown (siRNA1) with ZNf598 knockdown smaller than ZNf598 by itself? 
4) The knockdown appears to have a larger effect on the drepressing the K20 reporter relative to the knockout. This doesn't make sense. Also, the complementation is not convincing.

**Authors Response**

**Point-by-point responses to the reviewers’ comments:**

We would like to thank the Reviewers for their comments and suggestions during the reviewing process, which helped to improve our manuscript. 

Reviewer #1: 

I am happy to recommend publication of the revised MS, which will be of wide interest in the field of translational quality control. 

The authors have done a good job in addressing the initial comments. 

*We appreciate the Reviewer’s support.*

Reviewer #2: 

The authors have adequately addressed my concerns and I have no further comments except for: 

1) Please, pay attention to mention the Figures correctly in the main text. There are several mistakes (e.g. when referring to Figure 4 and Figs S6-S8 in page 12). 

*We apologise for the mistakes. We carefully checked all figure references throughout the manuscript again.* 

2) Regarding the new panels of Figure 4, the changes in MKRN1 KO cells (Fig 4C) are very small compared to WT cells. Therefore, I think the sentence "Reporter assays confirmed that complete loss of MKRN1 impaired ribosome stalling at K(AAA)20" is too strong. Similarly, in Fig 4D, adding wt MKRN1 only partially restores ribosome stalling, as the authors correctly mention. 

*In the revised version, we now describe in more detail the observed effects and provide possible explanations for the small effect size compared to the KD (see also comment 4 by Reviewer #3 below). The respective paragraph now reads: 
“For independent validation of the KD results, we generated a stable MKRN1 knockout (KO) cell line using CRISPR/Cas9 genome editing (Additional file 1: Fig. S8C,D). Reporter assays showed that complete loss of MKRN1 impaired ribosome stalling at K(AAA)20, albeit with a small effect size compared to the siRNA-mediated KD (Fig. 4D and Additional file 1: Fig. S7C). qPCR indicated a compensatory upregulation of the paralog MKRN2 in the MKRN1 KO but not in the MKRN1 KD (Additional file 1: Fig. S8B,D), which could explain the reduced effect size of the MKRN1 KO in the reporter assays. In line with a partially redundant role of MKRN2, we find that simultaneous depletion of MKRN1 and MKRN2, as observed upon KD with siRNA2 (Additional file 1: Fig. S8B), shows a larger effect than KD with siRNA1, which does not change MKRN2 levels.”*

Reviewer #3: 

The revised manuscript is much better, and I appreciate the fact that the authors spent a lot of effort trying to address my concerns and those of the other reviewers. The new in vitro ubiquitination data adds support to the authors’ model. 

I still have some issues with some of the arguments made by the authors: 

1) I was curious as to why the authors did not include the light fractions when analyzing MRKN1 association with the polysomes. 

*The lightest fractions of the gradient, corresponding to the free mRNAs, were not included in the analyses as these proved to be highly variable with respect to their RNA content in the past. This is at least in part due to technical constraints in the fractionation process leading to a higher variability in the exact volume at this end of the gradient. Moreover, as our main goal was to determine whether MRKN1 associates with translating ribosomes, we primarily focused on the heavier fractions, in line with a recent methods paper (Pringle et al. 2019 Polysome profiling analysis of mRNA and associated proteins engaged in translation. Curr Protoc Mol Biol, 125, e79.).* 

2) Could the author explain why they used purified uS10 for the in vitro ubiquitination assays? Why not use ribosomes? Also, could they show that this is specific to uS10? Why did not they show that in vivo uS10 is not ubiquitinated in the RING mutant background as reviewer asked? 

*We identified RPS10 (eS10) as a putative substrate of MKRN1 in ubiquitin remnant profiling experiments and therefore decided to use recombinant RPS10 purified from E. coli in in vitro ubiquitylation assays. Purified ribosomes from mammalian cell lysate, as used for instance in Juszkiewicz & Hegde, Mol Cell, 2017, may contain impurities that could contribute to or interfere with the in vitro ubiquitylation reaction. In future experiments, it will be interesting to reconstitute the complete stalling complex, including ribosomes and prematurely polyadenylated mRNAs, but this is beyond the scope of the current manuscript. 
As outlined in the first round of revision, we pulled down ubiquitylated proteins from cells expressing tagged ubiquitin, but failed to convincingly detect ubiquitylation of endogenous RPS10 by Western blot under any condition. A possible explanation is that only a minor pool of ribosomes is stalled at poly(A) sequences.* 

3) Figure 4B, why is the effect of MKRN1 knockdown (siRNA1) with ZNf598 knockdown smaller than ZNf598 by itself? 

*The difference between the MKRN1 (siRNA1) & ZNF598 double knockdown and the ZNF598 single knockdown is not significant (P value = 0.6302, paired two-tailed Student’s t-test). Residual differences are difficult to assess due to the variability between measurements.* 

4) The knockdown appears to have a larger effect on the drepressing the K20 reporter relative to the knockout. This doesn't make sense. Also, the complementation is not convincing. 

*The Reviewer is correct that the siRNA-mediated knockdown (KD) shows a stronger effect in our reporter measurements than the CRISPR/Cas9 knockout (KO). Our results indicate that this may result from the upregulation of the paralog MKRN2, which occurs in the MKRN1 KO but is not detected in the KD (see Additional file 1: Fig. S8B,D). In line with a partial compensatory role of MKRN2, we find that simultaneous depletion of MKRN1 and MKRN2, as observed upon KD with siRNA2 (see Additional file 1: Fig. S8B), shows a larger effect than KD with siRNA1, which does not change MKRN2 levels. 
In order to clarify this in the text, we extended the respective paragraph as follows: 
“For independent validation of the KD results, we generated a stable MKRN1 knockout (KO) cell line using CRISPR/Cas9 genome editing (Additional file 1: Fig. S8C,D). Reporter assays showed that complete loss of MKRN1 impaired ribosome stalling at K(AAA)20, albeit with a small effect size compared to the siRNA-mediated KD (Fig. 4D and Additional file 1: Fig. S7C). qPCR indicated a compensatory upregulation of the paralog MKRN2 in the MKRN1 KO but not in the MKRN1 KD (Additional file 1: Fig. S8B,D), which could explain the reduced effect size of the MKRN1 KO in the reporter assays. In line with a partially redundant role of MKRN2, we find that simultaneous depletion of MKRN1 and MKRN2, as observed upon KD with siRNA2 (Additional file 1: Fig. S8B), shows a larger effect than KD with siRNA1, which does not change MKRN2 levels.”*
